# The function of LncRNAs and their role in the prediction, diagnosis, and prognosis of lung cancer

**DOI:** 10.1002/ctm2.367

**Published:** 2021-04-05

**Authors:** Yu Chen, Emory Zitello, Rui Guo, Youping Deng

**Affiliations:** ^1^ Department of Quantitative Health Sciences John A. Burns School of Medicine, University of Hawaii at Manoa Honolulu Hawaii USA; ^2^ Department of Molecular Biosciences and Bioengineering, College of Tropical Agriculture and Human Resources University of Hawaii at Manoa Honolulu Hawaii USA; ^3^ School of Public Health Guangxi Medical University Nanning China

**Keywords:** biomarker, chemosensitivity, diagnosis, EGFR, LDCT, lncRNA, lung cancer, predictive, prognosis, radiosensitivity

## Abstract

Lung cancer remains a major threat to human health. Low dose CT scan (LDCT) has become the main method of early screening for lung cancer due to the low sensitivity of chest X‐ray. However, LDCT not only has a high false positive rate, but also entails risks of overdiagnosis and cumulative radiation exposure. In addition, cumulative radiation by LDCT screening and subsequent follow‐up can increase the risk of lung cancer. Many studies have shown that long noncoding RNAs (lncRNAs) remain stable in blood, and profiling of blood has the advantages of being noninvasive, readily accessible and inexpensive. Serum or plasma assay of lncRNAs in blood can be used as a novel detection method to assist LDCT while improving the accuracy of early lung cancer screening. LncRNAs can participate in the regulation of various biological processes. A large number of researches have reported that lncRNAs are key regulators involved in the progression of human cancers through multiple action models. Especially, some lncRNAs can affect various hallmarks of lung cancer. In addition to their diagnostic value, lncRNAs also possess promising potential in other clinical applications toward lung cancer. LncRNAs can be used as predictive markers for chemosensitivity, radiosensitivity, and sensitivity to epidermal growth factor receptor (EGFR)‐targeted therapy, and as well markers of prognosis. Different lncRNAs have been implicated to regulate chemosensitivity, radiosensitivity, and sensitivity to EGFR‐targeted therapy through diverse mechanisms. Although many challenges need to be addressed in the future, lncRNAs have bright prospects as an adjunct to radiographic methods in the clinical management of lung cancer.

## Graphical abstract

Serum or plasma assay of lncRNAs in blood can be used as a novel detection method to assist low dose CT scan (LDCT) while improving the accuracy of early lung cancer screening. LncRNAs can be used as predictive markers for chemosensitivity, radiosensitivity, and sensitivity to epidermal growth factor receptor (EGFR)‐targeted therapy, and as well markers of prognosis. Different lncRNAs have been implicated to regulate chemosensitivity, radiosensitivity and sensitivity to EGFR‐targeted therapy through diverse mechanisms.

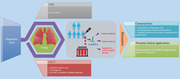

**Abbreviations**
ABC: ATP‐binding cassette
ABCG2: ATP binding cassette subfamily G member 2
ABHD11‐AS1: ABHD11 antisense RNA 1
ANRIL: antisense noncoding RNA in the INK4 locus
ATG: autophagy‐related
ATG7: target autophagy‐related 7
AUC: area under the curve
BLACAT1: bladder cancer‐associated transcript 1
CAPG: Capping Actin Protein, Gelsolin Like
CAR10: chromatin‐associated RNA 10
CARM1: coactivator‐associated arginine methyltransferase 1
CEA: carcinoembryonic antigen
ceRNAs: competing endogenous RNAs
CRC: colorectal cancer
CSCs: Cancer stem cells
CXR: chest X‐ray
CYFRA 21‐1: cytokeratin 19‐fragments
CYTOR: cytoskeleton regulator RNA
EGF: epidermal growth factor
EGFR: epidermal growth factor receptor
EGFR‐TKIs: EGFR tyrosine kinase inhibitors
EMT: epithelial‐to‐mesenchymal transition
eRNAs: enhancer RNAs
EZH2: Enhancer of Zeste homolog 2
FAM201A: family with sequence similarity 201 member A
FENDRR: FOXF1 adjacent noncoding developmental regulatory RNA
GAS5: growth arrest‐specific 5
HIF‐1α: hypoxia‐inducible factor 1alpha
HOST2: human ovarian cancer‐specific transcript 2
HOTAIR: HOX transcript antisense RNA
HOTAIRM1: HOXA transcript antisense RNA myeloid‐specific 1
HOXA10: homeobox A10
HOXA11‐AS: HOXA11 antisense RNA
KCNQ1OT1: KCNQ1 opposite strand/antisense transcript 1
LAD: lung adenocarcinoma
LDCT: low dose CT scan
LINC02418: long intergenic non‐protein coding RNA 2418
LincRNA‐p21: long intergenic noncoding RNA‐p21
lincRNA‐ROR: long intergenic non‐protein coding RNA, regulator of reprogramming
LincRNAs: long intergenic noncoding RNAs
LncRNAs: long noncoding RNAs
LUSC: lung squamous cell carcinoma
m^6^A: N^6^‐methyladenosine
MALAT1: metastasis associated in lung adenocarcinoma transcript 1
MBNL3: muscleblind‐like 3
MDR: multidrug resistance
MDR1: multidrug resistance protein 1
MEG3: maternally expressed 3; METTL3, methyltransferase‐like 3
METTL14: methyltransferase‐like 14
MIR4435‐2HG: MIR4435‐2 host gene
miRNAs: microRNAs
MMP‐2: matrix metallopeptidase 2
MMP‐9: matrix metallopeptidase 9
MRP1: multidrug resistance‐associated protein 1
MRP7: multidrug resistance‐associated protein 7
NCHS: National Center for Health Statistics
NCN: noncalcified nodules
ncRNA: noncoding RNA
NEAT1: nuclear paraspeckle assembly transcript 1
NNT‐AS1: NNT antisense RNA 1
NSCLC: non‐small cell carcinoma
NSE: neuron‐specific enolase
ProGRP: progastrin‐releasing peptide
PVT1: plasmacytoma variant translocation 1
SBF2‐AS1: SBF2 antisense RNA 1
SCCA: squamous cancer cell antigen
SCLC: small cell lung carcinoma
SNHG12: small nucleolar RNA host gene 12
SOX2OT: SOX2 overlapping transcript
TNM: tumor node metastasis
USP7: ubiquitin specific peptidase 7
XIST: X‐inactive‐specific transcript
YAP: Yes‐associated protein

## 1 INTRODUCTION

Lung cancer is a malignancy that originates in the bronchial mucosa or glands of the lungs. As cancer cells grow and spread, they severely damage the patient's respiratory system and compromise oxygen exchange.

In 2018, the World Health Organization surveyed the morbidity and mortality of 36 types of cancer in 185 countries or regions. In terms of morbidity and mortality, the top three cancers were lung cancer, breast cancer, and prostate cancer. Specifically, in both sexes combined, the number of patients diagnosed with lung cancer was 2,093,876 (11.6% of all cancer cases). The number of patients dying from lung cancer was 1,761,007 (18.4% of all cancer deaths).^1^ This shows that lung cancer still poses a serious threat to human health worldwide.

In the United States, cancer is a major disease and the second leading cause of death. A generalized linear mixed model was used by the National Center for Health Statistics to predict the total number of invasive cancer cases that will occur in the United States in 2020. It estimates that there will be 1,806,590 new cases of confirmed cancer and 606,520 new cases of cancer deaths in the United States throughout 2020. Among them, this will add 228,820 cases and 135,720 deaths for lung and bronchial cancers.

Traditionally, lung cancers comprise two subtypes: non‐small cell carcinoma (NSCLC) and small cell lung carcinoma (SCLC). SCLC is relatively uncommon, accounting for 20% of the total number of lung cancer patients. SCLC grows faster, and extensive metastasis occurs early, making it a highly malignant tumor. When patients are initially diagnosed, lung cancer cells have often metastasized, making cure difficult. For untreated patients, the median survival time is 2‐4 months. SCLC is extremely sensitive to chemotherapy; however, even after chemotherapy, patients will often relapse within 2 years of treatment and suffer significant side effects. Among patients with distant metastases, the 2‐year survival rate is less than 10%.^2^, ^3^

NSCLC is more common, accounting for 80% of the total number of lung cancer patients. Actually, the separation of histological subtypes is of great significance for the clinical treatment of NSCLC. NSCLC mainly includes lung adenocarcinoma (LAD), lung squamous cell carcinoma (LUSC), and large cell carcinoma. Compared with SCLC, NSCLC usually grows and spreads more slowly. For patients diagnosed with early stage NSCLC, tumors can usually be resected surgically. When the tumor has metastasized locally, it is treated by the simultaneous use of radiotherapy and chemotherapy. In cases when the tumor has been found to have metastasized to distant sites, palliative chemotherapy is generally the only available option. Many NSCLC patients are not diagnosed until advanced‐stage disease is present. The 5‐year relative survival rate of patients with NSCLC is only 15%.^3^, ^4^

In cases with either advanced SCLC or NSCLC, the prognosis is poor, and the survival rate is low. Therefore, as with many other cancer types, early detection of lung cancer is very important for overall patient prognosis.

This article discusses the advantages and disadvantages of low dose CT scan (LDCT) and chest X‐ray (CXR) for early detection of lung cancer. We relate this to the various functions of long noncoding RNAs (lncRNAs) and their potential role as emerging molecular markers in the prediction, diagnosis, and prognosis of lung cancer.

## 2 THE ADVANTAGES AND DISADVANTAGES OF LDCT AND CXR FOR EARLY DETECTION OF LUNG CANCER

LDCT and CXR are the two main methods of early detection of lung cancer. In France, from October 2002 to December 2004, the Depiscan study was carried out in order to compare the sensitivity of LDCT and CXR in early lung cancer screening.^5^ The size of noncalcified nodules (NCN) was measured through LDCT and CXR in this study. Statistical analysis of the test results showed that the probability of NCN detected by LDCT was higher than that of CXR. Additionally, LDCT can detect NCN with very small diameters that CXR cannot detect. Therefore, patients can be screened earlier, thereby increasing the probability of cure.^6^ For a study in Japan, it also showed that compared with CXR, the probability of detecting lung cancer by LDCT is nearly 10 times higher.^7^ On the basis of data such as that above, it has been determined that LDCT is more sensitive than CXR in detecting the lung cancer at an early stage.

Although LDCT has obvious advantages for early lung cancer screening compared with CXR, it also has some disadvantages. The National Lung Screening Trial (NLST) in USA was carried out from August 2002 to April 2004. A total of 53,454 high‐risk patients with lung cancer were randomly scheduled for 3‐year annual screenings with either LDCT or CXR. Although LDCT screening can reduce lung cancer mortality, 96.4% of the positive screening results in the LDCT group and 94.5% of the positive screening results in the CXR group were false positive results.^8^ Because these results are typical of large population screens for lung cancer, these approaches for early detection are not suitable for routine clinical applications.

**HIGHLIGHTS**

- Although LDCT has some disadvantages, it is still the main method for early detection of lung cancer.
- LncRNAs can be used as novel predictive/diagnostic/prognostic markers to assist LDCT while improving the accuracy of early lung cancer screening.
- Different lncRNAs have been implicated to regulate chemosensitivity, radiosensitivity, and sensitivity to EGFR‐targeted therapy through diverse mechanisms in lung cancer.

In addition to higher false positives, LDCT has two adverse effects that may be a cause of concern among clinicians. Overdiagnosis typically leads to one of the following two outcomes: firstly, a misdiagnosed lesion will not deteriorate further (or will subside), or secondly, the patient may die from another disease before the cancer progresses.^9^ Another disadvantage of LDCT is that even while reducing the mortality of lung cancer, it may increase the risk of cancer caused by radiation exposure. Early diagnosis of lung cancer through LDCT requires regular follow‐up of subjects to track the development of pulmonary nodules, which may entail its own independent risk of lung cancer. Two different time intervals of 20 and of 30 years were used to evaluate the accumulation of radiation caused by LDCT. In the 20‐, 30‐year program, the cumulative radiation exposure to the subject can reach 160 mSv and 212 mSv, respectively. Radiation effects are cumulative. The risk of cancer occurrence increases according to a linear dose‐response relationship.^10^ What's more, according to the results of ITALUNG trial of lung cancer screening reported by Mascalchi et al, 77.4% of radiation exposure is a result of annual screening, and 22.6% is due to the follow‐up of abnormal LDCT scans. The incidence of radiation‐induced lung cancer ranges from 0.12 to 0.33 per 1000 subjects.^11^

The radiation exposure that usually garners attention is mainly that emanating from nuclear power stations. In a study involving shipyard workers in the US nuclear power overhauls between 1957 and 1982, compared with workers exposed to 5.0–9.9 mSv radiation, workers exposed to 50 mSv radiation had a higher risk of lung cancer (1.26; 95% CI [Confidence interval], 0.9–1.9).^12^ However, the data on the high cumulative dose radiation exposure of nuclear workers are limited compared with the radiation accumulation data of LDCT screening and subsequent follow‐up, because nuclear power stations are under strict supervision, and nuclear workers rarely receive cumulative radiation exceeding 100 mSv.^10^ A publication on the subject by Boice et al mentioned that 5801 people participated in radiation‐exposing activities from 1948 to 1999 and reported a long‐term external average radiation dose of 13.5 mSv. The detected long‐term external and internal average radiation dose was estimated at 19 mSv.^13^ Jeong et al conducted a statistical analysis of the radiation exposure of 8429 nuclear workers at nuclear power plants in South Korea from 1992 to 2005. Only 2182 people had an estimated internal radiation dose greater than 0, and the long‐term average radiation dose was 0.82 mSv.^14^

It can be seen that the amount of radiation accumulated by LDCT screening and subsequent follow‐up far exceed even that of nuclear workers. In addition, radiation accumulated by LDCT screening and subsequent follow‐up can increase the risk of lung cancer.

## 3 THE IMPORTANCE OF LIQUID BIOPSY

Currently, LDCT is still the main method for early detection of lung cancer. However, due to its disadvantages (high false positive rate, overdiagnosis and the risk of cumulative radiation exposure), it is desirable to find noninvasive and more conveniently assayed detection methods as an auxiliary to LDCT in order to improve the accuracy of early detection of lung cancer.^15^

The occurrence and development of lung cancer is a multi‐step, gradual process. This process, known as carcinogenesis, is accompanied by the accumulation of genetic and epigenetic abnormalities, ultimately leads to the process of abnormal growth of normal lung epithelial cells, especially for LUSC.^16^, ^17^, ^18^, ^19^, ^20^ Molecular lesions usually occur in the normal‐looking lung tracheal epithelium, with no initial atypical hyperplasia. Metaplasia, which refers to a process in which tissues or cells that have been differentiated and matured are transformed into another form (i.e., homologous nature) to adapt to the environmental stresses under the stimulation of certain factors, is typical of precancerous lesions. Although metaplasia is a positive response made by tissues or cells in order to resist some external stimuli, the function of the original tissues or cells is lost because of the change in tissue morphology. Over time, metaplastic tissues have a tendency to become cancerous. A large proportion of lung cancer patients have a long history of smoking. The metaplasia caused by smoking is accompanied by genetic abnormalities. This metaplasia is regarded as a precancerous lesion. When genetic abnormalities accumulate, it may cause uncontrolled cell growth and migration and facilitates tumor growth and metastasis. The activation of oncogenes or the inactivation of anti‐oncogenes may be related to genetic modifications, including mutations, epigenetic modifications, etc. In general, molecular lesions begin much earlier than pathological morphological changes.^21^

Tissue biopsy is generally highly invasive. However, liquid biopsy has the advantage of being noninvasive and well‐tolerated by patients.^22^ With the rapid development of modern molecular technology, circulating nucleic acids can be extracted from serum or plasma. Using a Polymerase chain reaction (PCR) protocol, tumor‐derived circulating nucleic acids can be detected as an adjunct diagnostic tool to LDCT to carry out early detection of lung cancer, thus improving the accuracy of diagnosis.^23^

## 4 WHAT ARE LncRNAs?

In 1961, Crick et al proposed the central dogma of molecular biology, and RNA was regarded as the link between DNA and protein.^24^ However, in the human genome, most genes do not encode proteins, and the percentage of noncoding RNAs (ncRNAs) is greater than or equal to 98%. RNA without protein coding function is called ncRNA.^25^

LncRNAs comprise a very important class of circulating nucleic acids. They are a type of ncRNAs with a length of 200–100,000 nucleotides.^26^ According to the relative position between lncRNAs and proteincoding transcripts, lncRNAs are divided into the following two types: genic lncRNAs and long intergenic noncoding RNAs (lincRNAs).^27^ Based on the direction of gene transcription, the genic lncRNAs include the following two subtypes: sense lncRNAs and antisense lncRNAs.^28^

## 5 THE FUNCTION OF LncRNAs

LncRNAs are a very important part of the epigenetic regulatory network. LncRNAs can regulate epigenetic gene expression by affecting the structure of chromatin,^29^, ^30^, ^31^ histone modification,^32^, ^33^, ^34^ alternative splicing,^35^, ^36^ X‐chromosome silencing,^37^ and dosage compensation.^38^ Although lncRNAs cannot encode translated proteins, they can participate in the transcription process by regulating promoters,^39^ enhancers,^40^, ^41^ and transcription factors.^42^, ^43^ In addition, lncRNAs affect post‐transcriptional regulation in a manner that serves as precursors to small RNAs^44^, ^45^ and stabilizes mRNAs.^46^, ^47^ LncRNAs can also be regarded as competing endogenous RNAs (ceRNAs) to sponge microRNAs (miRNAs) to affect the expression of related downstream target genes.^48^, ^49^ Table S1 shows diversified biological functions of lncRNAs.

## 6 LncRNAs IN HUMAN CANCERS

The following eight hallmarks have been described in human cancers: 1) maintenance of proliferation, 2) replicative immortality, 3) antagonism of cell death, 4) avoiding growth inhibitors, 5) metabolic reprogramming, 6) promotion of angiogenesis, 7) activation of invasion and metastasis, and 8) evasion of immunosurveillance.^50^ Especially, some lncRNAs can affect various hallmarks of lung cancer. As examples, 1) upregulated SBF2 antisense RNA 1 (SBF2‐AS1) and SOX2 overlapping transcript (SOX2OT) promote the proliferation of lung cancer cells,^51^, ^52^ 2) long intergenic noncoding RNA‐p21 (lincRNA‐p21) modulates angiogenesis to affect the prognosis of patients with surgically resected lung cancer,^53^ 3) metastasis‐associated lung adenocarcinoma transcript 1 (MALAT1) and chromatin‐associated RNA 10 (CAR10) both play an important role in the metastasis of lung cancer,^54^, ^55^ and 4) downregulated HOXA transcript antisense RNA myeloid‐specific 1 (HOTAIRM1) is capable of promoting evasion of immunosurveillance in lung cancer patients.^56^

As mentioned earlier, thousands of studies have concluded that lncRNAs are key regulators involved in the progression of human cancers through four main multiple action models as follows: 1) epigenetic regulation, 2) transcriptional and post‐transcriptional regulation, 3) acting as oncogene or tumor suppressor, and 4) serving as enhancer RNAs (eRNAs) and ceRNAs.^57^

## 7 POTENTIAL CLINICAL APPLICATIONS OF LncRNAs AS BIOMARKERS IN LUNG CANCER

### 7.1 LncRNAs as predictive markers in lung cancer

#### 7.1.1 Chemosensitivity

Prior to the invasion or metastasis of cancer cells, surgical removal of the primary tumor in suitable surgical candidates with early‐stage NSCLC is usually possible. Unfortunately, about 30%–35% of patients with stage I NSCLC will relapse after surgery. Clinical statistics on adjuvant chemotherapy after NSCLC resection showed a 5‐year relative survival rate of 44.5% among 1867 subjects who received chemotherapy, which was significantly higher than 40.4% in the control group. This study also demonstrated that adjuvant chemotherapy after surgical resection can reduce the deaths of 7000 NSCLC patients each year. Several other clinical trials have reported on the survival of NSCLC patients of stages II and IIIA. After adjuvant cisplatin‐based chemotherapy, 5‐year relative survival rate increased by 8%–15%.^58^, ^59^ Therefore, after surgical resection, adjuvant chemotherapy has been conclusively proven to be of benefit to patients with early‐stage lung cancer. Unfortunately, acquired chemoresistance of lung cancer during treatment is one of the main reasons for the poor treatment efficacy in NSCLC. LncRNAs have been explored as a very promising predictive marker for chemosensitivity, which may potentially assist in the selection of the most suitable treatment for patients with lung cancer.^60^ Table S2 summarizes some lncRNAs suggested as predictive markers that may have an impact on chemosensitivity in lung cancer.

##### Signaling pathways related to chemosensitivity

After surgical removal of tumor, chemotherapy is regarded as an important adjuvant treatment for many patients with NSCLC. However, during a course of chemotherapy, patients often develop resistance to drugs such as cisplatin and paclitaxel.^4^ Knowledge of the underlying mechanisms of chemotherapy resistance is therefore essential to improve the efficacy of chemotherapy.

**FIGURE 1:**
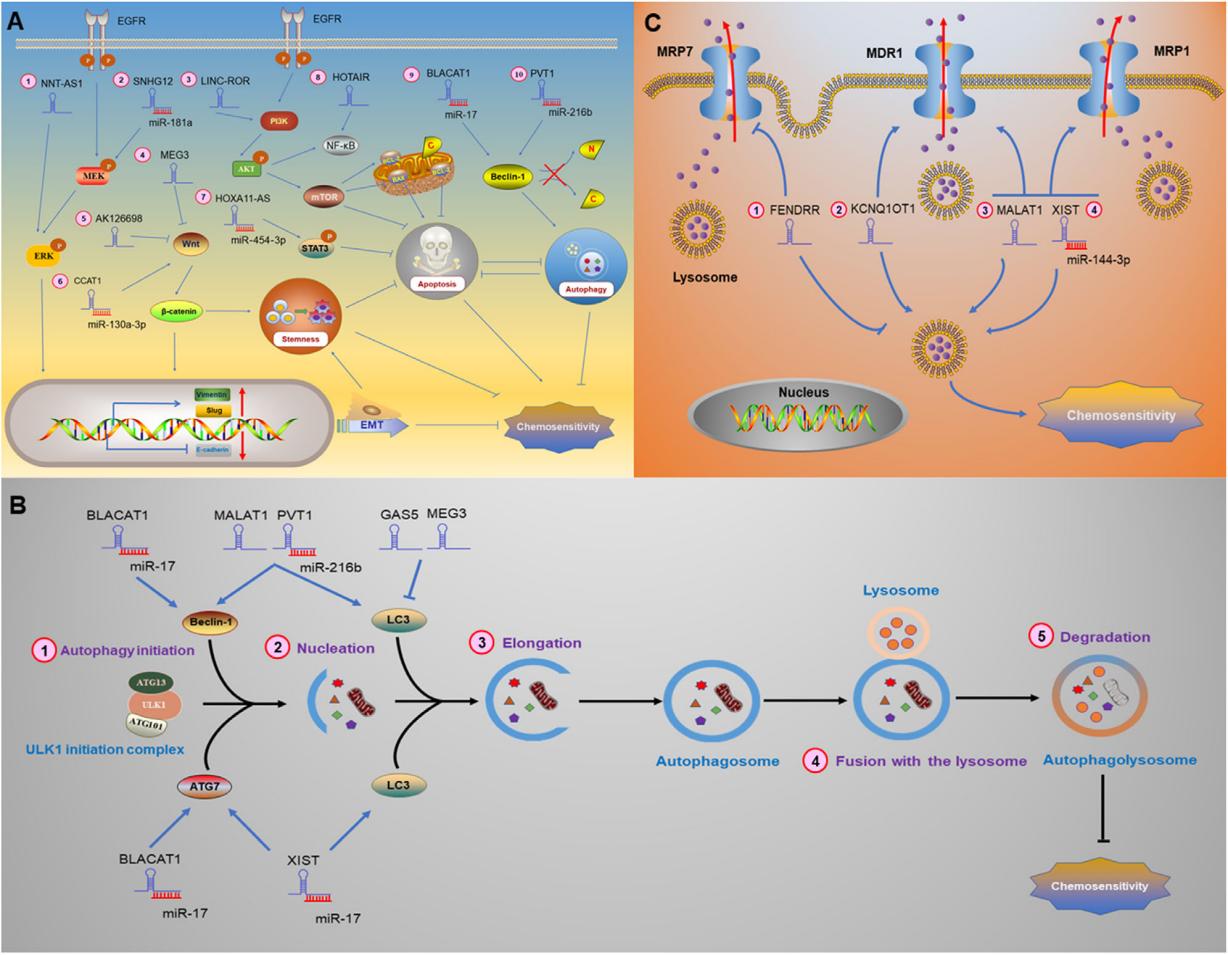
In lung cancer, lncRNAs regulate chemosensitivity through various mechanisms. A, LncRNAs regulate chemosensitivity through different signaling pathways. P: phosphorylated.①② LncRNAs regulate EMT‐mediated chemosensitivity by MAPK/ERK signaling pathways.④⑤⑥ LncRNAs regulate cancer cell stemness, EMT and chemosensitivity by regulating Wnt/β‐catenin signaling pathway. ③⑧ LncRNAs regulate chemosensitivity by modulating PI3K/AKT/mTOR and NF‐κB signaling pathway.⑦ LncRNAs regulate chemosensitivity through modulating STAT3 signaling pathway.⑨⑩ LncRNAs also regulate chemosensitivity through crosstalk between apoptosis and autophagy. Beclin‐1 doesn't produce two fragments named “N” and “C.” Beclin‐1 is activated and can induce autophagy and inhibit apoptosis regulated by mitochondrial pathway.②⑥⑦⑨⑩ LncRNAs sponge miRNAs and then regulate downstream signaling pathways. (Note: The lncRNA‐miRNA complex is regarded as a whole, and the arrows emitted by the complex in the figure represent that the complex promotes the expression of microRNA target genes. B, LncRNAs regulate autophagy‐mediated chemosensitivity. The process of autophagy is divided into the following five different stages: ①Autophagy initiation; ②Nucleation; ③Elongation; ⑤Fusion with the lysosome; and ⑤Degradation. LncRNAs bind to miRNAs, or directly target autophagy protein Beclin‐1 or ATG family proteins or LC3 to regulate autophagy, thereby affecting chemosensitivity. C, LncRNAs regulate chemosensitivity by modulating MDR‐related genes. The ABC transporters not only excrete intracellular chemotherapy drugs, but also mediate lysosomal sequestration of chemotherapy drugs, then reducing the concentration of intracellular chemotherapy drugs, which makes lung cancer cells resistant to chemotherapy. LncRNAs bind to miRNAs or directly regulate MDR through modulating the expression of MDR‐related genes (MDR1, MRP1, and MRP7)

LncRNAs mainly regulate the chemosensitivity of lung cancer cells through the following mechanisms: 1) MAPK/ERK signaling pathway, 2) PI3K/AKT/mTOR and NF‐κB signaling pathway, 3) mitochondrial pathway, 4) STAT3 signaling pathway, 5) affecting cancer cell stemness by regulating the Wnt/β‐catenin signaling pathway, 6) activating or inactivating Beclin‐1 through crosstalk between apoptosis and autophagy, 7) epithelial to mesenchymal transition (EMT), 8) autophagy, and 9) multidrug resistance (MDR) (Figure 1).

Vimentin and E‐cadherin are EMT markers. Upregulated vimentin and downregulated E‐cadherin have been demonstrated to promote EMT, which contributes to chemoresistance.^61^ LncRNA NNT antisense RNA 1 (NNT‐AS1) and small nucleolar RNA host gene 12 (SNHG12) have been shown to upregulate the EMT‐related transcription factor Slug through the MAPK/ERK signaling pathway to promote EMT, making lung cancer cells resistant to chemotherapy.^62^, ^63^ Cancer stem cells (CSCs) are a type of cancer cell with the potential for self‐renewal and differentiation. Chemoresistance is a very significant feature of CSCs. The Wnt/β‐catenin signaling pathway has been reported to involve self‐renewal of CSCs and carcinogenesis.^64^ In cancer cells with CSC‐like characteristics, stemness‐related genes, such as *ATP binding cassette subfamily G member 2* (*ABCG2*), are activated.^65^ ABCG2 has been associated with clinical drug resistance through mechanisms involve drug efflux.^66^ Downregulation of tumor suppressor genes lncRNA maternally expressed 3 (MEG3) and AK126698 has been demonstrated to confer resistance to cisplatin in lung cancer cells by activating the Wnt/β‐catenin signaling pathway.^67^, ^68^ SOX 4 is a transcription factor in the Wnt/β‐catenin signaling pathway; Hu et al showed that lncRNA CCAT1 targets SOX4 by binding to miR‐130a‐3p, thereby upregulating ABCG2 and reducing the chemosensitivity of lung cancer cells.^69^ Zhao et al reported that lncRNA HOXA11 antisense RNA (HOXA11‐AS) bound to miR‐454‐3p and subsequently activated the STAT3 signaling pathway to mediate acquired resistance to cisplatin.^70^ Chen et al revealed that lncRNA HOX Transcript Antisense RNA (HOTAIR) reduces the chemosensitivity of lung cancer cells by activating the NF‐κB signaling pathway.^71^ Shi et alreported that lincRNA‐ROR (long intergenic non‐protein coding RNA, regulator of reprogramming) enhances cisplatin resistance by activating PI3K/Akt/mTOR signaling pathway and inhibiting mitochondrial pathway in lung cancer cells (Figure 1A).^72^

Autophagy is one of the degradation pathways of intracellular substances and has also been shown to be involved in enhancing the resistance of lung cancer cells to chemotherapy and promoting tumor progression (Figure 1B).^73^ Many autophagy‐related (ATG) proteins participate in the regulation of autophagy. When autophagy occurs, autophagy marker LC3 is cleaved to generate LC3‐I. LC3‐I is further processed to form LC3‐II, then transferred to the autophagosome membrane, and participates in the degradation process.^74^ Both lncRNA X‐inactive‐specific transcript (XIST) and bladder cancer‐associated transcript 1 (BLACAT1) can bind to miR‐17 and target autophagy‐related 7 (ATG7) to promote autophagy and reduce the chemosensitivity of lung cancer cells.^75^, ^76^ The tumor suppressor gene lncRNA growth arrest‐specific 5 (GAS5) and MEG3 have been found to inhibit autophagy by suppressing the cleavage of LC3, thereby enhancing the chemosensitivity of lung cancer cells.^77^, ^78^ However, the oncogene lncRNA plasmacytoma variant translocation 1 (PVT1), XIST, and MALAT1 have all been demonstrated to promote autophagy through LC3 cleavage, which can enhance the chemoresistance of lung cancer cells.^75^, ^79^, ^80^

The autophagy protein Beclin‐1 is involved in the crosstalk between apoptosis and autophagy. When the cells are continuously exposed to apoptosis stimulation, Beclin‐1 is cleaved into N‐ and C‐terminal fragments, thus losing the function of autophagy induction. The C‐terminal fragments are transferred to the mitochondria, thus triggering the mitochondrial pathway to induce apoptosis.^81^ Huang et al found that lncRNA BLACAT1 inhibits miR‐17 and upregulates Beclin‐1, promoting autophagy and enhancing cisplatin resistance in lung cancer cells.^76^ LncRNA PVT1 has been further confirmed to promote autophagy and inhibit the mitochondrial pathway by upregulating Bcl‐2 and downregulating Bax, thereby reducing chemosensitivity.^80^

MDR is one of the factors that affect the efficacy of chemotherapy. ATP‐binding cassette (ABC) transporters include multidrug resistance protein 1 (MDR1), multidrug resistance‐associated protein 1 (MRP1), multidrug resistance‐associated protein 7 (MRP7), and others. ABC transporters have been identified as multidrug efflux pumps which reduce the intracellular concentration of chemotherapeutic drugs by efflux mechanisms, conferring chemoresistance to lung cancer cells.^82^ ABC transporters can also mediate lysosomal activity to sequester hydrophobic weak‐base chemotherapeutic agents, then trigger the expansion of lysosomal compartments, and enhance drug sequestration. These molecular events prevent chemotherapeutic agents from reaching their targets located in the nucleus or cytoplasm.^83^

In lung cancer, lncRNAs regulate chemosensitivity by modulating MDR‐related genes (Figure 1C). Tian et al demonstrated that lncRNA XIST binds to miR‐144‐3p, then upregulates MDR1 and MRP1, making lung cancer cells resistant to cisplatin.^84^ Similarly, Fang et al showed that lncRNA MALAT1 confers cisplatin resistance to lung cancer via upregulation of MDR1 and MRP1.^85^ Ren et al reported that lncRNA KCNQ1 opposite strand/antisense transcript 1 (KCNQ1OT1) correlated to poor prognosis of patients with lung cancer and MDR. It has also been revealed that KCNQ1OT1 confers an acquired resistance to paclitaxel by increasing MDR1 expression.^86^ Xu et al found that FOXF1 adjacent noncoding developmental regulatory RNA (FENDRR) is correlated with acquisition of sensitivity to cisplatin by downregulating MRP7 in lung cancer cells.^87^

#### 7.1.2 Radiosensitivity

For patients with NSCLC, the 5‐year relative survival rate is generally rather low, because 60%–80% of patients with NSCLC are not diagnosed until the cancer is already at an advanced stage.^88^ For unresectable NSCLC, the combination of chemotherapy and radiotherapy can suppress distant metastasis of the tumor and improve the survival rate of patients.^89^ Similar to chemotherapy, however, the effectiveness of radiotherapy is limited by the radioresistance of lung cancer cells. Table S3 summarizes some lncRNAs purported as predictive markers of radiosensitivity in lung cancer.

##### Signaling pathways related to radiosensitivity

**FIGURE 2:**
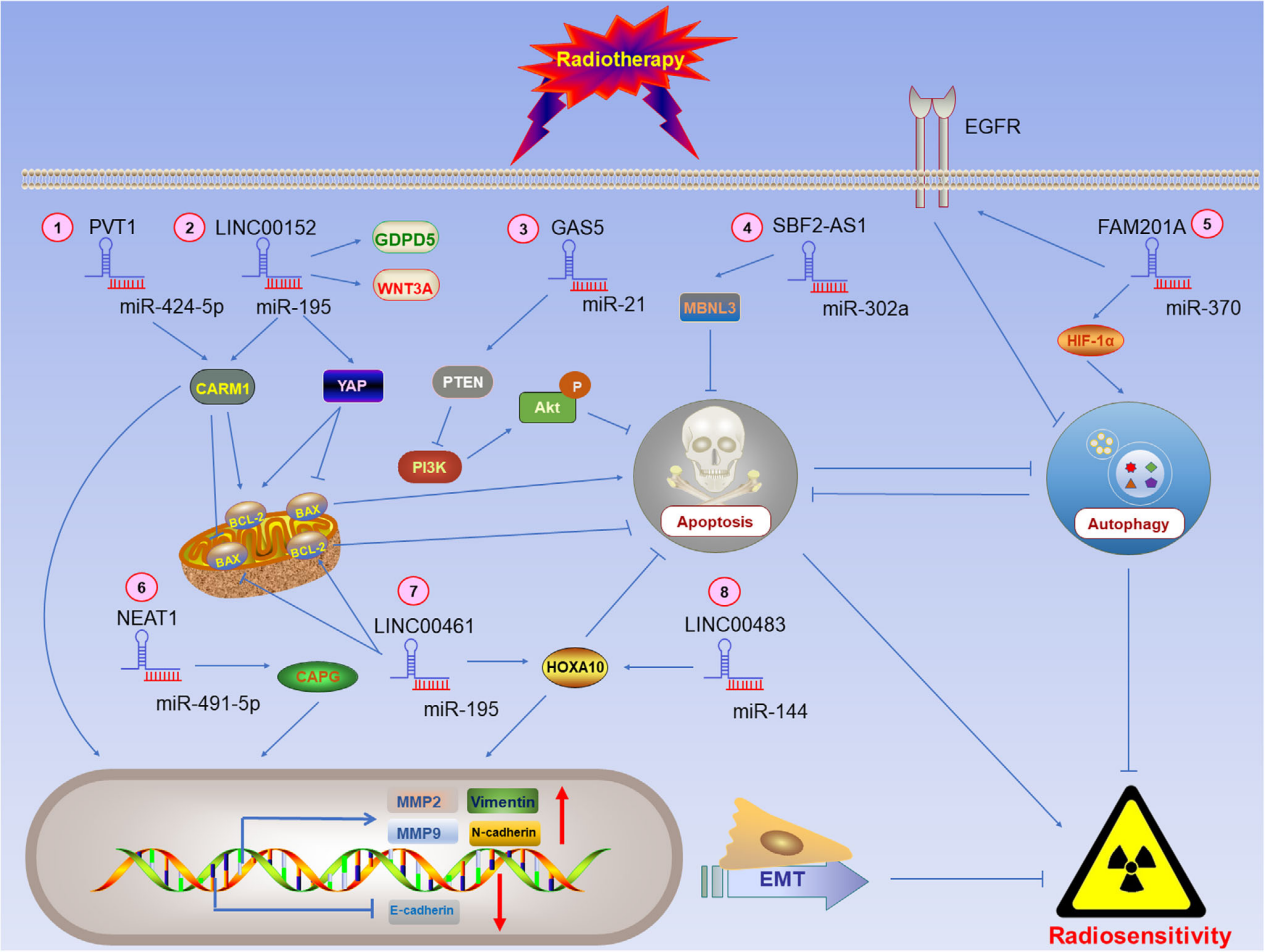
LncRNAs sponge microRNAs and then regulate radiosensitivity through different signaling pathways. P: phosphorylated. ①②③④⑦ LncRNAs regulate apoptosis‐mediated radiosensitivity. Specifically, ①②③⑦ lncRNAs affect radiosensitivity by regulating the intrinsic pathway of apoptosis, Hippo, and PI3K/AKT signaling pathways. ⑤ LncRNAs regulate autophagy‐mediated radiosensitivity through HIF‐1α signaling pathway. ①⑥⑦⑧ lncRNAs also regulate EMT‐mediated radiosensitivity via targeting EMT markers and matrix metalloproteinases (MMPs). (Note: The lncRNA‐miRNA complex is regarded as a whole, and the arrows emitted by the complex in the figure represent that the complex promotes the expression of miRNA target genes)

Three main mechanisms affect the radiosensitivity of lung cancer cells (Figure 2). The first of these is apoptosis. Many cancer treatments (including radiotherapy) kill tumor cells by promoting apoptosis. Apoptotic pathways include the extrinsic pathway of apoptosis mediated by death receptors, as well as an intrinsic pathway of mitochondrial‐mediated apoptosis (mitochondrial pathway).^90^ Anti‐apoptotic protein Bcl‐2 localized on the outer membrane of mitochondria inhibits the translocation of pro‐apoptotic protein Bax from cytoplasm to mitochondria, suppressing activation of caspase‐9 and caspase‐3, thereby inhibiting the intrinsic pathway of mitochondrial‐mediated apoptosis.^91^, ^92^ The second mechanism of radiosensitivity is autophagy; note that there is also a notable crosstalk involved between autophagy and apoptosis. Activity of Bcl‐2 inhibits autophagy.^93^ Autophagy has been shown to be upregulated in cancer cells with radioresistance and is capable of antagonizing apoptosis.^94^ The degree of hypoxia in tumor tissue is one of the factors that can affect the efficacy of radiotherapy. Under hypoxic conditions, autophagy is increased through the HIF‐1α signaling pathway, which is beneficial to the survival of tumor cells.^95^ The third mechanism is EMT. Upregulated matrix metallopeptidase 2 (MMP‐2) and matrix metallopeptidase 9 (MMP‐9) promote migration of lung cancer cells.^96^ Vimentin, N‐cadherin, and E‐cadherin are EMT markers; loss of epithelial phenotypic markers such as E‐cadherin and the upregulation of the mesenchymal phenotypic marker (eg. Vimentin, N‐cadherin) can promote EMT, which contributes to radioresistance.^97^ EMT is associated with a poor prognosis in lung cancer.^98^

Chen et al reported that lncRNA GAS5, a tumor suppressor, increases radiosensitivity by inhibiting miR‐21 and activating PTEN/PI3K/Akt signaling pathway.^99^ Zhang et al demonstrated that cytoskeleton regulator RNA (CYTOR, LINC00152) serves as a ceRNA to sponge miR‐195 and regulates the expression of downstream Yes‐associated protein (YAP) to affect radiosensitivity of lung cancer.^100^ YAP is an important regulatory protein related to the Hippo signaling pathway. Activation of the Hippo signaling pathway has been confirmed to inhibit apoptosis through downregulating Bax and upregulating Bcl‐2.^101^ Yang et al showed that LINC00483 binds to miR‐144, upregulates homeobox A10 (HOXA10), and subsequently upregulates MMP‐2 and MMP‐9 and to modulate expression of EMT markers (e.g., Vimentin, N‐cadherin, and E‐cadherin); this was associated with promotion of migration of lung cancer cells and a reduction in radiosensitivity.^102^ Likewise, Hou et al revealed that LINC00461 can act as a ceRNA interacting with miR‐195 to inhibit the mitochondrial pathway. This also upregulates HOXA10, MMP‐2, and MMP‐9, contributing to migration of lung cancer cells and ultimately reducing radiosensitivity.^103^ Liu et al found that family with sequence similarity 201 member A (FAM201A), which is highly expressed in lung cancer, binds to miR‐370, and downregulates epidermal growth factor receptor (EGFR) and hypoxia‐inducible factor 1alpha (HIF‐1α) to reduce radiosensitivity.^104^ In tumor progression, EGFR negatively regulates autophagy.^105^ LncRNA PVT1, SBF2‐AS1, and nuclear paraspeckle assembly transcript 1 (NEAT1) are also involved in this regulatory network of lncRNA‐microRNA‐mRNA and can affect the radiosensitivity of lung cancer cells.^106^, ^107^, ^108^, ^109^ Wang et al reported that lncRNA PVT1 competitively binds miR‐424‐5p, upregulating the target gene coactivator‐associated arginine methyltransferase 1 (CARM1), then upregulates Bcl‐2 while downregulating Bax to inhibit mitochondrial pathway. *PVT1* also increased the expression of MMP‐2 and MMP‐9 to promote EMT, which ultimately made lung cancer cells radioresistant.^107^ Yu et al confirmed that lncRNA SBF2‐AS1 reduced radiosensitivity by regulating a miR‐302a/MBNL3 (muscleblind‐like 3) axis in lung cancer.^108^ Similarly, Zhou et al showed that lncRNA NEAT regulated miR‐491‐5p/CAPG (Capping Actin Protein, Gelsolin Like) axis to promote EMT and lung cancer invasion, enhancing radioresistance of tumors.^109^

#### 7.1.3 Sensitivity to EGFR‐targeted therapy

EGFR possesses tyrosine kinase activity can bind to epidermal growth factor (EGF) to regulate cell growth, proliferation, differentiation, migration, and other physiological functions. Statistical analysis of data from large cohorts has shown that there are high levels of EGFR expression in tissues of at least 10 cancer types, including lung cancers,^110^ in comparison with normal tissues. From 40% to 80% of NSCLC patients are found to harbor tumors exhibiting dysregulation of EGFR in some cohorts. Mutations of EGFR are identified as one of the factors that can induce NSCLC and have often been found to occur in exons 19 and 21.^111^ For lung cancer patients with EGFR mutations, targeted therapy based on EGFR tyrosine kinase inhibitors (EGFR‐TKIs) is widely used in clinical treatment.^112^ However, many NSCLC patients develop resistance to EGFR‐TKIs (e.g., gefitinib, erlotinib, afatinib, and osimertinib) after 9–14 months of treatment. The T790M mutation of EGFR often induces lung cancer cells to acquire resistance to EGFR‐TKIs.^113^ A massive number of lncRNAs have been identified to regulate the sensitivity of lung cancer cells to EGFR‐TKIs. Table S4 shows several lncRNAs that have been claimed to be predictive markers of sensitivity to EGFR‐targeted therapy in lung cancer.

##### Signaling pathways related to sensitivity to EGFR‐targeted therapy

Gefitinib and erlotinib are EGFR‐TKIs commonly used in the treatment of advanced NSCLC. Results have demonstrated, though, that as the treatment time increases, lung cancer tumors commonly develop resistance to EGFR‐TKIs.^114^ Associated with PI3K/Akt and Ras/Raf/MAPK, which downstream signaling pathways for EGFR, P‐MAPK and P‐Akt have been reported to evaluate the efficacy of gefitinib in patients with advanced NSCLC.^115^

**FIGURE 3:**
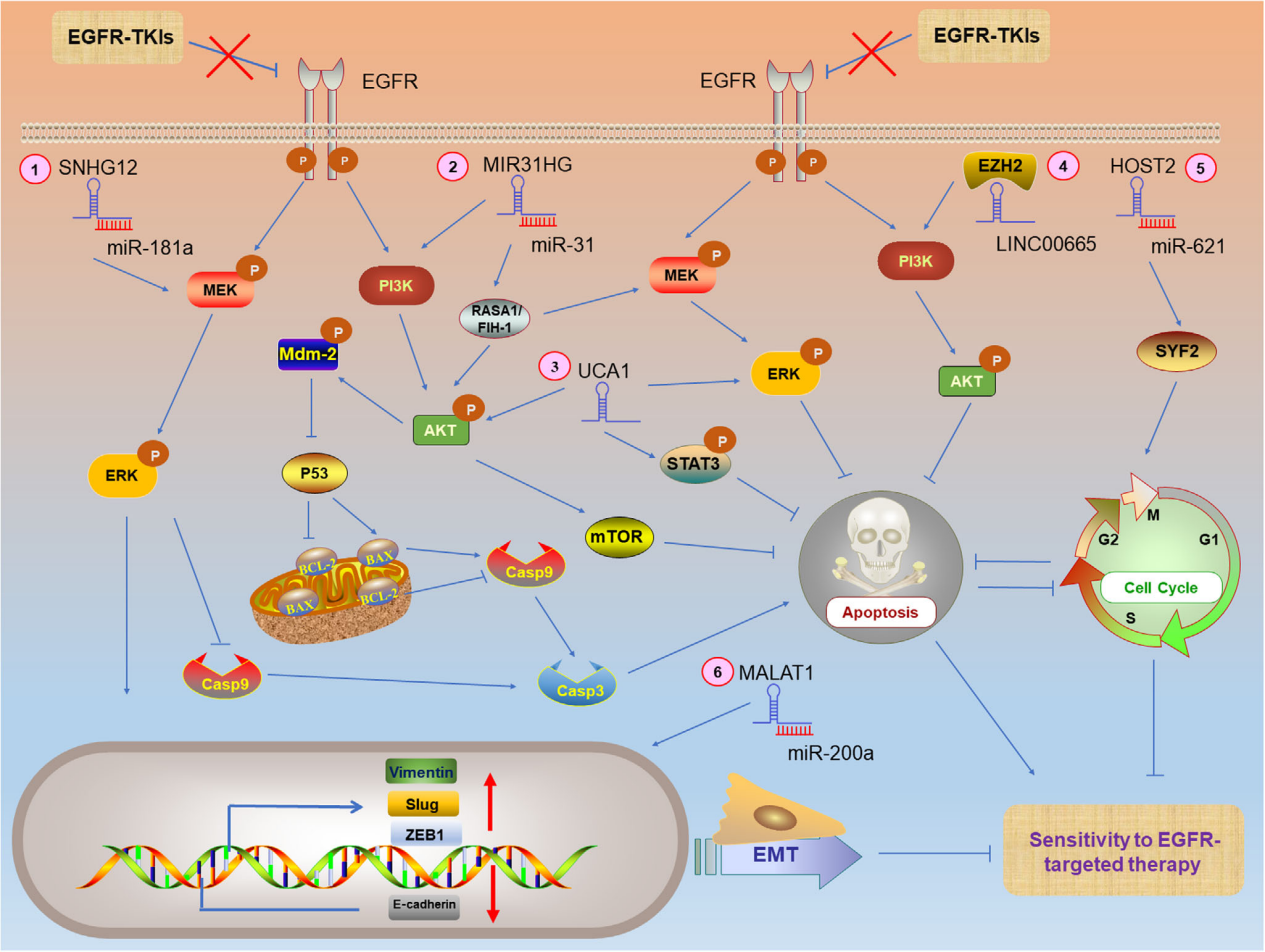
LncRNAs regulate sensitivity to EGFR‐targeted therapy through different signaling pathways in EGFR‐TKIs‐resistant lung cancer cells. P: phosphorylated. ①②③④ LncRNAs regulate sensitivity to EGFR‐targeted therapy by modulating MAPK/ERK, PI3K/AKT/mTOR, STAT3 signaling pathways, and mitochondrial pathway.②⑤ LncRNAs regulate cell cycle‐dependent sensitivity to EGFR‐targeted therapy. ①③⑥ lncRNAs also regulate EMT‐mediated sensitivity to EGFR‐targeted therapy via targeting EMT markers or transcription factors. (Note: The complex (e.g., lncRNA‐miRNA, lncRNA‐protein) is regarded as a whole, and the arrows emitted by the complex in the figure represent that the complex promotes the expression of target genes)

LncRNAs regulate the sensitivity of lung cancer cells to EGFR‐TKIs chiefly through one of the following mechanisms: 1) MAPK/ERK signaling pathway, 2) PI3K/AKT/mTOR signaling pathway, 3) STAT3 signaling pathway, 4) mitochondrial pathway, 5) cell cycle progression, or 6) EMT (Figure 3).

In NSCLC, upregulated vimentin, EMT‐related transcription factors (e.g., ZEB1, Slug), and downregulated E‐cadherin are associated with promotion of EMT, thereby increasing lung cancer resistance to EGFR‐TKIs.^116^, ^117^, ^118^

Wang et al revealed that lncRNA SNHG12 interacts with miR‐181a, activating the MAPK pathway, upregulating Slug to promote EMT, and suppressing the activation of caspase‐3,9 to inhibit the mitochondrial pathway. The upregulated SNHG12 confers resistance to gefitinib in lung cancer cells.^63^ MIR31HG has been found to activate MAPK/ERK and PI3K/AKT signaling pathways to upregulate Mdm‐2, reduce activity of tumor suppressor gene *P53*, and thereby it can inhibit the mitochondrial pathway and promote cell cycle progression. MIR31HG thus contributes to a reduction in the sensitivity of lung cancer cells to gefitinib.^119^, ^120^ LncRNA UCA1 has been demonstrated to promote EMT and mediates acquired resistance to gefitinib in lung cancer cells through MAPK/ERK, AKT/mTOR, and STAT3 signaling pathways.^121^, ^122^ Liu et al found that LINC00665 interacted with Enhancer of Zeste homolog 2 (EZH2) and activated the PI3K/AKT signaling pathway to reduce the sensitivity of lung cancer cells to gefitinib.^123^ Feng et al reported that LncRNA MALAT1 promoted EMT‐mediated gefitinib resistance via miR‐200a/ZEB1 axis.^124^ Chen et al showed that lncRNA human ovarian cancer‐specific transcript 2 (HOST2) acted as a ceRNA to inhibit miR‐621 and upregulated cell cycle‐associated protein SYF2, thereby promoting cell cycle progression and a consequential reduction in the sensitivity of lung cancer cells to gefitinib.^125^

### 7.2 LncRNAs as diagnostic markers in lung cancer

Early detection and intervention in clinical cases of lung cancer may prevent tumor progression. In the early screening of lung cancer, LDCT is often used for diagnostic imaging tests. However, because of its high false positive rate, it remains suboptimal; radiologists can sometimes confuse cancerous nodules with benign NCN. This may subject misdiagnosed patients to subsequent unnecessary invasive examinations.^126^ Detection utilizing blood‐based biomarkers has the advantage of being a noninvasive approach and can be used as an auxiliary method of LDCT for early screening of lung cancer. The molecular markers routinely used for classification of different histological subtypes differ. Neuron‐specific enolase (NSE) and progastrin‐releasing peptide (ProGRP) are often used as tumor markers for SCLC. Carcinoembryonic antigen (CEA), squamous cancer cell antigen (SCCA), and cytokeratin 19‐fragments (CYFRA 21‐1) are routinely used as tumor markers for NSCLC.^127^ However, histological analysis is inherently objective and lacks high specificity. For example, some tumor markers, such as CEA, are not only increased in lung cancer, but can also be elevated in some benign tumors or non‐carcinogenic diseases.^128^ Therefore it is very urgent to identify novel, more specific and more sensitive biomarkers to diagnose early stage lung cancer. Many studies have reported that lncRNAs can serve as novel and promising molecular markers for lung cancer diagnosis (Table S5). Similar to other blood‐based tumor markers, some lncRNAs are not very sensitive in the diagnosis of lung cancer, such as MALAT1. This is because that MALAT1 is abnormally expressed in many different types of cancer.^129^ A research by Weber et al revealed that the sensitivity of MALAT1 in NSCLC screening is only 56%. For the glandular subtype LAD of NSCLC, the sensitivity of MALAT1 is even lower, only 48%. Obviously, MALAT1 cannot be used as a single molecular marker to diagnose early stage lung cancer.^130^ The limitation of a blood‐based molecular marker that does not have a relatively high sensitivity can be mitigated by resorting to the selection of multiple molecular markers measured simultaneously in a panel. As part of a panel, molecular markers can be used in complementary fashion, resulting in improved diagnostic performance.^131^ The results of Xie et al confirmed such use of multiple molecular markers. LncRNA SOX2OT, antisense noncoding RNA in the INK4 locus (ANRIL), CEA, SCCA, and CYFRA21‐1 were selected to form a diagnostic panel for NSCLC. Compared with any single biomarker, higher specificity (79.2%) and sensitivity (77.1%) of the panel were calculated using a logistic regression model. In an independent validation set containing clinical data, the specificity (70.0%) and sensitivity (91.0%) of this panel were also significantly higher than the diagnostic performance of single molecular markers.^132^

**FIGURE 4:**
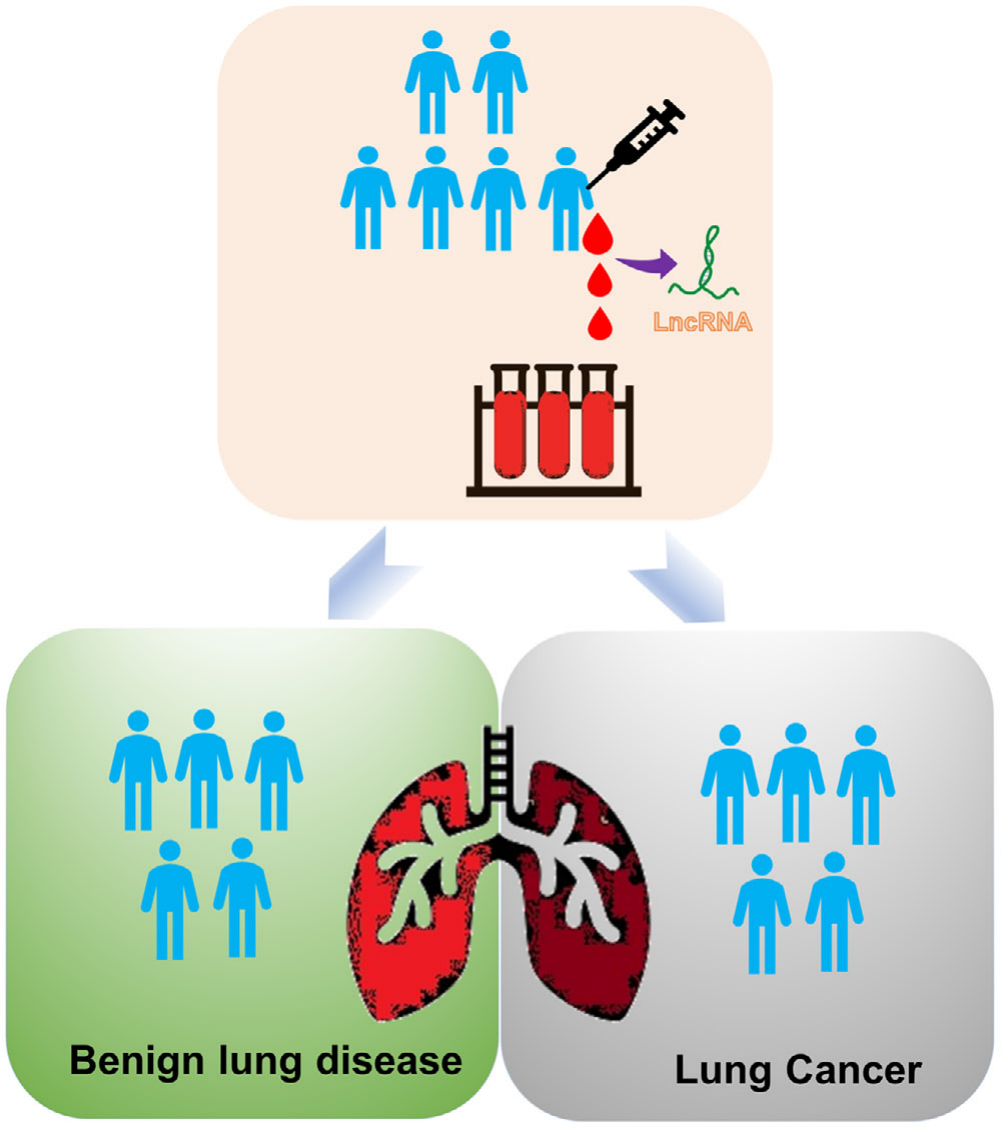
LncRNAs are used as a diagnostic tool for early lung cancer screening. Because LDCT has a high false positive rate, lncRNAs can be act as an auxiliary detection tool to diagnose diseases. LncRNAs are stable in the blood. After LDCT screening, patients were drawn blood to extract RNAs. Identifying the expression levels of different lncRNAs in the patient's blood by PCR, which can distinguish between lung cancer and benign lung disease

In sufficient quantity, tumor‐derived lncRNAs typically form a highly stable secondary structure, which is resistant to ribonuclease activity and is thus stable in peripheral blood, making lncRNAs suitable for quantitative detection. The lncRNAs present in blood serve as emerging tumor‐specific molecular markers and are used for early cancer detection.^133^, ^134^ In statistics, the receiver operating characteristic curve reflects the relationship between sensitivity and specificity in a diagnostic test. In a test of the utility of lncRNAs as a diagnostic marker, the area under the curve (AUC) can be used to quantify diagnostic accuracy. The value of AUC is a percentage which ranges between 0 and unity. Higher values of AUC indicate a higher diagnostic accuracy of the test.^135^ In clinical diagnosis, lung cancer is often confused with benign diseases of the lung (e.g., pneumonia), which often poses a dilemma in the subsequent treatment of patients. Some lncRNAs have been identified that can effectively serve to establish a differential diagnosis between lung cancer and benign lung disease (Figure 4).^136^ Recently, Linc00152 has been shown to hold potential to serve as a diagnostic marker distinguishing NSCLC from benign lung disease. In a cohort where this type of assay was employed, the value of AUC was calculated to be 0.742.^137^ Similarly, Jiang et al found that lncRNA XLOC_009167 could be reliably detected with high expression in the whole blood of lung cancer patients. The value of AUC reported by this group was 0.7005, indicating that this diagnostic test has modestly high accuracy. Consistent with the high sensitivity of molecular assays, the sensitivity of lncRNA XLOC_009167 to distinguish between lung cancer and pneumonia was reported to be as high as 90.1%.^133^ In addition to the possibility of sparing some ill patients from an invasive biopsy when no malignancy is present, clinical testing of this sort is also facilitated since there is no need to separate serum or plasma from whole blood.

Not only in lung cancer, some studies have confirmed that lncRNAs also have diagnostic potential in other types of cancer. For example, long intergenic non‐protein coding RNA 2418 (LINC02418) can be regarded as biomarkers to diagnose colorectal cancer (CRC).^138^ Similarly, according to the results of Gong et al, MIR4435‐2 host genes (MIR4435‐2HG) were identified to serve as a diagnostic marker for ovarian cancer.^139^ In addition, Liu et al showed that ABHD11 antisense RNA 1 (ABHD11‐AS1) had potential to act as a diagnostic marker distinguishing early‐stage pancreatic cancer from benign pancreatic disease and healthy controls.^140^

### 7.3 LncRNAs as prognosis markers in lung cancer

Prognosis is closely associated with the choice of treatment for lung cancer patients and the improvement of survival rate. In NSCLC, the main subtype of lung cancer, the 5‐year relative survival rate of patients is extremely low. Clinical cases are often complicated by lymph node metastasis and distant metastasis of tumor, which are related to poor prognosis.^141^ Although the tumor node metastasis (TNM) staging system has been established as a very valuable method for predicting the prognosis of NSCLC, it is not able to accurately assess prognosis.^142^ Univariate and multivariate analysis are standard and reliable statistical methods used to prove whether an lncRNA can be regarded as an independent tumor marker for predicting prognosis of lung cancer patients.^143^ Some lncRNAs have also been identified as independent prognostic markers in lung cancer (Table S6). It is suggestible, therefore, that serial measurements of prognosis‐associated lncRNAs might gauge individual response to therapy.

## 8 DISCUSSION

### 8.1 Future prospect

#### 8.1.1 Immune‐related lncRNAs as therapeutic targets for immunotherapy

At present, research involving lncRNAs mainly focuses on biological processes such as epigenetic regulation, cancer development and progression, and cell differentiation.^144^, ^145^, ^146^ However, lncRNAs can also act as regulators of gene expression in the immune system.^147^ The immune system has the dual function of promoting or suppressing tumors, depending on the three stages of cancer immunoediting: elimination, equilibrium, and escape.^148^ During the processes of oncogenic transformation and tumor propagation, abnormal immune function is an important characteristic that often occurs.^149^ LncRNAs have been shown to be involved in different stages of the cancer‐immunity cycle: 1) lncRNAs regulate the release of antigens, 2) lncRNAs induce the maturation of antigen‐presenting cells by regulating a series of pro‐inflammatory cytokines, 3) differentiation of immune cells is regulated by lncRNAs upon immune activation, 4) lncRNAs can influence the migration of immune cells, 5) lncRNAs affect the infiltration of T cells into cancer tissues, and 6) lncRNAs regulate processes by which the immune system recognizes and kills cancer cells.

Work by Li et al identified the regulation of lncRNAs in the immune system of 33 cancers and the potential of these lncRNAs as immunotherapeutic targets through an integrated algorithm, ImmLnc.^150^ In 33 types of cancer, on average, the research by the novel bioinformatics technique has suggested that there may be as many as 2000 lncRNAs of each cancer type related to immune pathways. Most of these lncRNAs were found to be related to “cytokine” and “cytokine receptors” pathways. Cytokine administration is one of the methods of immunotherapy.^151^ Also, the closely‐related chemokine system is an emerging potential target for immunotherapy. Xing et al found that lncRNA induced by chemokines participates in the regulation of cancer cell metastasis.^152^ Much contemporary research has studied the biology of cancer cells regulated by lncRNAs, while detailed mechanism of lncRNAs in the body's immune response to cancer is a relatively neglected topic. Therefore, the immune‐related lncRNAs may become potential therapeutic targets for subsequent exploration of lung cancer immunotherapy.

#### 8.1.2 m^6^A RNA methylation

By the close of 2017, 163 RNA modifications had been identified in MODOMICS.^153^ N^6^‐methyladenosine (m^6^A) methylation is the most common RNA modification in eukaryotic mRNA.^154^ m^6^A methylation has been confirmed to be a dynamic and reversible process^155^ and is involved in RNA transcription, post‐transcriptional processing, regulation of mRNA stability, translation and other biological events.^154^

m^6^A methylation is associated with the occurrence and development of lung cancer. Li et al showed that m^6^A demethylase FTO promotes the proliferation and growth of lung cancer cells by stabilizing ubiquitin‐specific peptidase 7 (USP7) mRNA.^156^ Likewise, an m6A methyltransferase, methyltransferase‐like 3 (METTL3) has been reported to promote transforming growth factor‐β‐dependent EMT in lung cancer by regulating the transcription factor JUNB.^157^ Moreover, lncRNAs modified by m^6^A methylation can affect the expression of downstream target genes through specific signaling pathways.^158^ Yang et al revealed that another m^6^A methyltransferase, methyltransferase‐like 14 (METTL14) inhibits the growth and metastasis of CRC by promoting degradation of XIST.^159^ At present, the regulatory mechanism of m^6^A methylation of lncRNAs for the occurrence and development of lung cancer is not clear. Therefore, lung cancer treatment strategies surrounding the m^6^A methylation of lncRNAs need to be explored in depth.

## 9 CONCLUSION

Since lncRNAs do not have the function of protein coding, they were once considered as transcriptional noise.^160^ As more new lncRNAs being identified, the functions of lncRNAs have likewise been recognized as increasingly diverse as well.

**FIGURE 5:**
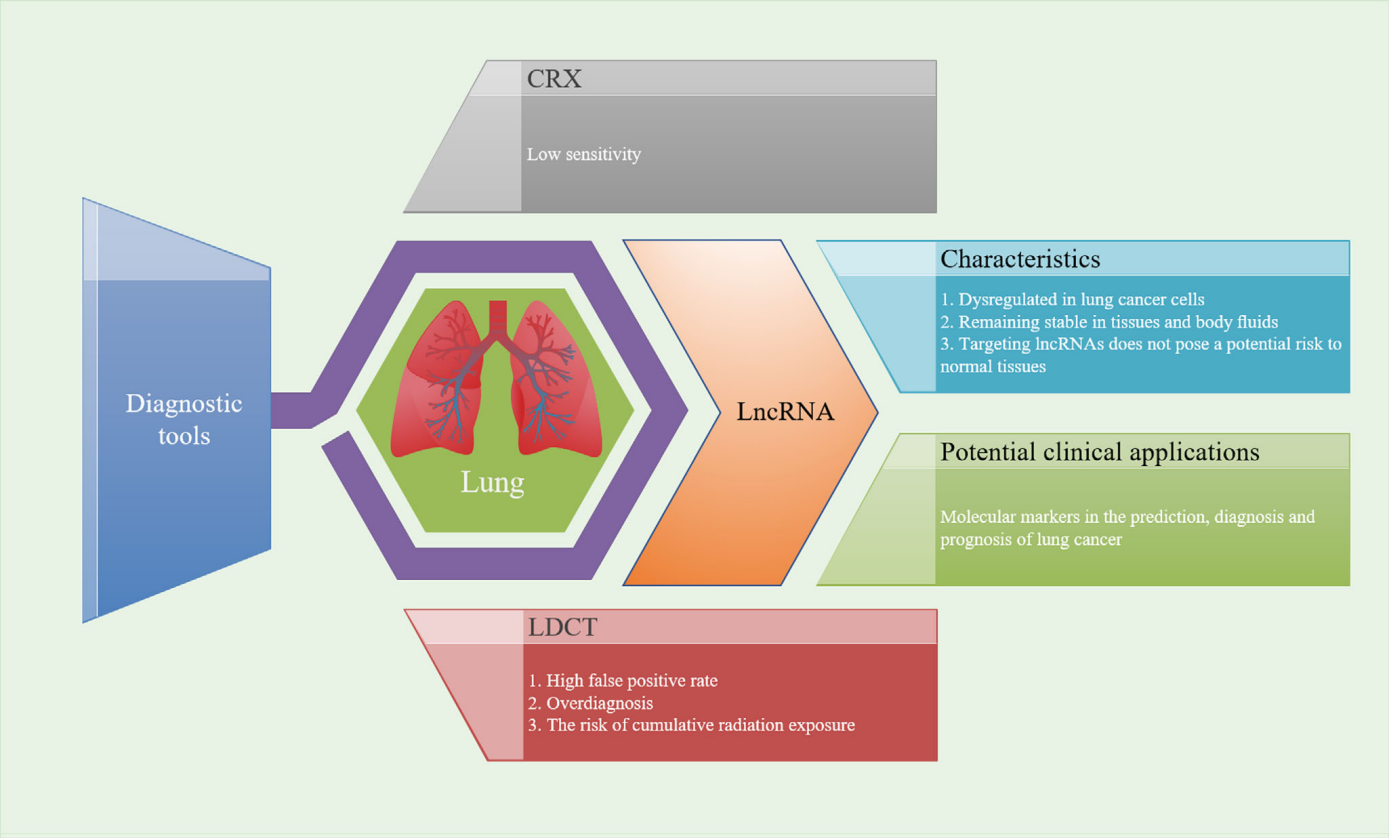
The disadvantages of CRX and LDCT and the characteristics and advantages of lncRNAs in the clinical applications of lung cancer

In clinical studies of lung cancer, lncRNAs have three noteworthy characteristics (Figure 5). First, a series of lncRNAs are dysregulated in lung cancer cells. Second, lncRNAs can stably persist in tissues and body fluids. Third, targeting lncRNAs does not pose a potential risk to normal tissues. Therefore, the development of personalized lung cancer treatment strategies around aberrantly‐expressed lncRNAs has potential clinical significance.^161^

**FIGURE 6:**
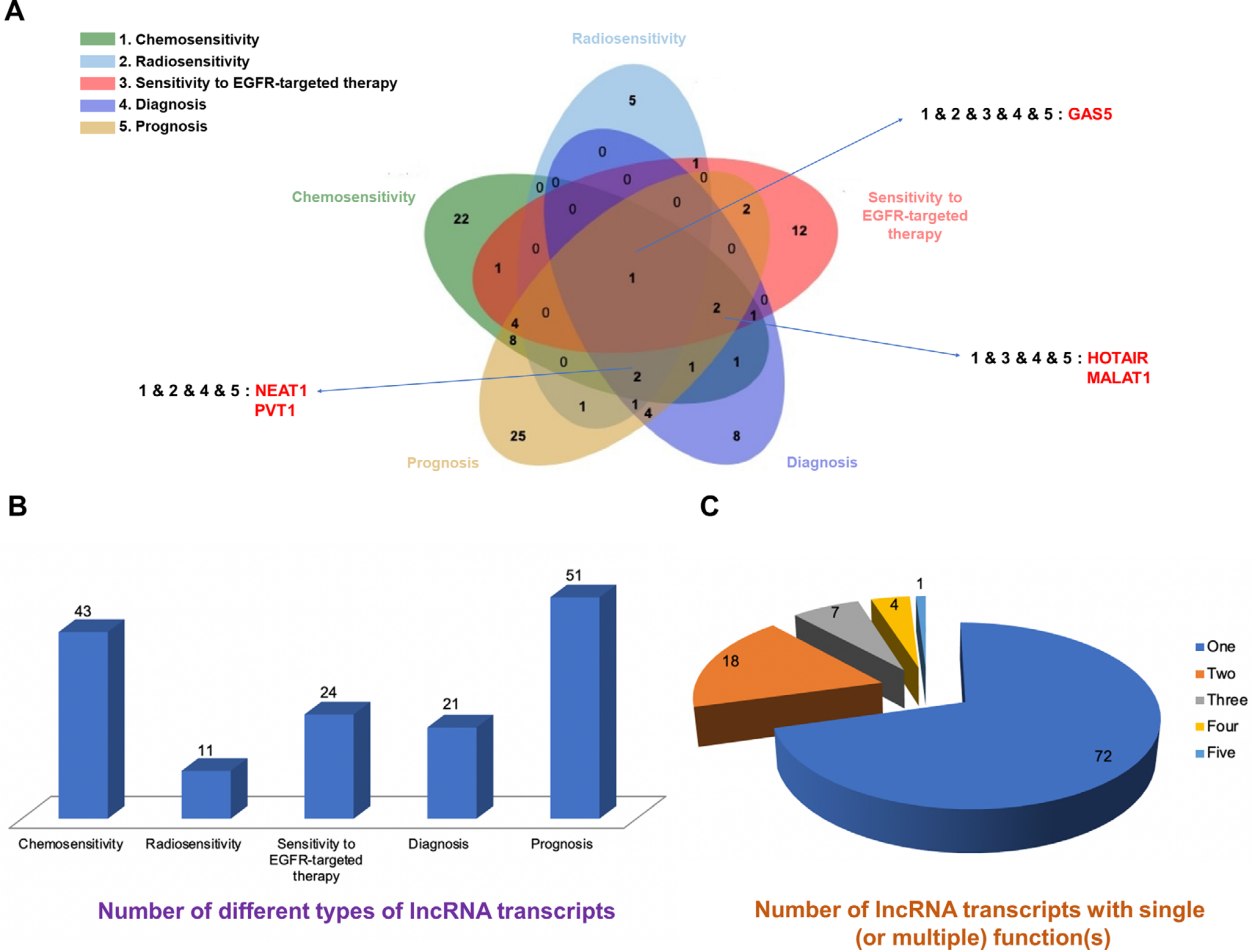
The lncRNAs from Tables S2, S3, S4, S5, and S6 were selected and divided into five types according to their different uses as markers: chemosensitivity, radiosensitivity, sensitivity to EGFR‐targeted therapy, diagnosis, and prognosis. They were sequentially labeled with serial numbers 1–5. A, The Venn diagram is made. Among them, the lncRNA with five types of functions is GAS5. The lncRNA with functions of 1 and 3 and 4 and 5 are HOTAIR and MALAT1. The lncRNAs with functions of 1 and 2 and 4 and 5 are NEAT1 and PVT1.Other overlapping or non‐overlapping areas show no details. B, Among the five types, the histogram shows the number of different types of lncRNA transcripts. C, According to the overlapping and non‐overlapping parts of Venn diagram (A), the number of lncRNA transcripts with a single function or multiple functions is shown

Due to the high false positives rate of LDCT, the potential risk of overdiagnosis and the cumulative hazards of radiation exposure, assay of lncRNA as a novel type of molecular marker can be used not only to assist LDCT to improve the accuracy of early lung cancer screening, but also can potentially aid in evaluation of drug resistance and prognosis of lung cancer patients throughout treatment. Some lncRNAs have been shown to serve as functional molecular markers in lung cancer in at least two capacities (Figure 6). For example, lncRNA NEAT1 and PVT1 not only reflect the sensitivity of lung cancer patients to chemotherapy and radiotherapy, but also serve as molecular markers for diagnosis and prognosis (Tables S2, S3, S5, and S6).^80^, ^106^, ^107^, ^109^, ^162^, ^163^, ^164^, ^165^, ^166^ In conjunction with statistical techniques, a carefully selected set of lncRNAs can be selected to form a panel which may improve the accuracy of diagnosis and prognosis in clinical situations. For instance, the expression level of lncRNA SOX2OT is closely related to histological subtype. Compared with LAD patients, the expression level of lncRNA SOX2OT in LUSC patients was significantly increased. Therefore, lncRNA SOX2OT might be an important indicator to distinguish LUSC from LAD. Differentiating NSCLC patients from healthy controls, the panel formed by the combination of GAS5 and SOX2OT has higher specificity (81.4%) and sensitivity (83.8%) than either one alone. Utilizing this panel, the expression levels of lncRNA GAS5 and SOX2OT in NSCLC patients can be detected and may distinguish between different TNM stages. Both lncRNA GAS5 and SOX2OT can be used as independent prognostic factors.^167^

Tumor hypoxia is one of the characteristics of malignant tumors and is a poor prognostic indicator. It has been associated with radioresistance and chemoresistance. Inhibiting the hypoxia of tumors has had a positive influence during treatment of lung cancer patients.^168^

**FIGURE 7:**
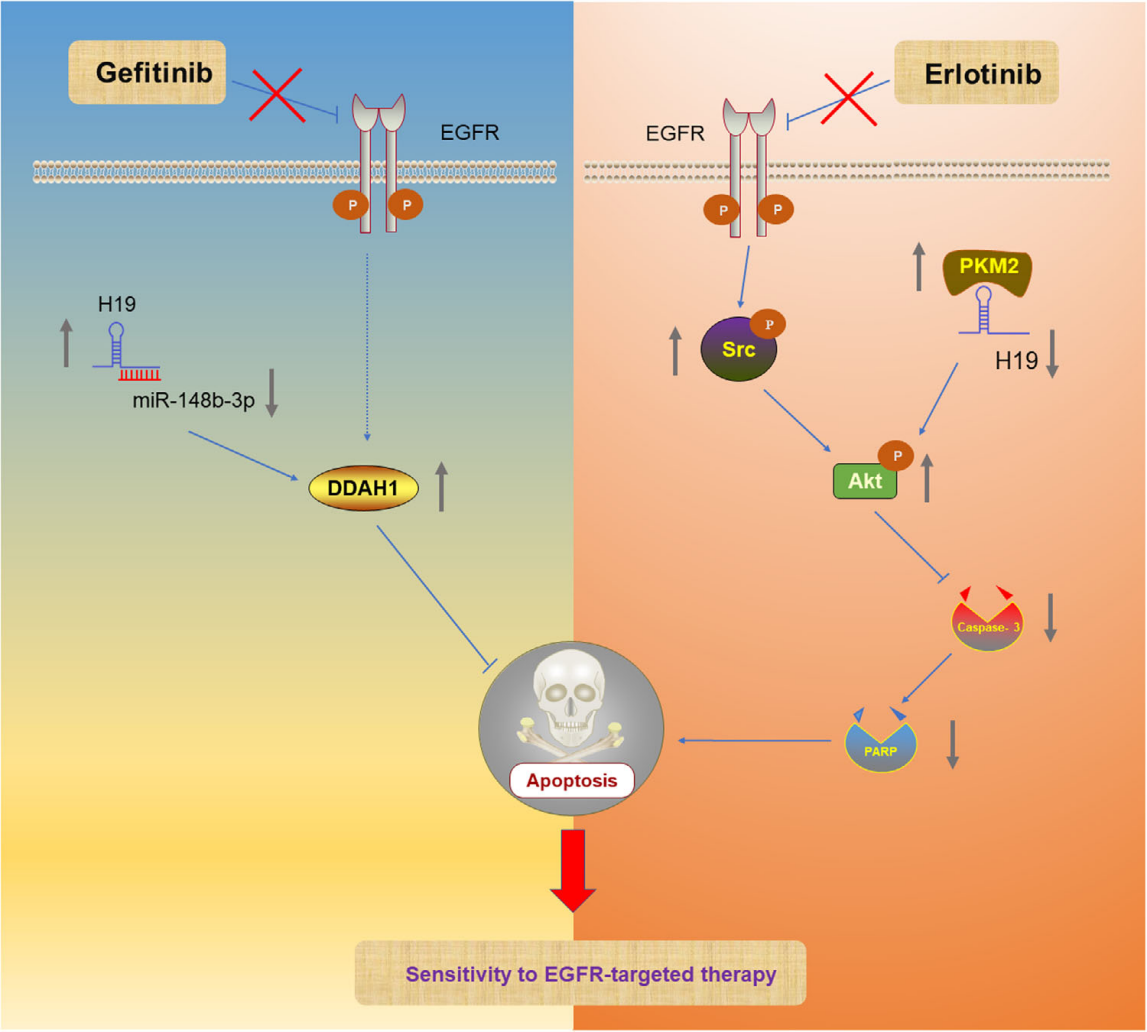
LncRNA H19 regulates sensitivity to EGFR‐targeted therapy through different mechanisms. P: phosphorylated. In geftinib‐resistant lung cancer cells, H19 affects sensitivity to EGFR‐targeted therapy by modulating miR‐148b‐3p/DDAH1 axis. In erlotinib‐resistant lung cancer cells, H19 affects sensitivity to EGFR‐targeted therapy through regulating the Src‐dependent Akt signaling pathway. (Note: The lncRNA‐protein complex is regarded as a whole, and the arrows emitted by the complex in the figure represent that the complex promotes the expression of downstream target genes. The gray arrows represent up‐ or downregulation of lncRNAs or proteins)

LncRNA H19 is involved in the development of almost all human cancers, including lung cancer. However, H19 plays different roles in the different stages of tumor initiation and progression, so it is still controversial as to whether H19 is truly an oncogene or a tumor suppressor gene.^169^ hnRNPA2B1‐dependent H19 has been reported to be contained in exosomes, with a capacity to transfer gefitinib resistance to tumor cells.^170^ Huang et al demonstrated that lncRNA H19 was upregulated in geftinib‐resistant LAD cells and regulated sensitivity to geftinib by a mechanism involving a miR‐148b‐3p/DDAH1 axis.^171^ However, lncRNA H19 has been found to be downregulated in erlotinib‐resistant EGFR mutant lung cancer cells and can regulate resistance to erlotinib by interaction with pyruvate kinase M2 (PKM2) and activation of a Src‐dependent Akt signaling pathway (Figure 7).^172^, ^173^ Therefore, more experiments in vivo and in vitro, as well as more clinical data, are needed to explore the exact role played by H19 regulating sensitivity to the various EGFR‐TKIs used in clinical treatment of lung cancer.

**FIGURE 8:**
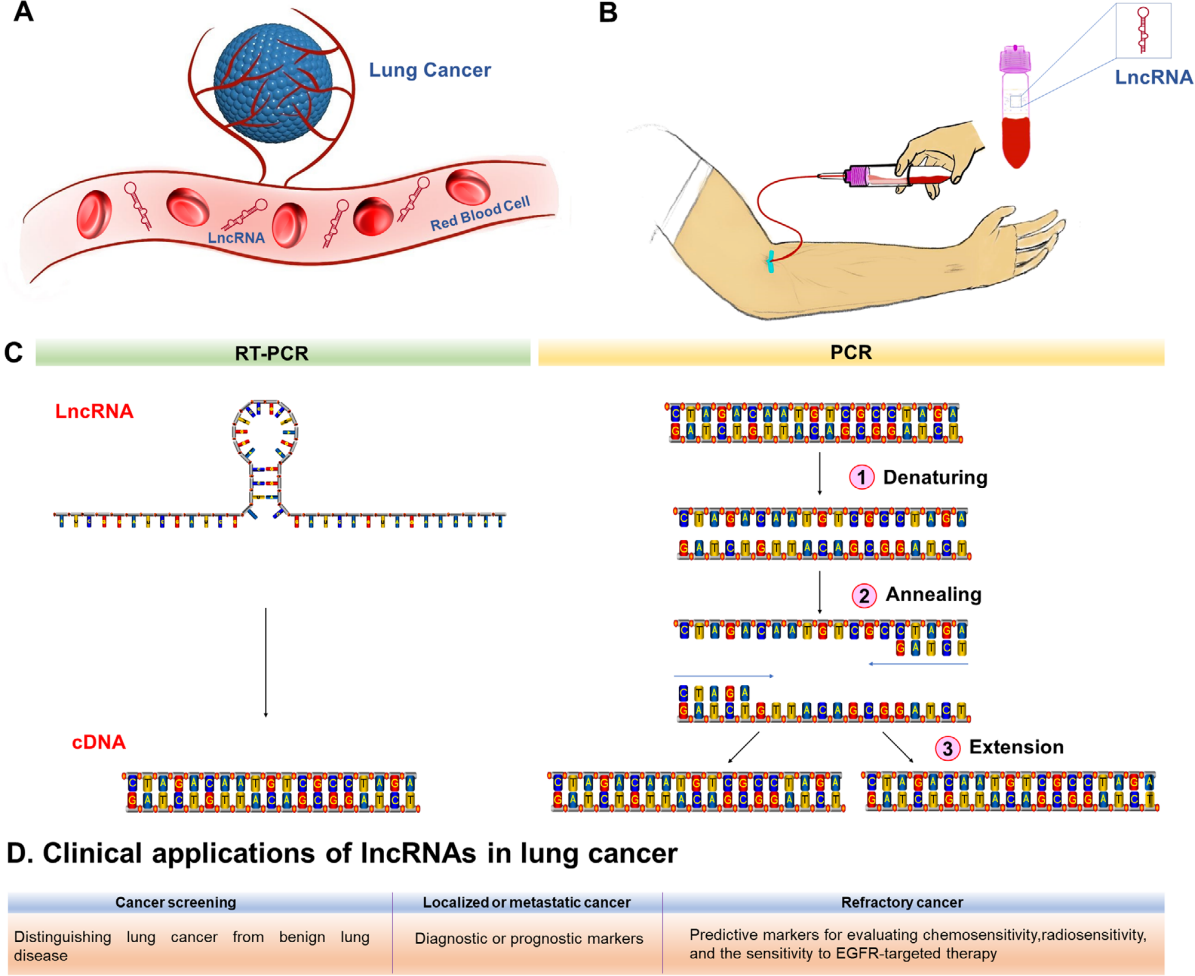
Analysis of lncRNA and clinical applications. A, In lung cancer, tumors with migration potential activate angiogenesis. LncRNAs are released from tumor cells and circulate in the blood. B, The blood sample is drawn from patients. After the whole blood is centrifuged, the bottom of the tube is red blood cells, and lncRNAs are extracted from the supernatant. C, Reverse transcription and amplification of cDNA by PCR. D, Clinical applications of lncRNAs in lung cancer

Generally, after LDCT screening, the clinical application of lncRNAs as an auxiliary diagnostic tool for LDCT is as follows (Figure 8): the patient's peripheral blood is collected before surgical resection or chemotherapy/radiotherapy/EGFR‐targeted therapy. LncRNAs are extracted from blood samples. By reverse transcription, cDNA libraries are generated. After amplification of cDNA by PCR, expression data are statistically analyzed. According to the expression characteristics of different lncRNAs, results are then interpreted for use in clinical decision‐making and other applications.^132^

### 9.1 Challenges and perspectives

Abnormal lncRNAs in tumor tissues can be monitored in body fluids, such as whole blood, saliva, and urine. LncRNA‐based liquid biopsy has the advantage of being noninvasive and well‐tolerated by patients.^174^ However, lncRNA‐based liquid biopsy as an auxiliary means of LDCT to screen for early lung cancer also faces some challenges.

Firstly, although ribonuclease is abundant in body fluids, lncRNAs are pack concentrated in exosomes and microvesicles for secretion, conferring resistance to ribonuclease. Therefore, lncRNAs exist stably in body fluids and can be detected.^174^ What's more, Arita et al revealed that lncRNAs only exhibit a small degree of instability under some extreme conditions, such as some freeze‐thaw processes and incubations with elevated temperatures.^175^ According to the results of Ren et al, it was found that the expression level of lncRNAs in heparin plasma was significantly reduced compared to EDTA plasma.^176^ Therefore, the sample collection and lncRNA extraction process needs to be standardized to improve the stability of lncRNAs derived from body fluids.

Secondly, before lncRNAs are used in the clinical detection of early lung cancer, a consensus should be reached on the suitable molecule as an endogenous control.

Thirdly, according to the results of a previous study in our laboratory, through the analysis of data from The Cancer Genome Atlas and Gene Expression Omnibus, eight lncRNAs with diagnostic potential in lung cancer were identified.^177^ Although bioinformatics methods represent an efficient method for the preliminary screening of tumor markers, the potential of screened lncRNAs should be further confirmed with by cytology experiments, xenograft mouse models, and biological specimens derived from lung cancer patients for their clinical applications in lung cancer.

Fourthly, in order to streamline the process of transforming measurements of lncRNAs into clinical applications, the reference interval for the pathogenicity of lncRNA assessment, etc. need to be further considered and established.

Fifthly, the future research trend is to expand the number and diversity of clinical samples used for research and to combine different lncRNAs to form diagnostic and prognostic panels. In such combinatorial panels, the specificity and sensitivity of the assay are improved while preserving the specific contribution of each individual lncRNA.

Although many challenges need to be addressed in the future, lncRNAs have bright prospects as an adjunct to radiographic methods in the clinical management of lung cancer.

## CONFLICT OF INTEREST

The authors declare that there is no conflict of interest that could be perceived as prejudicing the impartiality of the research reported.

## AUTHOR CONTRIBUTIONS

Yu Chen and Youping Deng designed this manuscript. Yu Chen wrote and edited this manuscript and made figures and tables. Yu Chen, Emory Zitello,Youping Deng, and Rui Guo reviewed and revised this manuscript. All authors read and approved the final manuscript.

## Supporting information

Table S1

Table S2

Table S3

Table S4

Table S5

Table S6

## Data Availability

Data sharing is not applicable to this article as no datasets were generated or analyzed during the current study.
