# Supplementary material for: The function of LncRNAs and their role in the prediction, diagnosis, and prognosis of lung cancer
Source: Clin Transl Med. 2021 Apr 5;11(4):e367. doi: 10.1002/ctm2.367 (PMC8021541; doi:10.1002/ctm2.367)
Supplement: Supplementary file 1 — Table S1 [file CTM2-11-e367-s005.docx]

Supplementary Table S1.Diversified biological functions of lncRNAs

| Functions | Categories | Details | Biological effect | Reference |
| --- | --- | --- | --- | --- |
| LncRNAs as ﻿epigenetic regulators | The structure of chromatin | LncRNA CCAT1-L interacts with CTCF and regulates the binding of CTCF to chromatin at the *MYC* locus, thereby enhancing the long-range ﻿chromatin interactions between the *MYC* promoter and its enhancers. | Regulating the development of CRC | ^1^ |
|  |  | LncRNA Khps1 binds to the TSS of SPHK1-B through DNA-RNA triplexes. The ﻿Khps1-associated p300/CBP binds to the SPHK1-B promoter, resulting in high acetylation and decompaction of chromatin, thereby promoting the combination of E2F1 and SPHK1-B promoter to activate the transcription of SPHK1. | Enhancing transcription and inhibiting apoptosis | ^2^ |
|  |  | HOTTIP as a lincRNA interacts with the adjacent target gene *HOXA* through the chromosomal loop to change the conformation of chromatin. | Regulating long-range gene activation | ^3^ |
|  | The histone modification on chromatin | In addition to the different effects of DNA methylation, lncRNA and mRNA have similar epigenetic regulatory characteristics. | Affecting epigenetics regulation | ^4^ |
|  |  | LncRNA HOTAIR binds to GA-rich polypurine DNA motifs at the chromatin locus. PRC2 can trimethylate histone H3K27, while LSD1 can demethylate H3K4. | It contributes to chromatin remodeling and promotes gene silencing | ^5^ |
|  |  | LncRNA FENDRR binds to PRC2 and histone modification complex TrxG/ MLL. Then FENDRR recruits to the promoter Foxf1 and Pitx2, followed by increasing H3K27 trimethylation and H3K4 demethylation. | Regulating lateral mesoderm differentiation, especially the normal development of the heart and the body wall | ^6^ |
|  | Alternative splicing | LncRNA asFGFR2 as a chromatin signature regulates chromatin-dependent FGFR2 alternative splicing by recruiting PRC2 and KDM2a. | Maintaining the diversity of protein produced | ^7^ |
|  | The X-chromosome silencing | LncRNA XIST, RepA, Jpx, Ftx and Tsix are involved in the process of X-inactivation. | Regulating early embryo development and tumorigenesis | ^8^ |
|  | The X-chromosome dosage compensation | LncRNA *roX1* and *roX2* as important components of MSL complex contribute to dosage compensation of the X chromosome. | Avoiding aneuploidy and male lethality | ^9^ |
| LncRNAs as transcriptional regulators | Promoter/Enhancer | LncRNA BISPR and BST2 mRNA are co-expressed on a bidirectional promoter. The promoter region shared by BISPR and BST2 is found to overlap active histone markers. The BISPR/BST2 locus overlaps the enhancer region. | Responding to interferon | ^10^ |
|  |  | The lncRNA PVT1 promoter competes for enhancers within the gene *PVT1*, thereby inhibiting *MYC* transcription *in cis*. | Anti-cancer effect | ^11^ |
|  |  | LncRNA APTR associates with the *p21* promoter through the embedded Alu (c-Alu) element in APTR.APTR recruits PRC2, catalyzing the histone H3K27me3 of chromatin to *trans*-repress the *p21* promoter, and ultimately promotes the proliferation of GBM cells. | Promoting the proliferation of cancer cells | ^12^ |
|  | Transcription factor | LncRNA ANCR with anti-differentiation function is regarded as a repressor. LncRNA TINCR with differentiation induction function is regarded as an activator. Both can regulate the downstream transcription factor MAF: MAFB. | Regulating epidermal differentiation | ^13^ |
|  |  | In human type 2 diabetes, the down-regulated lncRNA PLUTO attenuates the transcription factor PDX1 promoter targeting PLUTO enhancer cluster, inhibiting the transcription of human islet β cells. | Reducing insulin secretion and impairing blood sugar balance in the body | ^14^ |
|  |  | Histone H3K79 methyltransferase binds to lncRNA PRNCR1, then regulates AR in K349 methylation. K349 methylation and ﻿K631/634 acetylation facilitate the recruitment of PCGEM1 to AR. LncRNA PCGEM1 and PRNCR1 promote the binding of pygopus2 and AR to the enhancer region of target gene, and enhance enhancer-promoter interaction. | Promoting androgen receptor transcription and prostate cancer cell proliferation | ^15^ |
|  |  | LncRNA MALAT1 regulates the alternative splicing of the oncogenic transcription factor B-MYB pre-mRNAs. | Controlling the cell cycle progression | ^16^ |
|  |  | YY1 activates the transcription of lncRNA PVT1 by targeting PVT1 and binding its promoter region. | Leading to the migration and invasion of lung cancer | ^17^ |
|  |  | LncRNA FOXC2-AS1 stabilizes FOXC2 mRNA by forming an double-stranded RNA with FOXC2 mRNA, and up-regulates the expression of *FOXC2* and *ABCB1.* | Promoting the resistance of osteosarcoma cells to doxorubicin and correlates with poor prognosis | ^18^ |
|  |  | LncRNA *Braveheart* interacts with the transcription factor zinc-finger protein CNBP through the G-rich motif, inhibits CNBP which is a negative regulator of heart development. | Regulating cardiovascular lineage commitment | ^19^ |
| LncRNAs as post-transcriptional regulators | LncRNA as a precursor to small RNA | In rice, 21-nt and 24-nt phasiRNAs are derived from lncRNAs. | Regulating male sterility | ^20^ |
|  |  | In the *arabidopsis thaliana*, DCL1-dependent sRNAs are derived from the local long-stem structures of lncRNAs. | Potential regulatory activity | ^21^ |
|  |  | 268-nt RMRP is the source of two short RNAs (∼20 nt), RMRP-S1 and RMRP-S2. | Regulating the genes that have an effect on skeletal development, hair development and hematopoietic cell differentiation | ^22^ |
|  |  | LncRNA H19 harbors the sequence for miR-675. | Both H19 and miR-675 are involved in the regulation of chondrocyte metabolism and can be used as indicators to diagnose whether the metabolism is balanced in OA | ^23^ |
|  |  | LncRNA MIR100HG is the source of miR-100 and miR-125b. | Patients with head and neck squamous cell cancer and colorectal cancer develop resistance to cetuximab | ^24^ |
|  |  | MiR-31 can be transcribed from the intron sequence of LOC554202. | Hypermethylation of the promoter regulates the expression levels of LOC554202 and miR-31,which contributes to the invasion-metastasis cascade of triple negative breast cancer | ^25^ |
|  |  | MALAT1 derived MD-miniRNA is identified in plasma samples from patients with PCa. | MD-miniRNA can be used as a novel plasma-based biomarker to improve the diagnostic accuracy of PCa | ^26^ |
|  |  | The nuclear-retained ncRNA with a short poly (A) tail-like moiety and a small tRNA-like cytoplasmic RNA originated from MALAT1. | MascRNA can be used as a signaling molecule and a tRNA mimic | ^27^ |
|  | LncRNAs regulate mRNA stability | LncRNA MACC1-AS1 combined with mRNA to form RNA-RNA complexes to stabilize MACC1 mRNA. | Promoting the survival of GC cells and ultimately leading to the development of GC | ^28^ |
|  |  | LncRNA THOR binds to the 3′UTR of SOX9 mRNA, which stabilizes the mRNA by forming RNA duplexes. | Promoting the ﻿stemness and drug resistance of GC cells | ^29^ |
|  |  | Up-regulated lncRNA TINCR promotes STAU1-mediated mRNA decay, reducing the half-life of KLF2 mRNA. | Antagonizing the tumor suppressor function of gene KLF2, and finally promotes the progress of GC | ^30^ |
|  |  | LncRNA GHET1 enhances the interaction between RNA-binding protein IGF2BP1 and *c-Myc* mRNA by binding to IGF2BP1, thereby inhibiting the degradation of *c-Myc* mRNA. | Promoting the proliferation of GC cells | ^31^ |
|  |  | LncRNA PDCD4-AS1 combines with PDCD4 mRNA to form an RNA duplex, and weakens the interaction between PDCD4 mRNA and mRNA decay factor HuR to stabilize PDCD4 mRNA. | Suppressing the progression of triple-negative breast cancer | ^32^ |
| LncRNAs as ceRNAs regulate miRNAs | Acting as "sponge"/ "decoy" for miRNAs | LncRNA AK015322 interacts with the RISC. The miR-19b-3p binds to lncRNA AK015322 and is cleaved by the protein Argonaute in RISC, and then silences the target gene Ets-variant 5. | Promoting the self-renewal of mouse spermatogonial stem cell | ^33^ |
|  |  | In C13-PB cells, lncRNA H19 acts as a sponge of micRNA *let-7b*, triggering the process of EMT. In C13-PT and C13-LM cells, lncRNA *H19* acts as a sponge of miR-200b/c, triggering the process of MET. | Promoting breast cancer cells metastasis | ^34^ |
|  |  | In breast cancer cells, the decoy mechanism of lncRNA PVT1 is turned off, which be unable to inhibit miR-200 family members. In normal breast cells, the decoy mechanism of lncRNA PVT1 is switched on, which can inhibit miR-200 family members and affect the expression of downstream mRNAs. | In breast cancer cells, lncRNA PVT1 inhibits apoptosis. In normal breast cells, lncRNA PVT1 exerts anticancer effects. | ^35,36^ |

Abbreviations

ABCB1: ATP Binding Cassette Subfamily B Member 1

APTR: Alu-Mediated CDKN1A/P21 Transcriptional Regulator

AR: Androgen Receptor

BISPR: BST2 IFN-Stimulated Positive Regulator

B-MYB: Myb-Related Protein B

BST2: Bone Marrow Stromal Cell Antigen 2

CBP: CREB-Binding Protein

CCAT1: Colon Cancer Associated Transcript 1

CNBP: CCHC-Type Zinc Finger Nucleic Acid Binding Protein

CRC: Colorectal Cancer

CTCF: CCCTC-Binding Factor

E2F1: E2F Transcription Factor 1

EMT: Epithelial-Mesenchymal Transition

FENDRR: FOXF1 Adjacent Non-Coding Developmental Regulatory RNA

Foxf1: Forkhead Box F1

FOXC2: Forkhead Box C2

FOXC2-AS1: FOXC2 Antisense RNA 1

GBM: Glioblastoma Multiforme

GC: Gastric Cancer

GHET1: Gastric Carcinoma Proliferation Enhancing Transcript 1

H3K27: Histone H3 on lysine 27

H3K27me3: Trimethylation at lysine 27 of Histone H3

H3K79: Histone H3 on lysine 79

HOTAIR: HOX Transcript Antisense RNA

HOTTIP: HOXA distal transcript antisense RNA

IGF2BP1: Insulin Like Growth Factor 2 MRNA Binding Protein 1

KDM2a: Lysine Demethylase 2A

KLF2: Kruppel Like Factor 2

LincRNA: Long intergenic non-coding RNA

LSD1: Lysine-Specific Histone Demethylase 1A

MACC1: Metastasis Associated In Colon Cancer 1

MACC1-AS1: MACC1 Antisense RNA 1

MALAT1: Metastasis Associated Lung Adenocarcinoma Transcript 1

MD-miniRNA: MALAT1 derived-miniRNA

MET: Mesenchymal-Epithelial Transition

MIR100HG: Mir-100-Let-7a-2-Mir-125b-1 Cluster Host Gene

MLL: Mixed-Lineage Leukemia Protein

MSL: Male Specific Lethal

ncRNA: noncoding RNA

OA: Osteoarthritis

PCGEM1: PCGEM1 Prostate-Specific Transcript

PCa: Prostate Cancer

PDCD4: Programmed Cell Death 4

PDCD4-AS1: PDCD4 Antisense RNA 1

PDX1: Pancreatic And Duodenal Homeobox 1

Pitx2: Paired Like Homeodomain 2

PLUTO: PDX1 Associated LncRNA, Upregulator Of Transcription

PRC2: Polycomb Repressive Complex 2

PRNCR1: Prostate Cancer Associated Non-Coding RNA 1

PVT1: Plasmacytoma variant translocation 1

RMRP: RNA Component Of Mitochondrial RNA Processing Endoribonuclease

RISC: RNA-Induced Silencing Complex

SOX9: SRY-Box Transcription Factor 9

SPHK1: Sphingosine Kinase 1

STAU1: Staufen Double-Stranded RNA Binding Protein 1

THOR: Testis Associated Oncogenic LncRNA

TrxG: Trithorax-Group Proteins

tRNA: transfer RNA

﻿TSS: Transcription start site

XIST: X inactive specific transcript

YY1:Yin Yang-1

Supplementary References

1. Xiang JF, Yin QF, Chen T, et al. Human colorectal cancer-specific CCAT1-L lncRNA regulates long-range chromatin interactions at the MYC locus. *Cell Res.* 2014;24(5):513-531.

2. Postepska-Igielska A, Giwojna A, Gasri-Plotnitsky L, et al. LncRNA Khps1 Regulates Expression of the Proto-oncogene SPHK1 via Triplex-Mediated Changes in Chromatin Structure. *Mol Cell.* 2015;60(4):626-636.

3. Wang KC, Yang YW, Liu B, et al. A long noncoding RNA maintains active chromatin to coordinate homeotic gene expression. *Nature.* 2011;472(7341):120-124.

4. Sati S, Ghosh S, Jain V, Scaria V, Sengupta S. Genome-wide analysis reveals distinct patterns of epigenetic features in long non-coding RNA loci. *Nucleic Acids Res.* 2012;40(20):10018-10031.

5. Bhan A, Mandal SS. LncRNA HOTAIR: A master regulator of chromatin dynamics and cancer. *Biochim Biophys Acta.* 2015;1856(1):151-164.

6. Grote P, Wittler L, Hendrix D, et al. The tissue-specific lncRNA Fendrr is an essential regulator of heart and body wall development in the mouse. *Dev Cell.* 2013;24(2):206-214.

7. Gonzalez I, Munita R, Agirre E, et al. A lncRNA regulates alternative splicing via establishment of a splicing-specific chromatin signature. *Nat Struct Mol Biol.* 2015;22(5):370-376.

8. Froberg JE, Yang L, Lee JT. Guided by RNAs: X-inactivation as a model for lncRNA function. *J Mol Biol.* 2013;425(19):3698-3706.

9. Samata M, Akhtar A. Dosage Compensation of the X Chromosome: A Complex Epigenetic Assignment Involving Chromatin Regulators and Long Noncoding RNAs. *Annu Rev Biochem.* 2018;87:323-350.

10. Kambara H, Gunawardane L, Zebrowski E, et al. Regulation of Interferon-Stimulated Gene BST2 by a lncRNA Transcribed from a Shared Bidirectional Promoter. *Front Immunol.* 2014;5:676.

11. Cho SW, Xu J, Sun R, et al. Promoter of lncRNA Gene PVT1 Is a Tumor-Suppressor DNA Boundary Element. *Cell.* 2018;173(6):1398-1412 e1322.

12. Negishi M, Wongpalee SP, Sarkar S, et al. A new lncRNA, APTR, associates with and represses the CDKN1A/p21 promoter by recruiting polycomb proteins. *PLoS One.* 2014;9(4):e95216.

13. Lopez-Pajares V, Qu K, Zhang J, et al. A LncRNA-MAF:MAFB transcription factor network regulates epidermal differentiation. *Dev Cell.* 2015;32(6):693-706.

14. Akerman I, Tu Z, Beucher A, et al. Human Pancreatic beta Cell lncRNAs Control Cell-Specific Regulatory Networks. *Cell Metab.* 2017;25(2):400-411.

15. Yang L, Lin C, Jin C, et al. lncRNA-dependent mechanisms of androgen-receptor-regulated gene activation programs. *Nature.* 2013;500(7464):598-602.

16. Tripathi V, Shen Z, Chakraborty A, et al. Long noncoding RNA MALAT1 controls cell cycle progression by regulating the expression of oncogenic transcription factor B-MYB. *PLoS Genet.* 2013;9(3):e1003368.

17. Huang T, Wang G, Yang L, et al. Transcription Factor YY1 Modulates Lung Cancer Progression by Activating lncRNA-PVT1. *DNA Cell Biol.* 2017;36(11):947-958.

18. Zhang CL, Zhu KP, Ma XL. Antisense lncRNA FOXC2-AS1 promotes doxorubicin resistance in osteosarcoma by increasing the expression of FOXC2. *Cancer Lett.* 2017;396:66-75.

19. Xue Z, Hennelly S, Doyle B, et al. A G-Rich Motif in the lncRNA Braveheart Interacts with a Zinc-Finger Transcription Factor to Specify the Cardiovascular Lineage. *Mol Cell.* 2016;64(1):37-50.

20. Arikit S, Zhai J, Meyers BC. Biogenesis and function of rice small RNAs from non-coding RNA precursors. *Curr Opin Plant Biol.* 2013;16(2):170-179.

21. Ma X, Shao C, Jin Y, Wang H, Meng Y. Long non-coding RNAs: a novel endogenous source for the generation of Dicer-like 1-dependent small RNAs in Arabidopsis thaliana. *RNA Biol.* 2014;11(4):373-390.

22. Rogler LE, Kosmyna B, Moskowitz D, et al. Small RNAs derived from lncRNA RNase MRP have gene-silencing activity relevant to human cartilage-hair hypoplasia. *Hum Mol Genet.* 2014;23(2):368-382.

23. Steck E, Boeuf S, Gabler J, et al. Regulation of H19 and its encoded microRNA-675 in osteoarthritis and under anabolic and catabolic in vitro conditions. *J Mol Med (Berl).* 2012;90(10):1185-1195.

24. Lu Y, Zhao X, Liu Q, et al. lncRNA MIR100HG-derived miR-100 and miR-125b mediate cetuximab resistance via Wnt/beta-catenin signaling. *Nat Med.* 2017;23(11):1331-1341.

25. Augoff K, McCue B, Plow EF, Sossey-Alaoui K. miR-31 and its host gene lncRNA LOC554202 are regulated by promoter hypermethylation in triple-negative breast cancer. *Mol Cancer.* 2012;11:5.

26. Ren S, Wang F, Shen J, et al. Long non-coding RNA metastasis associated in lung adenocarcinoma transcript 1 derived miniRNA as a novel plasma-based biomarker for diagnosing prostate cancer. *Eur J Cancer.* 2013;49(13):2949-2959.

27. Wilusz JE, Freier SM, Spector DL. 3' end processing of a long nuclear-retained noncoding RNA yields a tRNA-like cytoplasmic RNA. *Cell.* 2008;135(5):919-932.

28. Zhao Y, Liu Y, Lin L, et al. The lncRNA MACC1-AS1 promotes gastric cancer cell metabolic plasticity via AMPK/Lin28 mediated mRNA stability of MACC1. *Mol Cancer.* 2018;17(1):69.

29. Song H, Xu Y, Shi L, et al. LncRNA THOR increases the stemness of gastric cancer cells via enhancing SOX9 mRNA stability. *Biomed Pharmacother.* 2018;108:338-346.

30. Xu TP, Liu XX, Xia R, et al. SP1-induced upregulation of the long noncoding RNA TINCR regulates cell proliferation and apoptosis by affecting KLF2 mRNA stability in gastric cancer. *Oncogene.* 2015;34(45):5648-5661.

31. Yang F, Xue X, Zheng L, et al. Long non-coding RNA GHET1 promotes gastric carcinoma cell proliferation by increasing c-Myc mRNA stability. *FEBS J.* 2014;281(3):802-813.

32. Jadaliha M, Gholamalamdari O, Tang W, et al. A natural antisense lncRNA controls breast cancer progression by promoting tumor suppressor gene mRNA stability. *PLoS Genet.* 2018;14(11):e1007802.

33. Hu K, Zhang J, Liang M. LncRNA AK015322 promotes proliferation of spermatogonial stem cell C18-4 by acting as a decoy for microRNA-19b-3p. *In Vitro Cell Dev Biol Anim.* 2017;53(3):277-284.

34. Zhou W, Ye XL, Xu J, et al. The lncRNA H19 mediates breast cancer cell plasticity during EMT and MET plasticity by differentially sponging miR-200b/c and let-7b. *Sci Signal.* 2017;10(483).

35. Paci P, Colombo T, Farina L. Computational analysis identifies a sponge interaction network between long non-coding RNAs and messenger RNAs in human breast cancer. *BMC Systems Biology.* 2014;8(1).

36. Conte F, Fiscon G, Chiara M, Colombo T, Farina L, Paci P. Role of the long non-coding RNA PVT1 in the dysregulation of the ceRNA-ceRNA network in human breast cancer. *PLoS One.* 2017;12(2):e0171661.
