# Supplementary material for: The function of LncRNAs and their role in the prediction, diagnosis, and prognosis of lung cancer
Source: Clin Transl Med. 2021 Apr 5;11(4):e367. doi: 10.1002/ctm2.367 (PMC8021541; doi:10.1002/ctm2.367)
Supplement: Supplementary file 2 — Table S2 [file CTM2-11-e367-s006.docx]

Supplementary Table S2. LncRNAs as predictive markers of ﻿chemosensitivity in lung cancer

| Official symbol | Ensemble/﻿GenBank accession no. | Genomic location | Description of the lncRNA | Types of lung cancer | Expression in drug-resistant cells of lung cancer | Function in tumorigenesis | Mechanism of action | Chemotherapy drugs | Effect on chemosensitivity in lung cancer | Reference |
| --- | --- | --- | --- | --- | --- | --- | --- | --- | --- | --- |
| XIST | ENSG00000229807 | [Chromosome X: 73,820,649-73,852,723](https://uswest.ensembl.org/Homo_sapiens/Location/View?db=core;g=ENSG00000229807;r=X:73820649-73852723) reverse strand | X inactive specific transcript | NSCLC | Upregulated | ﻿Oncogene | Promoting autophagy through lncRNA-XIST/miR-17 axis | Cisplatin | ﻿Upregulated lncRNA-XIST reduces chemosensitivity | ^1^ |
|  |  |  |  | LAD |  |  | Regulating let-7i/BAG-1 axis |  |  | ^2^ |
|  |  |  |  | NSCLC |  |  | ﻿Regulating the ﻿translocation of SMAD2 and transcription of p53 and NLRP3 |  |  | ^3^ |
|  |  |  |  | NSCLC |  |  | Regulating miR-144-3p/MDR1 and MRP1 axis |  |  | ^4^ |
| MALAT1 | ENSG00000251562 | [Chromosome 11: 65,497,688-65,506,516](https://uswest.ensembl.org/Homo_sapiens/Location/View?db=core;g=ENSG00000251562;r=11:65497688-65506516) forward strand | Metastasis associated lung adenocarcinoma transcript 1 | NSCLC | Upregulated | Oncogene | Regulating miR-197-3p/p120 Catenin Axis | Cisplatin, adriamycin, gefitinib and paclitaxel | Upregulated lncRNA MALAT1 reduces chemosensitivity | ^5^ |
|  |  |  |  |  |  |  | Upregulating MRP1 and MDR1 through STAT3 activation | Cisplatin  ﻿ |  | ^6^ |
|  |  |  |  |  |  |  | Regulating MALAT1-miR-101-SOX9 axis via Wnt signaling pathway |  |  | ^7^ |
|  |  |  |  |  |  |  | Regulating miR-101-3p/MCL1 axis |  |  | ^8^ |
|  |  |  |  |  |  |  | Regulating miR-200b/E2F3 & ZEB1 axis | Docetaxel |  | ^9^ |
| MEG3 | ENSG00000214548 | [Chromosome 14: 100,779,410-100,861,031](https://uswest.ensembl.org/Homo_sapiens/Location/View?db=core;g=ENSG00000214548;r=14:100779410-100861031) forward strand | Maternally expressed gene 3 | LAD | Downregulated | Tumor suppressor | Regulating the expression of p53 and Bcl-xl | Cisplatin | Downregulated lncRNA MEG3 reduces chemosensitivity | ^10^ |
|  |  |  |  | NSCLC |  |  | Regulating miR-21-5p/SOX7 axis |  |  | ^11^ |
|  |  |  |  | NSCLC |  |  | Regulating WNT/β-catenin signaling pathway |  |  | ^12^ |
|  |  |  |  | NSCLC |  |  | Regulating the level of autophagy | Vincristine |  | ^13^ |
| ﻿GAS5 | ENSG00000234741 | [Chromosome 1: 173,858,559-173,868,882](https://uswest.ensembl.org/Homo_sapiens/Location/View?db=core;g=ENSG00000234741;r=1:173858559-173868882) reverse strand | Growth arrest specific 5 | NSCLC | Downregulated | Tumor suppressor | Regulating miR-21/PTEN axis | Cisplatin | Downregulated lncRNA GAS5 reduces chemosensitivity | ^14^ |
|  |  |  |  |  |  |  | Regulating the level of autophagy |  |  | ^15^ |
| SFTA1P | ENSG00000225383 | [Chromosome 10: 10,784,437-10,795,047](https://uswest.ensembl.org/Homo_sapiens/Location/View?db=core;g=ENSG00000225383;r=10:10784437-10795047) reverse strand | Surfactant associated 1 | LSCC | Downregulated | Tumor suppressor | ﻿Regulating hnRNP-U/GADD45A axis | Cisplatin | Downregulated lncRNA SFTA1P reduces chemosensitivity | ^16^ |
| HOTTIP | ENSG00000243766 | [Chromosome 7: 27,198,575-27,207,259](https://uswest.ensembl.org/Homo_sapiens/Location/View?db=core;g=ENSG00000243766;r=7:27198575-27207259) forward strand | HOXA distal transcript antisense RNA | SCLC | Upregulated | Oncogene | Regulating miR-216a/BCL-2 Axis | ﻿Cisplatin, Etoposide (VP-16), ﻿adriamycin (ADM) | Upregulated lncRNA HOTTIP  reduces chemosensitivity | ^17^ |
| HOTAIR | ENSG00000228630 | [Chromosome 12: 53,962,308-53,974,956](https://uswest.ensembl.org/Homo_sapiens/Location/View?db=core;g=ENSG00000228630;r=12:53962308-53974956) reverse strand | Homeobox transcript antisense RNA | SCLC | Upregulated | Oncogene | ﻿Methylated HOXA1 by increasing the expression of DNMT1 and DNMT3b | Cisplatin, Etoposide (VP-16),﻿adriamycin (ADM) | Upregulated lncRNA HOTAIR reduces chemosensitivity | ^18^ |
|  |  |  |  |  |  |  | Regulating NF-κB signaling pathway |  |  | ^19^ |
| CCAT1 | 100507056 | Chromosome 8: 127,207,382-  127,219,268 Minus strand;Chromosome8:  128,219,629-  128,231,333 Minus strand | Colon cancer-associated transcript-1; CARLo-5 | NSCLC | Upregulated | Oncogene | Regulating miR-130a-3p/SOX4 axis | Cisplatin | Upregulated lncRNA CCAT1 reduces chemosensitivity | ^20^ |
|  |  |  |  | LAD |  |  | Regulating let-7c/Bcl-xl axis | Docetaxel |  | ^21^ |
| TATDN1 | ENSG00000147687 | [Chromosome 8: 124,488,485-124,539,458](https://uswest.ensembl.org/Homo_sapiens/Location/View?db=core;g=ENSG00000147687;r=8:124488485-124539458) reverse strand | TatD DNase domain containing 1 | NSCLC | Upregulated | Oncogene | Regulating miR-451/TRIM66 axis | Cisplatin | Upregulated lncRNA TATDN1 reduces chemosensitivity | ^22^ |
| BLACAT1 | ENSG00000281406 | [Chromosome 1: 205,434,885-205,457,091](https://uswest.ensembl.org/Homo_sapiens/Location/View?db=core;g=ENSG00000281406;r=1:205434885-205457091) reverse strand | Bladder cancer associated transcript 1 | NSCLC | Upregulated | Oncogene | Regulating the level of autophagy through the miR‑17/ATG7 signaling pathway | Cisplatin | Upregulated lncRNA BLACAT1 reduces chemosensitivity | ^23^ |
| TUG1 | ENSG00000253352 | [Chromosome 22: 30,969,245-30,979,395](https://uswest.ensembl.org/Homo_sapiens/Location/View?db=core;g=ENSG00000253352;r=22:30969245-30979395) forward strand | Taurine up-regulated 1 | SCLC | Upregulated | Oncogene | Regulating LIMK2b via EZH2 | Cisplatin, Etoposide (VP-16), ﻿adriamycin (ADM) | Upregulated lncRNA TUG1  reduces chemosensitivity | ^24^ |
|  |  |  |  | NSCLC | Downregulated | Tumor suppressor | Regulating miR-221/PTEN axis | Cisplatin | Downregulated lncRNA TUG1 reduces chemosensitivity | ^25^ |
| H19 | ENSG00000130600 | [Chromosome 11: 1,995,176-2,001,470](https://uswest.ensembl.org/Homo_sapiens/Location/View?db=core;g=ENSG00000130600;r=11:1995176-2001470) reverse strand | H19 imprinted maternally expressed transcript | LAD | Upregulated | Oncogene | ﻿Regulating cell cycle | Cisplatin | Upregulated lncRNA H19 reduces chemosensitivity | ^26^ |
| TRPM2-AS | ENSG00000230061 | [Chromosome 21: 44,414,588-44,425,272](https://uswest.ensembl.org/Homo_sapiens/Location/View?db=core;g=ENSG00000230061;r=21:44414588-44425272) reverse strand | TRPM2 antisense RNA | NSCLC | Upregulated | Oncogene | Regulating ﻿p53-p66^shc^ pathway and altered cell cycle distribution | Cisplatin | Upregulated lncRNA TRPM2-AS reduces chemosensitivity | ^27^ |
| FOXD2-AS1 | ENSG00000237424 | [Chromosome 1: 47,432,133-47,434,641](https://uswest.ensembl.org/Homo_sapiens/Location/View?db=core;g=ENSG00000237424;r=1:47432133-47434641;t=ENST00000445551) reverse strand | FOXD2 adjacent opposite strand RNA 1 | NSCLC | Upregulated | Oncogene | Regulating miR185-5p/SIX1 axis | Cisplatin | Upregulated lncRNA FOXD2-AS1 reduces chemosensitivity | ^28^ |
| PILAR1 | ENSG00000233461 | [Chromosome 1: 231,520,729-231,528,618](https://uswest.ensembl.org/Homo_sapiens/Location/View?db=core;g=ENSG00000233461;r=1:231520729-231528618) reverse strand | ﻿Prognostic in Lung Adenocarcinoma LncRNA 1 | LAD | Upregulated | Oncogene | ﻿Regulating genes related to DNA damage and immune activity | ﻿Etoposide (VP-16) | Upregulated lncRNA PILAR1 reduces chemosensitivity | ^29^ |
| LINC00173 | ENSG00000196668 | [Chromosome 12: 116,533,422-116,536,518](https://uswest.ensembl.org/Homo_sapiens/Location/View?db=core;g=ENSG00000196668;r=12:116533422-116536518) forward strand | Long intergenic non-protein coding RNA 173 | SCLC | Upregulated | Oncogene | Regulating miR-218/Etk axis | Cisplatin, Etoposide (VP-16), ﻿adriamycin (ADM) | Upregulated LINC00173 reduces chemosensitivity | ^30^ |
| BCYRN1 | ENSG00000236824 | [Chromosome 2: 47,335,315-47,335,514](https://uswest.ensembl.org/Homo_sapiens/Location/View?db=core;g=ENSG00000236824;r=2:47335315-47335514;t=ENST00000418539) forward strand | Brain Cytoplasmic RNA 1; brain cytoplasmic 200 (BC200) | NSCLC | Upregulated | Oncogene | Regulating PI3K/AKT pathway | Cisplatin | Upregulated lncRNA BCYRN1 reduces chemosensitivity | ^31^ |
| KCNQ1OT1 | ENSG00000269821 | [Chromosome 11: 2,608,328-2,699,994](https://uswest.ensembl.org/Homo_sapiens/Location/View?db=core;g=ENSG00000269821;r=11:2608328-2699994;t=ENST00000597346) reverse strand | KCNQ1 opposite strand/antisense transcript 1 | LAD | Upregulated | Oncogene | Regulating the expression of MDR1 | Paclitaxel | Upregulated lncRNA KCNQ1OT1 reduces chemosensitivity | ^32^ |
| LncRNA-ATB | 114004396 | Chromosome 14 | Long non-coding RNA activated by TGF-β | LAD | Upregulated | Oncogene | Regulating miR-200a/β-Catenin axis | Cisplatin | Upregulated lncRNA-ATB reduces chemosensitivity | ^33^ |
| CASC2 | ENSG00000177640 | [Chromosome 10: 118,046,279-118,210,158](https://uswest.ensembl.org/Homo_sapiens/Location/View?db=core;g=ENSG00000177640;r=10:118046279-118210158) forward strand | Cancer susceptibility 2 | NSCLC | Downregulated | Tumor suppressor | Regulating miR-18a/IRF-2 axis | Cisplatin | Downregulated lncRNA CASC2 reduces chemosensitivity | ^34^ |
| CASC8 | ENSG00000246228 | [Chromosome 8: 127,289,817-127,482,139](https://uswest.ensembl.org/Homo_sapiens/Location/View?db=core;g=ENSG00000246228;r=8:127289817-127482139) reverse strand | Cancer susceptibility 8 | LC | N/A | N/A | The SNP of lncRNA CASC8: rs10505477 | ﻿Platinum with gemcitabine (GP), etoposide (EP), pemetrexed (PP), docetaxel (DP), and paclitaxel (TP), and other platinum-based regimens (platinum with irinotecan or navelbine). | ﻿SNP rs10505477 is closely related to platinum-based chemotherapy response | ^35^ |
| FENDRR | ENSG00000268388 | [Chromosome 16: 86,474,529-86,509,099](https://uswest.ensembl.org/Homo_sapiens/Location/View?db=core;g=ENSG00000268388;r=16:86474529-86509099) reverse strand | FOXF1 adjacent non-coding  developmental regulatory RNA | NSCLC | Downregulated | Tumor suppressor | ﻿Regulating the expression of ABCC10 | Cisplatin | Downregulated lncRNA FENDRR  reduces chemosensitivity | ^36^ |
| HOXA-AS3 | ENSG00000254369 | [Chromosome 7: 27,129,977-27,155,928](https://uswest.ensembl.org/Homo_sapiens/Location/View?db=core;g=ENSG00000254369;r=7:27129977-27155928) forward strand | HOXA cluster antisense RNA 3 | NSCLC | Upregulated | Oncogene | HOXA-AS3 interacts with HOXA3 and downregulates HOXA3 | ﻿Cisplatin | Upregulated lncRNA HOXA-AS3 reduces chemosensitivity | ^37^ |
| TP53TG1 | ENSG00000182165 | [Chromosome 7: 87,322,943-87,345,528](https://uswest.ensembl.org/Homo_sapiens/Location/View?db=core;g=ENSG00000182165;r=7:87322943-87345528) reverse strand | TP53 target 1 | NSCLC | Downregulated | Tumor suppressor | Regulating miR-18a/PTEN axis | Cisplatin | Downregulated lncRNA TP53TG1 reduces chemosensitivity | ^38^ |
| LINC00221 | ENSG00000270816 | [Chromosome 14: 106,482,435-106,521,073](https://uswest.ensembl.org/Homo_sapiens/Location/View?db=core;g=ENSG00000270816;r=14:106482435-106521073) forward strand | Long intergenic non-protein coding RNA 221 | NSCLC | Upregulated | Oncogene | Regulating miR-519/ZBTB5 axis | Cisplatin | Upregulated LINC00221 reduces chemosensitivity | ^39^ |
| HOXA11-AS | ENSG00000240990 | [Chromosome 7: 27,184,507-27,189,298](https://uswest.ensembl.org/Homo_sapiens/Location/View?db=core;g=ENSG00000240990;r=7:27184507-27189298) forward strand | HOXA11 antisense RNA | LAD | Upregulated | Oncogene | Regulating miR‐454‐3p/Stat3 axis | Cisplatin | Upregulated lncRNA HOXA11-AS reduces chemosensitivity | ^40^ |
| AK126698 | ENSG00000203601 | [Chromosome 1: 168,903,905-169,087,005](https://uswest.ensembl.org/Homo_sapiens/Location/View?db=core;g=ENSG00000203601;r=1:168903905-169087005;t=ENST00000366408) reverse strand | LINC00970 | NSCLC | Downregulated | Tumor suppressor | Regulating the activation of Wnt pathway | Cisplatin | Downregulated lncRNA AK126698 reduces chemosensitivity | ^41^ |
| EGFR‑AS1 | ENSG00000224057 | [Chromosome 7: 55,179,750-55,188,934](https://uswest.ensembl.org/Homo_sapiens/Location/View?db=core;g=ENSG00000224057;r=7:55179750-55188934;t=ENST00000442411) reverse strand | EGFR Antisense RNA 1 | NSCLC | Upregulated | Oncogene | Regulating ﻿miR-223/﻿IGF1R axis | Cisplatin and gemcitabine | Upregulated lncRNA EGFR‑AS1 reduces chemosensitivity | ^42^ |
| PVT1 | ENSG00000249859 | [Chromosome 8: 127,794,526-128,187,101](https://uswest.ensembl.org/Homo_sapiens/Location/View?db=core;g=ENSG00000249859;r=8:127794526-128187101) forward strand | Pvt1 oncogene | NSCLC | Upregulated | Oncogene | ﻿Regulating autophagy and apoptosis *via*﻿miR-216b/Beclin-1 axis | Cisplatin | Upregulated lncRNA PVT1 reduces chemosensitivity | ^43^ |
| LINC00485 | ENSG00000258169 | [Chromosome 12: 102,809,280-102,824,399](https://uswest.ensembl.org/Homo_sapiens/Location/View?db=core;g=ENSG00000258169;r=12:102809280-102824399;t=ENST00000547179) reverse strand | Long intergenic non-protein coding RNA 485 | LAD | Upregulated | Oncogene | Regulating ﻿miR-195/CHEK1 axis | Cisplatin | Upregulated LINC00485 reduces chemosensitivity | ^44^ |
| NNT-AS1 | ENSG00000248092 | [Chromosome 5: 43,571,594-43,603,230](https://uswest.ensembl.org/Homo_sapiens/Location/View?db=core;g=ENSG00000248092;r=5:43571594-43603230) reverse strand | NNT antisense RNA 1 | NSCLC | Upregulated | Oncogene | Regulating the MAPK/Slug signaling pathway | Cisplatin | Upregulated lncRNA NNT-AS1 reduces chemosensitivity | ^45^ |
| LUCAT1 | ENSG00000248323 | [Chromosome 5: 91,054,834-91,314,547](https://uswest.ensembl.org/Homo_sapiens/Location/View?db=core;g=ENSG00000248323;r=5:91054834-91314547) reverse strand | Lung cancer associated transcript 1 | NSCLC | Upregulated | Oncogene | Upregulating the expression of IGF-2 | Cisplatin | Upregulated lncRNA LUCAT1 reduces chemosensitivity | ^46^ |
| SNHG12 | ENSG00000197989 | [Chromosome 1: 28,578,538-28,583,132](https://uswest.ensembl.org/Homo_sapiens/Location/View?db=core;g=ENSG00000197989;r=1:28578538-28583132) reverse strand | Small nucleolar RNA host gene 12 | NSCLC | Upregulated | Oncogene | ﻿Regulating SNHG12-miR-181a-MAPK/ Slug axis | Cisplatin and paclitaxel | Upregulated lncRNA SNHG12 reduces chemosensitivity | ^47^ |
| AC090204.1-202 | ENST00000500843 | [Chromosome 8: 32,996,178-33,044,855](https://uswest.ensembl.org/Homo_sapiens/Location/View?db=core;g=ENSG00000247134;r=8:32996178-33044855;t=ENST00000500843) forward strand | Novel transcript | LAD | Downregulated | Tumor suppressor | N/A | ﻿Paclitaxel | Downregulated lncRNA AC090204.1-202 reduces chemosensitivity | ^48^ |
| LINC00707 | ENSG00000238266 | [Chromosome 10: 6,779,549-6,879,450](https://uswest.ensembl.org/Homo_sapiens/Location/View?db=core;g=ENSG00000238266;r=10:6779549-6879450) forward strand | Long intergenic non-protein coding RNA 707 | NSCLC | Upregulated | Oncogene | ﻿By sponging miR‑145 | Cisplatin | Downregulated LINC00707 enhances chemosensitivity | ^49^ |
| LINC-ROR | ENSG00000258609 | [Chromosome 18: 57,054,558-57,072,119](https://uswest.ensembl.org/Homo_sapiens/Location/View?db=core;g=ENSG00000258609;r=18:57054558-57072119) reverse strand | Long intergenic non-protein coding RNA, regulator of reprogramming | LAD | Upregulated | Oncogene | Regulating ﻿miR-145/FSCN1 axis | Docetaxel | Upregulated LINC-ROR reduces chemosensitivity | ^50^ |
|  |  |  |  | NSCLC |  |  | Regulating PI3K/Akt/mTOR signaling pathway | Cisplatin | Downregulated LINC-ROR enhances chemosensitivity | ^51^ |
| NEAT1 | ENSG00000245532 | [Chromosome 11: 65,422,774-65,445,540](https://uswest.ensembl.org/Homo_sapiens/Location/View?db=core;g=ENSG00000245532;r=11:65422774-65445540) forward strand | Nuclear paraspeckle assembly transcript 1 | NSCLC | Upregulated | Oncogene | Regulating Akt/mTOR signaling pathway | Paclitaxel | Upregulated lncRNA NEAT1 reduces chemosensitivity | ^52^ |
| SNHG7 | ENSG00000233016 | [Chromosome 9: 136,721,366-136,728,184](https://uswest.ensembl.org/Homo_sapiens/Location/View?db=core;g=ENSG00000233016;r=9:136721366-136728184) reverse strand | Small nucleolar RNA host gene 7 | NSCLC | Upregulated | Oncogene | ﻿Regulating PI3K/AKT signaling pathway | Cisplatin | Upregulated lncRNA SNHG7 reduces chemosensitivity | ^53^ |
| AFAP1-AS1 | ENSG00000272620 | [Chromosome 4: 7,754,077-7,778,928](https://uswest.ensembl.org/Homo_sapiens/Location/View?db=core;g=ENSG00000272620;r=4:7754077-7778928) forward strand | AFAP1 antisense RNA 1 | NSCLC | Upregulated | Oncogene | ﻿Regulating PI3K/AKT signaling pathway | Cisplatin | Upregulated lncRNA AFAP1-AS1 reduces chemosensitivity | ^54^ |
| SPRY4-IT1 | 100642175 | 5q31.3 | SPRY4 intronic transcript 1 | NSCLC | Downregulated | Tumor suppressor | ﻿Regulating MPZL-1 via modulating EMT process | Cisplatin | Upregulated lncRNA SPRY4-IT1 enhances chemosensitivity | ^55^ |
| MIAT | ENSG00000225783 | [Chromosome 22: 26,646,411-26,676,475](https://uswest.ensembl.org/Homo_sapiens/Location/View?db=core;g=ENSG00000225783;r=22:26646411-26676475) forward strand | Myocardial infarction associated transcript | NSCLC | Upregulated | Oncogene | Regulating ﻿miR‑184/SF1 axis | Cisplatin | Downregulated lncRNA MIAT enhances chemosensitivity | ^56^ |
| UCA1 | ENSG00000214049 | [Chromosome 19: 15,828,206-15,836,328](https://uswest.ensembl.org/Homo_sapiens/Location/View?db=core;g=ENSG00000214049;r=19:15828206-15836328) forward strand | Urothelial cancer associated 1 | NSCLC | Upregulated | Oncogene | ﻿Regulating miR‐495/NRF2 axis | Cisplatin | Upregulated lncRNA UCA1 reduces chemosensitivity | ^57^ |
| MSTRG.51053.2 | N/A | N/A | Novel transcript | NSCLC | Upregulated | Oncogene | By Sponging miR-432-5p | Cisplatin | Upregulated lncRNA MSTRG.51053.2 reduces chemosensitivity | ^58^ |

Abbreviations

ABCC10: ATP binding cassette subfamily C member 10

AFAP1: Actin Filament Associated Protein 1

ATG7: Autophagy Related 7

BAG-1: BAG family molecular chaperone regulator 1

CHEK1: Checkpoint Kinase 1

DNMT1: DNA Methyltransferase 1

DNMT3b: DNA Methyltransferase 3 Beta

EGFR: Epidermal Growth Factor Receptor

EMT: Epithelial–mesenchymal transition

EZH2: Enhancer Of Zeste Homolog 2

E2F3: E2F Transcription Factor 3

FOXF1: Forkhead Box F1

FOXD2: Forkhead Box D2

FSCN1: Fascin Actin-Bundling Protein 1

GADD45A: Growth Arrest And DNA Damage Inducible Alpha

hnRNP-U: Heterogeneous Nuclear Ribonucleoprotein U

HOXA1: Homeobox A1

HOXA3: Homeobox A3

HOXA11: Homeobox A11

IGF1R: Insulin-Like Growth Factor 1 Receptor

IGF-2: Insulin Like Growth Factor 2

IRF-2: Interferon Regulatory Factor 2

KCNQ1: Potassium Voltage-Gated Channel Subfamily Q Member 1

LAD: Lung adenocarcinoma

LC: Lung cancer

Let-7c: Lethal-7c

Let-7i: Lethal-7i

LIMK2b: LIM Domain Kinase 2b

LSCC: Lung squamous cell carcinoma

mTOR: mammalian target of rapamycin

MAPK: Mitogen-activated protein kinase

MCL1: Myeloid Cell Leukemia Sequence 1

MDR1: Multidrug resistance 1

MPZL-1: Myelin Protein Zero Like 1

MRP1: Multidrug resistance-associated protein 1

NF-κB: Nuclear factor kappa B

NLRP3: NLR Family Pyrin Domain Containing 3

NNT: Nicotinamide Nucleotide Transhydrogenase

NRF2: Nuclear Factor Erythroid 2-Related Factor 2

NSCLC: Non-small cell lung cancer

N/A: Not available

PI3K: Phosphoinositide 3-kinase

PTEN: Phosphatase And Tensin Homolog

PVT1: Plasmacytoma variant translocation 1

SCLC: Small cell lung cancer

SF1: Splicing Factor 1

SIX1: SIX Homeobox 1

SMAD2: SMAD Family Member 2

SNP: Single nucleotide polymorphisms

SOX4: SRY-Box Transcription Factor 4

SOX9: SRY-Box Transcription Factor 9

SPRY4: Sprouty RTK Signaling Antagonist 4

STAT3: Signal Transducer And Activator Of Transcription 3

TGF-β: Transforming growth factor beta 1

TP53: Tumor Protein P53

TRIM66: Tripartite Motif Containing 66

TRPM2: Transient Receptor Potential Cation Channel Subfamily M Member 2

ZBTB5: Zinc Finger And BTB Domain Containing 5

ZEB1: Zinc Finger E-Box Binding Homeobox 1

Supplementary References

1. Sun W, Zu Y, Fu X, Deng Y. Knockdown of lncRNA-XIST enhances the chemosensitivity of NSCLC cells via suppression of autophagy. *Oncol Rep.* 2017;38(6):3347-3354.

2. Sun J, Pan LM, Chen LB, Wang Y. LncRNA XIST promotes human lung adenocarcinoma cells to cisplatin resistance via let-7i/BAG-1 axis. *Cell Cycle.* 2017;16(21):2100-2107.

3. Xu X, Zhou X, Chen Z, Gao C, Zhao L, Cui Y. Silencing of lncRNA XIST inhibits non-small cell lung cancer growth and promotes chemosensitivity to cisplatin. *Aging (Albany NY).* 2020;12(6):4711-4726.

4. Tian L-J, Wu Y-P, Wang D, et al. Upregulation of Long Noncoding RNA (lncRNA) X-Inactive Specific Transcript (XIST) is Associated with Cisplatin Resistance in Non-Small Cell Lung Cancer (NSCLC) by Downregulating MicroRNA-144-3p. *Medical Science Monitor.* 2019;25:8095-8104.

5. Yang T, Li H, Chen T, Ren H, Shi P, Chen M. LncRNA MALAT1 Depressed Chemo-Sensitivity of NSCLC Cells through Directly Functioning on miR-197-3p/p120 Catenin Axis. *Mol Cells.* 2019;42(3):270-283.

6. Fang Z, Chen W, Yuan Z, Liu X, Jiang H. LncRNA-MALAT1 contributes to the cisplatin-resistance of lung cancer by upregulating MRP1 and MDR1 via STAT3 activation. *Biomedicine & Pharmacotherapy.* 2018;101:536-542.

7. Chen W, Zhao W, Zhang L, et al. MALAT1-miR-101-SOX9 feedback loop modulates the chemo-resistance of lung cancer cell to DDP via Wnt signaling pathway. *Oncotarget.* 2017;8(55):94317-94329.

8. Wang H, Wang L, Zhang G, et al. MALAT1/miR-101-3p/MCL1 axis mediates cisplatin resistance in lung cancer. *Oncotarget.* 2018;9(7):7501-7512.

9. Chen J, Liu X, Xu Y, et al. TFAP2C-Activated MALAT1 Modulates the Chemoresistance of Docetaxel-Resistant Lung Adenocarcinoma Cells. *Mol Ther Nucleic Acids.* 2019;14:567-582.

10. Liu J, Wan L, Lu K, et al. The Long Noncoding RNA MEG3 Contributes to Cisplatin Resistance of Human Lung Adenocarcinoma. *PLoS One.* 2015;10(5):e0114586.

11. Wang P, Chen D, Ma H, Li Y. LncRNA MEG3 enhances cisplatin sensitivity in non-small cell lung cancer by regulating miR-21-5p/SOX7 axis. *Onco Targets Ther.* 2017;10:5137-5149.

12. Xia Y, He Z, Liu B, Wang P, Chen Y. Downregulation of Meg3 enhances cisplatin resistance of lung cancer cells through activation of the WNT/beta-catenin signaling pathway. *Mol Med Rep.* 2015;12(3):4530-4537.

13. Xia H, Qu XL, Liu LY, Qian DH, Jing HY. LncRNA MEG3 promotes the sensitivity of vincristine by inhibiting autophagy in lung cancer chemotherapy. *Eur Rev Med Pharmacol Sci.* 2018;22(4):1020-1027.

14. Cao L, Chen J, Ou B, Liu C, Zou Y, chen Q. GAS5 knockdown reduces the chemo-sensitivity of non-small cell lung cancer (NSCLC) cell to cisplatin (DDP) through regulating miR-21/PTEN axis. *Biomedicine & Pharmacotherapy.* 2017;93:570-579.

15. Zhang N, Yang G, Shao X, Wei L. GAS5 modulated autophagy is a mechanism modulating cisplatin sensitivity in NSCLC cells. *Eur Rev Med Pharmacol Sci.* 2016;20(11):2271-2277.

16. Li L, Yin JY, He FZ, et al. Long noncoding RNA SFTA1P promoted apoptosis and increased cisplatin chemosensitivity via regulating the hnRNP-U-GADD45A axis in lung squamous cell carcinoma. *Oncotarget.* 2017;8(57):97476-97489.

17. Sun Y, Hu B, Wang Q, et al. Long non-coding RNA HOTTIP promotes BCL-2 expression and induces chemoresistance in small cell lung cancer by sponging miR-216a. *Cell Death Dis.* 2018;9(2):85.

18. Fang S, Gao H, Tong Y, et al. Long noncoding RNA-HOTAIR affects chemoresistance by regulating HOXA1 methylation in small cell lung cancer cells. *Lab Invest.* 2016;96(1):60-68.

19. Chen R, Chen B, Li D, et al. HOTAIR contributes to chemoresistance by activating NF-κB signaling in small-cell lung cancer. *International Journal of Clinical and Experimental Pathology.* 2019;12(8):2997.

20. Hu B, Zhang H, Wang Z, Zhang F, Wei H, Li L. LncRNA CCAT1/miR-130a-3p axis increases cisplatin resistance in non-small-cell lung cancer cell line by targeting SOX4. *Cancer Biol Ther.* 2017;18(12):974-983.

21. Chen J, Zhang K, Song H, Wang R, Chu X, Chen L. Long noncoding RNA CCAT1 acts as an oncogene and promotes chemoresistance in docetaxel-resistant lung adenocarcinoma cells. *Oncotarget.* 2016;7(38):62474-62489.

22. Wang L, Shang X, Feng Q. LncRNA TATDN1 contributes to the cisplatin resistance of non-small cell lung cancer through TATDN1/miR-451/TRIM66 axis. *Cancer Biol Ther.* 2019;20(3):261-271.

23. Huang FX, Chen HJ, Zheng FX, et al. LncRNA BLACAT1 is involved in chemoresistance of nonsmall cell lung cancer cells by regulating autophagy. *Int J Oncol.* 2019;54(1):339-347.

24. Niu Y, Ma F, Huang W, et al. Long non-coding RNA TUG1 is involved in cell growth and chemoresistance of small cell lung cancer by regulating LIMK2b via EZH2. *Mol Cancer.* 2017;16(1):5.

25. Guo S, Zhang L, Zhang Y, et al. Long non-coding RNA TUG1 enhances chemosensitivity in non-small cell lung cancer by impairing microRNA-221-dependent PTEN inhibition. *Aging (Albany NY).* 2019;11(18):7553-7569.

26. Wang Q, Cheng N, Li X, et al. Correlation of long non-coding RNA H19 expression with cisplatin-resistance and clinical outcome in lung adenocarcinoma. *Oncotarget.* 2017;8(2):2558-2567.

27. Ma L, Xie X, Ma L, et al. Downregulated long non-coding RNA TRPM2-AS inhibits cisplatin resistance of non-small cell lung cancer cells via activation of p53-p66shc pathway. *Eur Rev Med Pharmacol Sci.* 2017;21(11):2626-2634.

28. Ge P, Cao L, Yao YJ, Jing RJ, Wang W, Li HJ. lncRNA FOXD2-AS1 confers cisplatin resistance of non-small-cell lung cancer via regulation of miR185-5p-SIX1 axis. *Onco Targets Ther.* 2019;12:6105-6117.

29. Kumar P, Khadirnaikar S, Shukla SK. PILAR1, a novel prognostic LncRNA, reveals the presence of a unique subtype of lung adenocarcinoma patients with KEAP1 mutations. *Gene.* 2019;691:167-175.

30. Zeng F, Wang Q, Wang S, et al. Linc00173 promotes chemoresistance and progression of small cell lung cancer by sponging miR-218 to regulate Etk expression. *Oncogene.* 2020;39(2):293-307.

31. Gao BB, Wang SX. LncRNA BC200 regulates the cell proliferation and cisplatin resistance in non-small cell lung cancer via PI3K/AKT pathway. *Eur Rev Med Pharmacol Sci.* 2019;23(3):1093-1101.

32. Ren K, Xu R, Huang J, Zhao J, Shi W. Knockdown of long non-coding RNA KCNQ1OT1 depressed chemoresistance to paclitaxel in lung adenocarcinoma. *Cancer Chemother Pharmacol.* 2017;80(2):243-250.

33. Tang W, Yu X, Zeng R, Chen L. LncRNA-ATB Promotes Cisplatin Resistance in Lung Adenocarcinoma Cells by Targeting the miR-200a/beta-Catenin Pathway. *Cancer Manag Res.* 2020;12:2001-2014.

34. Xiao X, He S. ELF1 activated long non-coding RNA CASC2 inhibits cisplatin resistance of non-small cell lung cancer via the miR-18a/IRF-2 signaling pathway. *European Review for Medical and Pharmacological Sciences.* 2020;24(6):3130-3142.

35. Hu L, Chen SH, Lv QL, et al. Clinical Significance of Long Non-Coding RNA CASC8 rs10505477 Polymorphism in Lung Cancer Susceptibility, Platinum-Based Chemotherapy Response, and Toxicity. *Int J Environ Res Public Health.* 2016;13(6).

36. Xu R, Han Y. Long non-coding RNA FOXF1 adjacent non-coding developmental regulatory RNA inhibits growth and chemotherapy resistance in non-small cell lung cancer. *Arch Med Sci.* 2019;15(6):1539-1546.

37. Lin S, Zhang R, An X, et al. LncRNA HOXA-AS3 confers cisplatin resistance by interacting with HOXA3 in non-small-cell lung carcinoma cells. *Oncogenesis.* 2019;8(11):60.

38. Xiao H, Liu Y, Liang P, et al. TP53TG1 enhances cisplatin sensitivity of non-small cell lung cancer cells through regulating miR-18a/PTEN axis. *Cell & bioscience.* 2018;8(1):1-13.

39. Tang H, Han X, Li M, Li T, Hao Y. Linc00221 modulates cisplatin resistance in non-small-cell lung cancer via sponging miR-519a. *Biochimie.* 2019;162:134-143.

40. Zhao X, Li X, Zhou L, et al. LncRNA HOXA11-AS drives cisplatin resistance of human LUAD cells via modulating miR-454-3p/Stat3. *Cancer Sci.* 2018;109(10):3068-3079.

41. Yang Y, Li H, Hou S, Hu B, Liu J, Wang J. The noncoding RNA expression profile and the effect of lncRNA AK126698 on cisplatin resistance in non-small-cell lung cancer cell. *PLoS One.* 2013;8(5):e65309.

42. Xu YH, Tu JR, Zhao TT, Xie SG, Tang SB. Overexpression of lncRNA EGFRAS1 is associated with a poor prognosis and promotes chemotherapy resistance in nonsmall cell lung cancer. *Int J Oncol.* 2019;54(1):295-305.

43. Chen L, Han X, Hu Z, Chen L. The PVT1/miR-216b/Beclin-1 regulates cisplatin sensitivity of NSCLC cells via modulating autophagy and apoptosis. *Cancer Chemother Pharmacol.* 2019;83(5):921-931.

44. Zuo W, Zhang W, Xu F, Zhou J, Bai W. Long non-coding RNA LINC00485 acts as a microRNA-195 sponge to regulate the chemotherapy sensitivity of lung adenocarcinoma cells to cisplatin by regulating CHEK1. *Cancer Cell Int.* 2019;19:240.

45. Cai Y, Dong Z, Wang J. LncRNA NNT-AS1 is a major mediator of cisplatin chemoresistance in non-small cell lung cancer through MAPK/Slug pathway. *Eur Rev Med Pharmacol Sci.* 2018;22(15):4879-4887.

46. Wang W, Dong M, Zhang W, Liu T. Long noncoding LUCAT1 promotes cisplatin resistance of non-small cell lung cancer by promoting IGF-2. *Eur Rev Med Pharmacol Sci.* 2019;23(12):5229-5234.

47. Wang P, Chen D, Ma H, Li Y. LncRNA SNHG12 contributes to multidrug resistance through activating the MAPK/Slug pathway by sponging miR-181a in non-small cell lung cancer. *Oncotarget.* 2017;8(48):84086-84101.

48. Tian X, Gao S, Liu Y, Xuan Y, Wu R, Zhang Z. Long non-coding RNA ENST00000500843 is downregulated and promotes chemoresistance to paclitaxel in lung adenocarcinoma. *Oncol Lett.* 2019;18(4):3716-3722.

49. Zhang H, Luo Y, Xu W, Li K, Liao C. Silencing long intergenic non-coding RNA 00707 enhances cisplatin sensitivity in cisplatin-resistant non-small-cell lung cancer cells by sponging miR-145. *Oncol Lett.* 2019;18(6):6261-6268.

50. Pan Y, Chen J, Tao L, et al. Long noncoding RNA ROR regulates chemoresistance in docetaxel-resistant lung adenocarcinoma cells via epithelial mesenchymal transition pathway. *Oncotarget.* 2017;8(20):33144-33158.

51. Shi H, Pu J, Zhou XL, Ning YY, Bai C. Silencing long non-coding RNA ROR improves sensitivity of non-small-cell lung cancer to cisplatin resistance by inhibiting PI3K/Akt/mTOR signaling pathway. *Tumour Biol.* 2017;39(5):1010428317697568.

52. Li B, Gu W, Zhu X. NEAT1 mediates paclitaxel-resistance of non-small cell of lung cancer through activation of Akt/mTOR signalling pathway. *J Drug Target.* 2019;27(10):1061-1067.

53. Chen K, Abuduwufuer A, Zhang H, Luo L, Suotesiyali M, Zou Y. SNHG7 mediates cisplatin-resistance in non-small cell lung cancer by activating PI3K/AKT pathway. *Eur Rev Med Pharmacol Sci.* 2019;23(16):6935-6943.

54. Liu Y, Hu Q, Wang X. AFAP1-AS1 induces cisplatin resistance in non-small cell lung cancer through PI3K/AKT pathway. *Oncol Lett.* 2020;19(1):1024-1030.

55. Ye Y, Gu J, Liu P, et al. Long Non-Coding RNA SPRY4-IT1 Reverses Cisplatin Resistance by Downregulating MPZL-1 via Suppressing EMT in NSCLC. *Onco Targets Ther.* 2020;13:2783-2793.

56. Wu L, Liu C, Zhang Z. Knockdown of lncRNA MIAT inhibits proliferation and cisplatin resistance in non-small cell lung cancer cells by increasing miR-184 expression. *Oncol Lett.* 2020;19(1):533-541.

57. Li C, Fan K, Qu Y, et al. Deregulation of UCA1 expression may be involved in the development of chemoresistance to cisplatin in the treatment of non-small-cell lung cancer via regulating the signaling pathway of microRNA-495/NRF2. *J Cell Physiol.* 2020;235(4):3721-3730.

58. Zhang J, Xu C, Gao Y, et al. A Novel Long Non-coding RNA, MSTRG.51053.2 Regulates Cisplatin Resistance by Sponging the miR-432-5p in Non-small Cell Lung Cancer Cells. *Front Oncol.* 2020;10:215.
