# Supplementary material for: The function of LncRNAs and their role in the prediction, diagnosis, and prognosis of lung cancer
Source: Clin Transl Med. 2021 Apr 5;11(4):e367. doi: 10.1002/ctm2.367 (PMC8021541; doi:10.1002/ctm2.367)
Supplement: Supplementary file 3 — Table S3 [file CTM2-11-e367-s002.docx]

Supplementary Table S3. LncRNAs as predictive markers of radiosensitivity in lung cancer

| Official symbol | Ensemble accession no. | Genomic location | Description of the lncRNA | Types of lung cancer | Expression in lung cancer cells | Function in tumorigenesis | Mechanism of action | Radiotherapy | ﻿Dose rate | Effect on radiosensitivity in lung cancer | Reference |
| --- | --- | --- | --- | --- | --- | --- | --- | --- | --- | --- | --- |
| PVT1 | ENSG00000249859 | [Chromosome 8: 127,794,526-128,187,101](https://uswest.ensembl.org/Homo_sapiens/Location/View?db=core;g=ENSG00000249859;r=8:127794526-128187101) forward strand | Pvt1 oncogene | NSCLC | Upregulated | Oncogene | LncRNA PVT1 interacts with miR-195 and downregulates miR-195 | ﻿X-ray | 3.5 ﻿Gy/min | Upregulated lncRNA PVT1 reduces radiosensitivity | ^1^ |
|  |  |  |  |  |  |  | LncRNA PVT1 competitively binds miR-424-5p to downregulate CARM1 | X-ray | N/A |  | ^2^ |
| GAS5 | ENSG00000234741 | [Chromosome 1: 173,858,559-173,868,882](https://uswest.ensembl.org/Homo_sapiens/Location/View?db=core;g=ENSG00000234741;r=1:173858559-173868882) reverse strand | Growth arrest specific 5 | NSCLC | Downregulated | Tumor suppressor | LncRNA GAS5 interacts with miR-135b and negatively regulates the expression of miR-135b | X-ray | 3.2 Gy/min | Downregulated lncRNA GAS5 reduces radiosensitivity | ^3^ |
|  |  |  |  |  |  |  | Regulating the ﻿miR‑21/PTEN/Akt axis | X-ray | 1 Gy/min |  | ^4^ |
| GACAT3 | ENSG00000236289 | [Chromosome 2: 16,013,928-16,087,201](https://uswest.ensembl.org/Homo_sapiens/Location/View?db=core;g=ENSG00000236289;r=2:16013928-16087201) forward strand | Gastric cancer associated transcript 3 | NSCLC | Upregulated | Oncogene | LncRNA GACAT3 upregulates MMP10 by targeting TIMP2 | N/A | N/A | Overexpressed lncRNA GACAT3 enhances radiosensitivity | ^5^ |
| CYTOR | ENSG00000222041 | [Chromosome 2: 87,454,781-87,636,740](https://uswest.ensembl.org/Homo_sapiens/Location/View?db=core;g=ENSG00000222041;r=2:87454781-87636740) forward strand | Cytoskeleton regulator RNA; LINC00152 | NSCLC | Upregulated | Oncogene | CYTOR interacts with miR-195 and then upregulates the expression of its target genes | X-ray | 3.5 Gy/min | Upregulated lncRNA CYTOR reduces radiosensitivity | ^6^ |
| SBF2-AS1 | ENSG00000246273 | [Chromosome 11: 9,758,268-9,811,335](https://uswest.ensembl.org/Homo_sapiens/Location/View?db=core;g=ENSG00000246273;r=11:9758268-9811335) forward strand | SBF2 antisense RNA 1 | NSCLC | Upregulated | Oncogene | Regulating miR-302a/MBNL3 axis | X-ray | 2 Gy/min | Upregulated lncRNA SBF2-AS1 reduces radiosensitivity | ^7^ |
| ANKRD40CL | ENSG00000167117 | [Chromosome 17: 50,761,029-50,767,557](https://uswest.ensembl.org/Homo_sapiens/Location/View?db=core;g=ENSG00000167117;r=17:50761029-50767557) reverse strand | ANKRD40 C-terminal like; LINC00483 | LAD | Upregulated | Oncogene | Regulating miR‐144/HOXA10 axis | X-ray | 100 Mu/min | Upregulated ANKRD40CL reduces radiosensitivity | ^8^ |
| FAM201A | ENSG00000204860 | [Chromosome 9: 38,620,474-38,624,990](https://uswest.ensembl.org/Homo_sapiens/Location/View?db=core;g=ENSG00000204860;r=9:38620474-38624990) forward strand | Family with sequence similarity 201 member A | NSCLC | Upregulated | Oncogene | Regulating ﻿miR-370/EGFR axis | X-ray | N/A | Upregulated lncRNA FAM201A reduces radiosensitivity | ^9^ |
| LINC00461 | ENSG00000245526 | [Chromosome 5: 88,507,546-88,691,057](https://uswest.ensembl.org/Homo_sapiens/Location/View?db=core;g=ENSG00000245526;r=5:88507546-88691057) reverse strand | Long intergenic non-protein coding RNA 461 | LAD | Upregulated | Oncogene | Regulating ﻿miR-195/HOXA10 axis | X-ray | ﻿100 Mu/min | Upregulated LINC00461 reduces radiosensitivity | ^10^ |
| PPIAP43 | ENSG00000255059 | [Chromosome 11: 100,666,459-100,666,962](https://uswest.ensembl.org/Homo_sapiens/Location/View?db=core;g=ENSG00000255059;r=11:100666459-100666962;t=ENST00000527147) forward strand | Peptidylprolyl isomerase A pseudogene 43 | SCLC | Upregulated in cells irradiated | N/A | LncRNA transcribed from PPIAP43 interacts with microRNAs and regulates downstream target genes | ﻿Gamma | N/A | ﻿PPIAP43 RNA transcription enhances radiosensitivity | ^11^ |
| NEAT1 | ENSG00000245532 | [Chromosome 11: 65,422,774-65,445,540](https://uswest.ensembl.org/Homo_sapiens/Location/View?db=core;g=ENSG00000245532;r=11:65422774-65445540) forward strand | Nuclear paraspeckle assembly transcript 1 | NSCLC | Upregulated | Oncogene | Regulating miR-491-5p/CAPG axis | N/A | N/A | Upregulated lncRNA NEAT1  enhances radiosensitivity | ^12^ |
| CRNDE | ENSG00000245694 | [Chromosome 16: 54,845,189-54,929,189](https://uswest.ensembl.org/Homo_sapiens/Location/View?db=core;g=ENSG00000245694;r=16:54845189-54929189) reverse strand | Colorectal neoplasia differentially expressed | LAD | Upregulated | Oncogene | ﻿Regulating PRC2/p21 axis | X-ray | N/A | Upregulated lncRNA CRNDE reduces radiosensitivity | ^13^ |

Abbreviations

ANKRD40: Ankyrin Repeat Domain 40

CAPG: Capping Actin Protein, Gelsolin Like

CARM1: Coactivator Associated Arginine Methyltransferase 1

EGFR: Epidermal Growth Factor Receptor

Gy: Gray,a derived unit of ionizing radiation dose in the International System of Units

HOXA10: Homeobox A10

LAD: Lung adenocarcinoma

MBNL3: Muscleblind-Like Protein 3

MMP10: Matrix Metallopeptidase 10

Mu: Monitor unit

NSCLC: Non-small cell lung cancer

N/A: Not available

PRC2: Polycomb Repressive Complex 2

PTEN: Phosphatase And Tensin Homolog

PVT1: Plasmacytoma variant translocation 1

SBF2: SET Binding Factor 2

SCLC: Small cell lung cancer

TIMP2: Tissue Inhibitor Of Metalloproteinases 2

Supplementary References

1. Wu D, Li Y, Zhang H, Hu X. Knockdown of Lncrna PVT1 Enhances Radiosensitivity in Non-Small Cell Lung Cancer by Sponging Mir-195. *Cell Physiol Biochem.* 2017;42(6):2453-2466.

2. Wang D, Hu Y. Long Non-coding RNA PVT1 Competitively Binds MicroRNA-424-5p to Regulate CARM1 in Radiosensitivity of Non-Small-Cell Lung Cancer. *Mol Ther Nucleic Acids.* 2019;16:130-140.

3. Xue Y, Ni T, Jiang Y, Li Y. Long Noncoding RNA GAS5 Inhibits Tumorigenesis and Enhances Radiosensitivity by Suppressing miR-135b Expression in Non-Small Cell Lung Cancer. *Oncol Res.* 2017;25(8):1305-1316.

4. Chen L, Ren P, Zhang Y, Gong B, Yu D, Sun X. Long noncoding RNA GAS5 increases the radiosensitivity of A549 cells through interaction with the miR21/PTEN/Akt axis. *Oncol Rep.* 2020;43(3):897-907.

5. Yang X, Zhang W, Cheng S, Yang R. High expression of lncRNA GACAT3 inhibits invasion and metastasis of non-small cell lung cancer to enhance the effect of radiotherapy. *Eur Rev Med Pharmacol Sci.* 2018;22(5):1315-1322.

6. Zhang J, Li W. Long noncoding RNA CYTOR sponges miR-195 to modulate proliferation, migration, invasion and radiosensitivity in nonsmall cell lung cancer cells. *Biosci Rep.* 2018;38(6).

7. Yu Z, Wang G, Zhang C, et al. LncRNA SBF2-AS1 affects the radiosensitivity of non-small cell lung cancer via modulating microRNA-302a/MBNL3 axis. *Cell Cycle.* 2020;19(3):300-316.

8. Yang QS, Li B, Xu G, et al. Long noncoding RNA LINC00483/microRNA-144 regulates radiosensitivity and epithelial-mesenchymal transition in lung adenocarcinoma by interacting with HOXA10. *J Cell Physiol.* 2019;234(7):11805-11821.

9. Liu A, Zhu Y, Huang Z, Lei L, Fu S, Chen Y. Long noncoding RNA FAM201A involves in radioresistance of non-small-cell lung cancer by enhancing EGFR expression via miR-370. *Eur Rev Med Pharmacol Sci.* 2019;23(13):5802-5814.

10. Hou J, Wang Y, Zhang H, Hu Y, Xin X, Li X. Silencing of LINC00461 enhances radiosensitivity of lung adenocarcinoma cells by down-regulating HOXA10 via microRNA-195. *J Cell Mol Med.* 2020;24(5):2879-2890.

11. Wang S, Yu J. Long non-coding RNA transcribed from pseudogene PPIAP43 is associated with radiation sensitivity of small cell lung cancer cells. *Oncol Lett.* 2019;18(5):4583-4592.

12. Zhou S, Yang H. P1.03-34 The lncRNA NEAT1 Promotes Radioresistance via the MiR-491-5p/CAPG Axis in NSCLC. *Journal of Thoracic Oncology.* 2019;14(10):S431-S432.

13. Zhang M, Gao C, Yang Y, et al. Long Noncoding RNA CRNDE/PRC2 Participated in the Radiotherapy Resistance of Human Lung Adenocarcinoma Through Targeting p21 Expression. *Oncol Res.* 2018;26(8):1245-1255.
