# Supplementary material for: The function of LncRNAs and their role in the prediction, diagnosis, and prognosis of lung cancer
Source: Clin Transl Med. 2021 Apr 5;11(4):e367. doi: 10.1002/ctm2.367 (PMC8021541; doi:10.1002/ctm2.367)
Supplement: Supplementary file 4 — Table S4 [file CTM2-11-e367-s001.docx]

Supplementary Table S4. LncRNAs as predictive markers of ﻿sensitivity to EGFR-targeted therapy in lung cancer

| Official symbol | Ensemble/﻿GenBank accession no. | Genomic location | Description of the lncRNA | Types of lung cancer | Expression in drug-resistant cells of lung cancer | Function in tumorigenesis | Mechanism of action | EGFR-TKI(s) | Effect on sensitivity of targeted therapy in lung cancer | Reference |
| --- | --- | --- | --- | --- | --- | --- | --- | --- | --- | --- |
| SNHG12 | ENSG00000197989 | [Chromosome 1: 28,578,538-28,583,132](https://uswest.ensembl.org/Homo_sapiens/Location/View?db=core;g=ENSG00000197989;r=1:28578538-28583132) reverse strand | Small nucleolar RNA host gene 12 | NSCLC | Upregulated | Oncogene | Regulating miR-181a/MAPK/ Slug axis | Gefitinib | Upregulated lncRNA SNHG12 reduces the ﻿sensitivity to gefitinib | ^1^ |
| MIR31HG | ENSG00000171889 | [Chromosome 9: 21,453,802-21,559,900](https://uswest.ensembl.org/Homo_sapiens/Location/View?db=core;g=ENSG00000171889;r=9:21453802-21559900) reverse strand | MIR31 host gene; LOC554202 | NSCLC | Upregulated | Oncogene | Regulating ﻿EGFR/PI3K/AKT signaling pathway | ﻿Gefitinib | Upregulated lncRNA MIR31HG reduces  the ﻿sensitivity to gefitinib | ^2^ |
|  |  |  |  |  |  |  | ﻿Regulating RAF/MEK/ERK and  PI3K/AKT signaling pathways via modulating the expression of ﻿miR-31 |  |  | ^3^ |
| CASC9 | ENSG00000249395 | [Chromosome 8: 75,223,127-75,324,741](https://uswest.ensembl.org/Homo_sapiens/Location/View?db=core;g=ENSG00000249395;r=8:75223127-75324741) reverse strand | Cancer susceptibility 9 | NSCLC | Upregulated | Oncogene | Regulating cell growth, apoptosis and Chromatin assembly | Gefitinib | Upregulated lncRNA CASC9 reduces  the ﻿sensitivity to gefitinib | ^4^ |
| LINC00227 | N/A | N/A | EWAST1 | NSCLC | Downregulated | Tumor suppressor |  | Gefitinib | Downregulated LINC00227 reduces the ﻿sensitivity to gefitinib | ^4^ |
| H19 | ENSG00000130600 | [Chromosome 11: 1,995,176-2,001,470](https://uswest.ensembl.org/Homo_sapiens/Location/View?db=core;g=ENSG00000130600;r=11:1995176-2001470) reverse strand | H19 imprinted maternally expressed transcript | NSCLC | Upregulated | Oncogene | hnRNPA2B1-dependent H19 is packaged into exosomes, and then transferred to spread drug resistance to gefitinib | Gefitinib | Upregulated lncRNA H19 reduces the ﻿sensitivity to gefitinib | ^5^ |
|  |  |  |  | LAD | Upregulated | Oncogene | ﻿Regulating miR-148b-3p/ DDAH1 axis | Gefitinib |  | ^6^ |
|  |  |  |  | LC | Downregulated | Tumor suppressor | ﻿Upregulated PKM2 and phosphorylated AKT | ﻿Erlotinib | Downregulated lncRNA H19 reduces the ﻿sensitivity to erlotinib | ^7^ |
|  |  |  |  | NSCLC | Downregulated | Tumor suppressor | ﻿Regulating the levels of phosphorylated AKT and SRC | Erlotinib |  | ^8^ |
| UCA1 | ENSG00000214049 | [Chromosome 19: 15,828,206-15,836,328](https://uswest.ensembl.org/Homo_sapiens/Location/View?db=core;g=ENSG00000214049;r=19:15828206-15836328) forward strand | Urothelial cancer associated 1 | NSCLC | Upregulated | Oncogene | Inducing ﻿non-T790M mutation via activating the AKT/mTOR signaling pathway | ﻿Gefitinib | Upregulated lncRNA UCA1 reduces the ﻿sensitivity to gefitinib | ^9^ |
|  |  |  |  |  |  |  | LncRNA UCA1 interacts with EZH2 to reduce the expression of CDKN1A |  |  | ^10^ |
|  |  |  |  |  |  |  | Regulating ﻿miR-143/FOSL2 axis |  |  | ^11^ |
|  |  |  |  |  |  |  | Regulating STAT3 signaling pathway |  |  | ^12^ |
| BC087858 | BC087858 | Chromosome 6: 1,514,971-1,515,138 | A 1,322 bp intergenic lncRNAs and was found to be located near FOXC1 | NSCLC | Upregulated | Oncogene | Inducing ﻿non-T790M mutation via activating the PI3K/AKT and MEK/ERK signaling pathway and EMT | Gefitinib | Upregulated lncRNA BC087858 reduces  the ﻿sensitivity to gefitinib | ^13^ |
| HOTAIR | ENSG00000228630 | [Chromosome 12: 53,962,308-53,974,956](https://uswest.ensembl.org/Homo_sapiens/Location/View?db=core;g=ENSG00000228630;r=12:53962308-53974956) reverse strand | HOX transcript antisense RNA | LAD | Upregulated | Oncogene | Regulating TGF-α/EGFR signaling pathway and the expression of Bax/Caspase-3 | Gefitinib | Downregulated lncRNA HOTAIR enhances the ﻿sensitivity to gefitinib | ^14^ |
|  |  |  |  | NSCLC | Upregulated | Oncogene | ﻿Regulating the PDK1- and HOTAIR-mediated EZH2 | ATL-1 and erlotinib | Downregulated lncRNA HOTAIR enhances the ﻿sensitivity to ATL-1 and erlotinib | ^15^ |
| RHPN1-AS1 | ENSG00000254389 | [Chromosome 8: 143,366,631-143,368,548](https://uswest.ensembl.org/Homo_sapiens/Location/View?db=core;g=ENSG00000254389;r=8:143366631-143368548;t=ENST00000518049) reverse strand | RHPN1 antisense RNA 1 | NSCLC | Downregulated | Tumor suppressor | ﻿Regulating miR-299-3p/ TNFSF12 axis | Gefitinib | Downregulated lncRNA RHPN1-AS1  reduces the ﻿sensitivity to gefitinib | ^16^ |
| GAS5 | ENSG00000234741 | [Chromosome 1: 173,858,559-173,868,882](https://uswest.ensembl.org/Homo_sapiens/Location/View?db=core;g=ENSG00000234741;r=1:173858559-173868882) reverse strand | Growth arrest specific 5 | LAD | Downregulated | Tumor suppressor | ﻿Regulating EGFR signaling pathway and the expression of IGF-1R | Gefitinib | Downregulated lncRNA GAS5 reduces  the ﻿sensitivity to gefitinib | ^17^ |
| SNHG5 | ENSG00000203875 | [Chromosome 6: 85,650,491-85,678,932](https://uswest.ensembl.org/Homo_sapiens/Location/View?db=core;g=ENSG00000203875;r=6:85650491-85678932) reverse strand | Small nucleolar RNA host gene 5 | LAD | Downregulated | Tumor suppressor | Regulating miR-377/CASP1 axis | Gefitinib | Downregulated lncRNA SNHG5 reduces the ﻿sensitivity to gefitinib | ^18^ |
| SNHG14 | ENSG00000224078 | [Chromosome 15: 24,978,583-25,420,336](https://uswest.ensembl.org/Homo_sapiens/Location/View?db=core;g=ENSG00000224078;r=15:24978583-25420336) forward strand | Small nucleolar RNA host gene 14 | NSCLC | Upregulated | Oncogene | ﻿Regulating miR-206-3p/ABCB1 axis | Gefitinib | Upregulated lncRNA SNHG14 reduces the ﻿sensitivity to gefitinib | ^19^ |
| MALAT1 | ENSG00000251562 | [Chromosome 11: 65,497,688-65,506,516](https://uswest.ensembl.org/Homo_sapiens/Location/View?db=core;g=ENSG00000251562;r=11:65497688-65506516) forward strand | Metastasis associated lung adenocarcinoma transcript 1 | NSCLC | Upregulated | Oncogene | ﻿Regulating miR-200a/ZEB1 axis | Gefitinib | Upregulated lncRNA MALAT1 reduces the ﻿sensitivity to gefitinib | ^20^ |
| MIAT | ENSG00000225783 | [Chromosome 22: 26,646,411-26,676,475](https://uswest.ensembl.org/Homo_sapiens/Location/View?db=core;g=ENSG00000225783;r=22:26646411-26676475) forward strand | Myocardial infarction associated transcript | LC | Upregulated | Oncogene | ﻿Regulating PI3K/Akt signaling pathway via modulating miR-34a | Gefitinib | Upregulated lncRNA MIAT reduces the ﻿  sensitivity to gefitinib | ^21^ |
| HOST2 | ENSG00000285972 | [Chromosome 10: 84,167,228-84,172,093](https://uswest.ensembl.org/Homo_sapiens/Location/View?db=core;g=ENSG00000285972;r=10:84167228-84172093;t=ENST00000647830) reverse strand | Human Ovarian Cancer-Specific Transcript 2; CERNA2 | NSCLC | Upregulated | Oncogene | Regulating ﻿miRNA-621/SYF2 axis | Gefitinib | Upregulated lncRNA HOST2 reduces the ﻿sensitivity to gefitinib | ^22^ |
| LINC01116 | ENSG00000163364 | [Chromosome 2: 176,629,572-176,637,931](https://uswest.ensembl.org/Homo_sapiens/Location/View?db=core;g=ENSG00000163364;r=2:176629572-176637931) reverse strand | Long intergenic non-protein coding RNA 1116 | NSCLC | Upregulated | Oncogene | Regulating ﻿the expression of IFI44 | Gefitinib | Upregulated LINC01116 reduces the ﻿  sensitivity to gefitinib | ^23^ |
| LINC00460 | ENSG00000233532 | [Chromosome 13: 106,374,477-106,384,315](https://uswest.ensembl.org/Homo_sapiens/Location/View?db=core;g=ENSG00000233532;r=13:106374477-106384315) forward strand | Long intergenic non-protein coding RNA 460 | NSCLC | Upregulated | Oncogene | Regulating the expression of transcription factors FOS and NF-κB | Gefitinib, erlotinib, and osimertinib | Upregulated LINC00460 promotes the malignant process of EGFR-mutated LAD and resistance to EGFR-TKIs | ^24^ |
|  |  |  |  |  |  |  | ﻿Regulating miR-769-5p/EGFR axis | Gefitinib | Upregulated LINC00460 reduces the ﻿  sensitivity to gefitinib | ^25^ |
| LINC00665 | ENSG00000232677 | [Chromosome 19: 36,313,067-36,331,770](https://uswest.ensembl.org/Homo_sapiens/Location/View?db=core;g=ENSG00000232677;r=19:36313067-36331770) reverse strand | Long intergenic non-protein coding RNA 665 | NSCLC | Upregulated | Oncogene | ﻿Regulating EGFR signaling pathway | Gefitinib | Upregulated LINC00665 reduces the ﻿  sensitivity to gefitinib | ^26^ |
|  |  |  |  |  |  |  | LINC00665 interacts with EZH2 and regulates the PI3K/AKT signaling pathway |  |  | ^27^ |
| CCAT1 | 100507056 | Chromosome 8: 127,207,382-  127,219,268 Minus strand;  Chromosome8:  128,219,629-  128,231,333 Minus strand | Colon cancer-associated transcript-1; CARLo-5 | NSCLC | Upregulated | Oncogene | ﻿Regulating miR-218/﻿HOXA1 axis | Gefitinib | Upregulated lncRNA CCAT1 reduces the ﻿sensitivity to gefitinib | ^28^ |
| HAS2-AS1 | ENSG00000248690 | [Chromosome 8: 121,639,293-121,994,185](https://uswest.ensembl.org/Homo_sapiens/Location/View?db=core;g=ENSG00000248690;r=8:121639293-121994185) forward strand | HAS2 antisense RNA 1 | NSCLC | Upregulated | Oncogene | Regulating LSD1/EphB3 signaling pathway | Gefitinib | Upregulated lncRNA HAS2-AS 1 reduces the ﻿sensitivity to gefitinib | ^29^ |
| GAPLINC | ENSG00000266835 | [Chromosome 18: 3,466,250-3,478,978](https://uswest.ensembl.org/Homo_sapiens/Location/View?db=core;g=ENSG00000266835;r=18:3466250-3478978) forward strand | Gastric adenocarcinoma associated, positive CD44 regulator, long intergenic non-coding RNA; RP11-838N2.4 | NSCLC | Upregulated | Oncogene | ﻿FOXO1-dependent H19 is packaged into exosomes, and then transferred to spread drug resistance to erlotinib | ﻿Erlotinib | Upregulated lncRNA GAPLINC  reduces the ﻿sensitivity to erlotinib | ^30^ |
| BLACAT1 | ENSG00000281406 | [Chromosome 1: 205,434,885-205,457,091](https://uswest.ensembl.org/Homo_sapiens/Location/View?db=core;g=ENSG00000281406;r=1:205434885-205457091) reverse strand | Bladder cancer associated transcript 1 | NSCLC | Upregulated | Oncogene | Regulating STAT3 signaling pathway | Afatinib | Downregulated lncRNA BLACAT1 enhances the ﻿sensitivity to afatinib | ^31^ |
| ﻿MSTRG.292666.16 | N/A | N/A | Novel transcript | NSCLC | Upregulated | Oncogene | LncRNA MSTRG.292666.16 is packaged into exosomes, and then transferred to spread drug resistance to osimertinib | ﻿Osimertinib | Upregulated lncRNA MSTRG.292666.16 reduces the ﻿sensitivity to osimertinib | ^32^ |
| LINC00461 | ENSG00000245526 | [Chromosome 5: 88,507,546-88,691,057](https://uswest.ensembl.org/Homo_sapiens/Location/View?db=core;g=ENSG00000245526;r=5:88507546-88691057) reverse strand | Long intergenic non-protein coding RNA 461 | NSCLC | Upregulated | Oncogene | N/A | EGFR-TKI | Upregulated LINC00461 reduces the ﻿  sensitivity to EGFR-TKI | ^33^ |

Abbreviations

ABCB1: ATP Binding Cassette Subfamily B Member 1

ATL-1: Atractylenolide 1

CASP1: Caspase 1

CDKN1A: Cyclin Dependent Kinase Inhibitor 1A

DDAH1: Dimethylarginine Dimethylaminohydrolase 1

EGFR: Epidermal Growth Factor Receptor

﻿EGFR-TKI: EGFR Tyrosine Kinase Inhibitor

EMT: Epithelial–Mesenchymal Transition

EphB3: Eph Receptor B3

EZH2: Enhancer Of Zeste Homolog 2

FOSL2: FOS Like 2, AP-1 Transcription Factor Subunit

FOXC1: Forkhead Box C1

FOXO1: Forkhead Box O1

HAS2: Hyaluronan Synthase 2

HOXA1: Homeobox A1

IFI44: Interferon Induced Protein 44

IGF-1R: Insulin-like growth factor 1 receptor

LAD: Lung adenocarcinoma

LC: Lung cancer

LSD1: Lysine-Specifc Demethylase 1

MIR31: MicroRNA 31

mTOR: mammalian target of rapamycin

NSCLC: Non-small cell lung cancer

NF-κB: Nuclear factor kappa B

N/A: Not available

PDK1: Pyruvate Dehydrogenase Kinase 1

RHPN1: Rhophilin Rho GTPase Binding Protein 1

PI3K: Phosphoinositide 3-kinase

PKM2: Pyruvate Kinase Isozymes M2

STAT3: Signal Transducer and Activator of Transcription 3

TGF-α: Transforming growth factor alpha

TNFSF12: TNF Superfamily Member 12

ZEB1: Zinc Finger E-Box Binding Homeobox 1

Supplementary References

1. Wang P, Chen D, Ma H, Li Y. LncRNA SNHG12 contributes to multidrug resistance through activating the MAPK/Slug pathway by sponging miR-181a in non-small cell lung cancer. *Oncotarget.* 2017;8(48):84086-84101.

2. Wang B, Jiang H, Wang L, et al. Increased MIR31HG lncRNA expression increases gefitinib resistance in non-small cell lung cancer cell lines through the EGFR/PI3K/AKT signaling pathway. *Oncol Lett.* 2017;13(5):3494-3500.

3. He J, Jin S, Zhang W, et al. Long non-coding RNA LOC554202 promotes acquired gefitinib resistance in non-small cell lung cancer through upregulating miR-31 expression. *J Cancer.* 2019;10(24):6003-6013.

4. Ma P, Zhang M, Nie F, et al. Transcriptome analysis of EGFR tyrosine kinase inhibitors resistance associated long noncoding RNA in non-small cell lung cancer. *Biomed Pharmacother.* 2017;87:20-26.

5. Lei Y, Guo W, Chen B, Chen L, Gong J, Li W. Tumorreleased lncRNA H19 promotes gefitinib resistance via packaging into exosomes in nonsmall cell lung cancer. *Oncol Rep.* 2018;40(6):3438-3446.

6. Huang Z, Ma Y, Zhang P, Si J, Xiong Y, Yang Y. Long non-coding RNA H19 confers resistance to gefitinib via miR-148b-3p/DDAH1 axis in lung adenocarcinoma. *Anticancer Drugs.* 2020;31(1):44-54.

7. Chen C, Liu WR, Zhang B, et al. LncRNA H19 downregulation confers erlotinib resistance through upregulation of PKM2 and phosphorylation of AKT in EGFR-mutant lung cancers. *Cancer Lett.* 2020;486:58-70.

8. Li C, Zhang B. P1.03-46 LncRNA H19 Downregulation Promoted Resistance to EGFR-TKIs Through Regulating AKT and SRC Activating in NSCLC Cells. *Journal of Thoracic Oncology.* 2019;14(10).

9. Cheng N, Cai W, Ren S, et al. Long non-coding RNA UCA1 induces non-T790M acquired resistance to EGFR-TKIs by activating the AKT/mTOR pathway in EGFR-mutant non-small cell lung cancer. *Oncotarget.* 2015;6(27):23582-23593.

10. Xu T, Yan S, Wang M, et al. LncRNA UCA1 Induces Acquired Resistance to Gefitinib by Epigenetically Silencing CDKN1A Expression in Non-small-Cell Lung Cancer. *Front Oncol.* 2020;10:656.

11. Chen X, Wang Z, Tong F, Dong X, Wu G, Zhang R. lncRNA UCA1 Promotes Gefitinib Resistance as a ceRNA to Target FOSL2 by Sponging miR-143 in Non-small Cell Lung Cancer. *Mol Ther Nucleic Acids.* 2020;19:643-653.

12. Zhang B, Wang H, Wang Q, Xu J, Jiang P, Li W. Knockout of lncRNA UCA1 inhibits drug resistance to gefitinib via targeting STAT3 signaling in NSCLC. *Minerva Med.* 2019;110(3):273-275.

13. Pan H, Jiang T, Cheng N, et al. Long non-coding RNA BC087858 induces non-T790M mutation acquired resistance to EGFR-TKIs by activating PI3K/AKT and MEK/ERK pathways and EMT in non-small-cell lung cancer. *Oncotarget.* 2016;7(31):49948-49960.

14. Liu Y, Jiang H, Zhou H, et al. Lentivirus-mediated silencing of HOTAIR lncRNA restores gefitinib sensitivity by activating Bax/Caspase-3 and suppressing TGF-alpha/EGFR signaling in lung adenocarcinoma. *Oncol Lett.* 2018;15(3):2829-2838.

15. Xiao Q, Zheng F, Tang Q, et al. Repression of PDK1- and LncRNA HOTAIR-Mediated EZH2 Gene Expression Contributes to the Enhancement of Atractylenolide 1 and Erlotinib in the Inhibition of Human Lung Cancer Cells. *Cell Physiol Biochem.* 2018;49(4):1615-1632.

16. Li X, Zhang X, Yang C, Cui S, Shen Q, Xu S. The lncRNA RHPN1-AS1 downregulation promotes gefitinib resistance by targeting miR-299-3p/TNFSF12 pathway in NSCLC. *Cell Cycle.* 2018;17(14):1772-1783.

17. Dong S, Qu X, Li W, et al. The long non-coding RNA, GAS5, enhances gefitinib-induced cell death in innate EGFR tyrosine kinase inhibitor-resistant lung adenocarcinoma cells with wide-type EGFR via downregulation of the IGF-1R expression. *J Hematol Oncol.* 2015;8:43.

18. Wang Z, Pan L, Yu H, Wang Y. The long non-coding RNA SNHG5 regulates gefitinib resistance in lung adenocarcinoma cells by targetting miR-377/CASP1 axis. *Biosci Rep.* 2018;38(4).

19. Wu K, Li J, Qi Y, et al. SNHG14 confers gefitinib resistance in non-small cell lung cancer by up-regulating ABCB1 via sponging miR-206-3p. *Biomed Pharmacother.* 2019;116:108995.

20. Feng C, Zhao Y, Li Y, Zhang T, Ma Y, Liu Y. LncRNA MALAT1 Promotes Lung Cancer Proliferation and Gefitinib Resistance by Acting as a miR-200a Sponge. *Archivos de Bronconeumología (English Edition).* 2019;55(12):627-633.

21. Fu Y, Li C, Luo Y, Li L, Liu J, Gui R. Silencing of Long Non-coding RNA MIAT Sensitizes Lung Cancer Cells to Gefitinib by Epigenetically Regulating miR-34a. *Front Pharmacol.* 2018;9:82.

22. Chen Z, Liu H, Jiang N, Yuan J. LncRNA HOST2 enhances gefitinib-resistance in non-small cell lung cancer by down-regulating miRNA-621. *European Review for Medical and Pharmacological Sciences.* 2019;23(22):9939-9946.

23. Wang H, Lu B, Ren S, et al. Long Noncoding RNA LINC01116 Contributes to Gefitinib Resistance in Non-small Cell Lung Cancer through Regulating IFI44. *Mol Ther Nucleic Acids.* 2020;19:218-227.

24. Isobe K, Nakano Y, Kobayashi H, et al. Clinical importance of long noncoding RNA LINC00460 expression in plasma cell-free tumor RNA in EGFR-mutant adenocarcinoma. *Journal of Clinical Oncology.* 2019;37(15_suppl):e20529-e20529.

25. Ma G, Zhu J, Liu F, Yang Y. Long Noncoding RNA LINC00460 Promotes the Gefitinib Resistance of Nonsmall Cell Lung Cancer Through Epidermal Growth Factor Receptor by Sponging miR-769-5p. *DNA Cell Biol.* 2019;38(2):176-183.

26. Liu X, Lu X, Guo R. P2.13-34 Long Intergenic Non-Coding RNA 00665 Induces Acquired Resistance to Gefitinib in Non-Small-Cell Lung Cancer. *Journal of Thoracic Oncology.* 2018;13(10).

27. Liu X, Lu X, Zhen F, et al. LINC00665 Induces Acquired Resistance to Gefitinib through Recruiting EZH2 and Activating PI3K/AKT Pathway in NSCLC. *Mol Ther Nucleic Acids.* 2019;16:155-161.

28. Jin X, Liu X, Zhang Z, Guan Y. lncRNA CCAT1 Acts as a MicroRNA-218 Sponge to Increase Gefitinib Resistance in NSCLC by Targeting HOXA1. *Mol Ther Nucleic Acids.* 2020;19:1266-1275.

29. Sun P, Sun L, Cui J, Liu L, He Q. Long noncoding RNA HAS2-AS1 accelerates non-small cell lung cancer chemotherapy resistance by targeting LSD1/EphB3 pathway. *Am J Transl Res.* 2020;12(3):950-958.

30. Zhang W, Cai X, Yu J, Lu X, Qian Q, Qian W. Exosome-mediated transfer of lncRNA RP11838N2.4 promotes erlotinib resistance in non-small cell lung cancer. *Int J Oncol.* 2018;53(2):527-538.

31. Shu D, Xu Y, Chen W. Knockdown of lncRNA BLACAT1 reverses the resistance of afatinib to non-small cell lung cancer via modulating STAT3 signalling. *J Drug Target.* 2020;28(3):300-306.

32. Deng Q, Fang Q, Xie B, Sun H, Bao Y, Zhou S. Exosomal long non-coding RNA MSTRG.292666.16 is associated with osimertinib (AZD9291) resistance in non-small cell lung cancer. *Aging (Albany NY).* 2020;12(9):8001-8015.

33. Lee JY, Bach D-H, Hu R, Kim D, Lee SK. Role of long noncoding RNA LINC00461 in EGFR-TKIs resistant non-small cell lung cancer. In: AACR; 2019.
