# Supplementary material for: The function of LncRNAs and their role in the prediction, diagnosis, and prognosis of lung cancer
Source: Clin Transl Med. 2021 Apr 5;11(4):e367. doi: 10.1002/ctm2.367 (PMC8021541; doi:10.1002/ctm2.367)
Supplement: Supplementary file 5 — Table S5 [file CTM2-11-e367-s003.docx]

Supplementary Table S5. LncRNAs as diagnostic markers in lung cancer

| Official symbol | Ensemble accession no. | Genomic location | Description of the lncRNA | Types of lung cancer | Type of sample | Expression in lung cancer cells | Function in tumorigenesis | AUC (﻿Distinguishing patients from healthy controls) | Sensitivity | Specificity | Effect on lung cancer progression/diagnosis | Reference |
| --- | --- | --- | --- | --- | --- | --- | --- | --- | --- | --- | --- | --- |
| XLOC_009167 | N/A | N/A | Novel transcript | LC | ﻿Whole blood | Upregulated | Oncogene | 0.7398 | 78.7% | 61.8% | ﻿Distinguishing lung cancer from pneumonia | ^1^ |
| UCA1 | ENSG00000214049 | [Chromosome 19: 15,828,206-15,836,328](https://uswest.ensembl.org/Homo_sapiens/Location/View?db=core;g=ENSG00000214049;r=19:15828206-15836328) forward strand | Urothelial cancer associated 1 | NSCLC | Plasma | Upregulated | Oncogene | 0.886 | N/A | N/A | Promoting NSCLC malignant progression | ^2^ |
| CCAT2 | ENSG00000280997 | [Chromosome 8: 127,400,399-127,402,150](https://uswest.ensembl.org/Homo_sapiens/Location/View?db=core;g=ENSG00000280997;r=8:127400399-127402150;t=ENST00000630920) forward strand | Colon cancer associated transcript 2 | LAD | Tissues and cells | Upregulated | Oncogene | 0.589 | N/A | N/A | Promoting invasion of LAD; Predicting lymph node metastasis | ^3^ |
| GAS5 | ENSG00000234741 | [Chromosome 1: 173,858,559-173,868,882](https://uswest.ensembl.org/Homo_sapiens/Location/View?db=core;g=ENSG00000234741;r=1:173858559-173868882) reverse strand | Growth arrest specific 5 | NSCLC | ﻿Serum | Downregulated | Tumor suppressor | 0.857 | 85.94% | 70% | ﻿GAS5 in exosomes can be used to identified patients with early-stage NSCLC | ^4^ |
| AFAP1-AS1 | ENSG00000272620 | [Chromosome 4: 7,754,077-7,778,928](https://uswest.ensembl.org/Homo_sapiens/Location/View?db=core;g=ENSG00000272620;r=4:7754077-7778928) forward strand | AFAP1 antisense RNA 1 | NSCLC | Serum | Upregulated | Oncogene | 0.759 | 69.3% | 88.3% | Promoting distant metastasis and lymph node metastasis of NSCLC | ^5^ |
| CYTOR | ENSG00000222041 | [Chromosome 2: 87,454,781-87,636,740](https://uswest.ensembl.org/Homo_sapiens/Location/View?db=core;g=ENSG00000222041;r=2:87454781-87636740) forward strand | Cytoskeleton regulator RNA;﻿LINC00152 | NSCLC | Serum | Upregulated | Oncogene | 0.816 | 80% | 72% | ﻿Distinguish NSCLC patients from benign lung disease | ^6^ |
| HOTAIR | ENSG00000228630 | [Chromosome 12: 53,962,308-53,974,956](https://uswest.ensembl.org/Homo_sapiens/Location/View?db=core;g=ENSG00000228630;r=12:53962308-53974956) reverse strand | HOX transcript antisense RNA | NSCLC | Plasma | Upregulated | Oncogene | 0.806 | 76.2% | 71.9% | Promoting NSCLC cells invasion and metastasis | ^7^ |
| MALAT1 | ENSG00000251562 | [Chromosome 11: 65,497,688-65,506,516](https://uswest.ensembl.org/Homo_sapiens/Location/View?db=core;g=ENSG00000251562;r=11:65497688-65506516) forward strand | Metastasis associated lung adenocarcinoma transcript 1 | NSCLC | blood | Upregulated | Oncogene | 0.79 | 56% | 96% | When it used as a single molecular marker for diagnosis of NSCLC, the sensitivity is low and can be improved as a complementary biomarker within a panel | ^8^ |
| ﻿SNHG1-210 | ﻿ENST00000539303 | [Chromosome 11: 62,853,573-62,855,174](http://uswest.ensembl.org/Homo_sapiens/Location/View?db=core;g=ENSG00000255717;r=11:62853573-62855174;t=ENST00000539303) reverse strand | Small nucleolar RNA host gene 1;LncRNA16 | LC | Tissues, plasma and cells | Upregulated | Oncogene | 0.858 | 73.97% | 100% | Promoting the proliferation of lung cancer cells by regulating cell cycle | ^9^ |
| TUG1 | ENSG00000253352 | [Chromosome 22: 30,969,245-30,979,395](http://uswest.ensembl.org/Homo_sapiens/Location/View?db=core;g=ENSG00000253352;r=22:30969245-30979395) forward strand | Taurine up-regulated 1 | LAD | Serum and cells | Upregulated | Oncogene | 0.756 | 78.33% | 60% | Physical interaction with EZH2 inhibits BAX expression, thereby inhibiting apoptosis | ^10^ |
| SOX2OT | ENSG00000242808 | [Chromosome 3: 180,989,762-181,836,880](http://uswest.ensembl.org/Homo_sapiens/Location/View?db=core;g=ENSG00000242808;r=3:180989762-181836880) forward strand | SOX2 overlapping transcript | LSCC | Plasma | Upregulated | Oncogene | 0.815 | 76% | 73.17% | Promoting LSCC tumor enlargement and lymph node metastasis | ^11^ |
| TBILA | ENSG00000261488 | [Chromosome 3: 112,133,423-112,135,359](http://uswest.ensembl.org/Homo_sapiens/Location/View?db=core;g=ENSG00000261488;r=3:112133423-112135359;t=ENST00000563632) forward strand | TGF-beta induced lncRNA | NSCLC | ﻿Serum | Upregulated | Oncogene | 0.923 | 64.7% | 80.7% | Significantly correlated with tumor size | ^12^ |
| AGAP2-AS1 | ENSG00000255737 | [Chromosome 12: 57,726,271-57,728,356](http://uswest.ensembl.org/Homo_sapiens/Location/View?db=core;g=ENSG00000255737;r=12:57726271-57728356;t=ENST00000542466) forward strand | AGAP2 antisense RNA 1 | NSCLC | ﻿Serum | Upregulated | Oncogene | 0.846 | N/A | N/A | ﻿Significantly correlated with lymph node metastasis and ﻿tumor stage | ^13^ |
| MAGI2-AS3 | ENSG00000234456 | [Chromosome 7: 79,452,877-79,471,208](https://uswest.ensembl.org/Homo_sapiens/Location/View?db=core;g=ENSG00000234456;r=7:79452877-79471208) forward strand | MAGI2 antisense RNA 3 | LAD | ﻿Plasma and Platelets | Downregulated | Tumor suppressor | 0.853 in Plasma  0.866 in Platelets | 82.2%/88.2%/  100% ^†^ | 98.5%/92.7%/  92% ^†^ | ﻿MAGI2-AS3 and ZFAS1 in tumor-educated blood platelets can be used to diagnose LSCC and LAD | ^14^ |
|  |  |  |  | LSCC | Plasma and Platelets |  |  | 0.892 in Plasma  0.887 in Platelets | 100%/98.4% ^‡^ | 87.7%/92.9% ^‡^ |  |  |
| ZFAS1 | ENSG00000177410 | [Chromosome 20: 49,278,178-49,299,600](https://uswest.ensembl.org/Homo_sapiens/Location/View?db=core;g=ENSG00000177410;r=20:49278178-49299600) forward strand | ZNFX1 antisense RNA 1 | LAD | ﻿Plasma and Platelets | Downregulated | Tumor suppressor | 0.780 in Plasma  0.806 in Platelets | 64.4%/61.2%/  92%^†^ | 64.6%/78.6%/  68%^†^ |  |  |
|  |  |  |  | LSCC | Plasma and Platelets |  |  | 0.744 in Plasma  0.770 in Platelets | 63%/57.4% ^‡^ | 69.2%/78.6% ^‡^ |  |  |
| PCAT6 | ENSG00000228288 | [Chromosome 1: 202,810,954-202,812,156](https://uswest.ensembl.org/Homo_sapiens/Location/View?db=core;g=ENSG00000228288;r=1:202810954-202812156) forward strand | Prostate cancer associated transcript 6 | LAD | Tissues and plasma | Upregulated | Oncogene | 0.9213 | 87.67% | 97.44% | Promoting the proliferation and invasion of LAD cells | ^15^ |
|  |  |  |  | LSCC |  |  |  | 0.9583 | 94.12 | 100% |  |  |
| PRAL | ENSG00000279296 | [Chromosome 17: 6,772,831-6,776,116](http://uswest.ensembl.org/Homo_sapiens/Location/View?db=core;g=ENSG00000279296;r=17:6772831-6776116;t=ENST00000624952) reverse strand | p53 regulation associated lncRNA | NSCLC | Tissues and cells | Downregulated | Tumor suppressor | 0.8546 | N/A | N/A | Inhibiting the proliferation, invasion and metastasis of NSCLC cells | ^16^ |
| ﻿CDKN2B-AS1 | ENSG00000240498 | [Chromosome 9: 21,994,139-22,128,103](https://uswest.ensembl.org/Homo_sapiens/Location/View?db=core;g=ENSG00000240498;r=9:21994139-22128103) forward strand | CDKN2B antisense RNA 1; ANRIL | NSCLC | Tissues and serum | Upregulated | Oncogene | 0.723 | N/A | N/A | Promoting NSCLC malignant progression | ^17^ |
| PVT1 | ENSG00000249859 | [Chromosome 8: 127,794,526-128,187,101](https://uswest.ensembl.org/Homo_sapiens/Location/View?db=core;g=ENSG00000249859;r=8:127794526-128187101) forward strand | Pvt1 oncogene | NSCLC | Tissues and cells | Upregulated | Oncogene | 0.736 | 81.5% | 61.7% | ﻿Promoting cell proliferation *via* downregulating the expression of *p15* and *p21* | ^18^ |
| ﻿NEAT1 | ENSG00000245532 | [Chromosome 11: 65,422,774-65,445,540](https://uswest.ensembl.org/Homo_sapiens/Location/View?db=core;g=ENSG00000245532;r=11:65422774-65445540) forward strand | Nuclear paraspeckle assembly transcript 1 | NSCLC | Plasma | Upregulated | Oncogene | 0.72 | 81% | 54% | Promoting NSCLC growth | ^19^ |
| DLX6-AS1 | ENSG00000231764 | [Chromosome 7: 96,955,141-97,014,088](https://uswest.ensembl.org/Homo_sapiens/Location/View?db=core;g=ENSG00000231764;r=7:96955141-97014088) reverse strand | DLX6 antisense RNA 1 | NSCLC | Tissues and serum | Upregulated | Oncogene | 0.806 | 77.5% | 85.9% | Promoting NSCLC cell proliferation and migration | ^20^ |

†. The data are from three GEO datasets. ‡. The data are from two GEO datasets.

Abbreviations

AFAP1: Actin Filament Associated Protein 1

AGAP2: ArfGAP With GTPase Domain, Ankyrin Repeat And PH Domain 2

AUC: Are under the ROC curve

CDKN2B: Cyclin Dependent Kinase Inhibitor 2B

DLX6: Distal-Less Homeobox 6

LAD: Lung adenocarcinoma

LC: Lung cancer

LSCC: ﻿Lung Squamous Cell Carcinoma

MAGI2: Membrane Associated Guanylate Kinase, WW And PDZ Domain Containing 2

NSCLC: ﻿Non-small cell lung cancer

N/A:﻿ Not available

PVT1: Plasmacytoma variant translocation 1

﻿ROC: Receiver operating characteristic curve

SOX2: SRY-Box Transcription Factor 2

TGF: Transforming growth factor

ZNFX1: Zinc Finger NFX1-Type Containing 1

﻿

Supplementary References

1. Jiang N, Meng X, Mi H, et al. Circulating lncRNA XLOC_009167 serves as a diagnostic biomarker to predict lung cancer. *Clin Chim Acta.* 2018;486:26-33.

2. Wang H-M, Lu J-H, Chen W-Y, Gu A-Q. Upregulated lncRNA-UCA1 contributes to progression of lung cancer and is closely related to clinical diagnosis as a predictive biomarker in plasma. *International journal of clinical and experimental medicine.* 2015;8(7):11824.

3. Qiu M, Xu Y, Yang X, et al. CCAT2 is a lung adenocarcinoma-specific long non-coding RNA and promotes invasion of non-small cell lung cancer. *Tumour Biol.* 2014;35(6):5375-5380.

4. Li C, Lv Y, Shao C, et al. Tumor-derived exosomal lncRNA GAS5 as a biomarker for early-stage non-small-cell lung cancer diagnosis. *J Cell Physiol.* 2019;234(11):20721-20727.

5. Li W, Li N, Kang X, Shi K. Circulating long non-coding RNA AFAP1-AS1 is a potential diagnostic biomarker for non-small cell lung cancer. *Clin Chim Acta.* 2017;475:152-156.

6. Li N, Feng XB, Tan Q, et al. Identification of Circulating Long Noncoding RNA Linc00152 as a Novel Biomarker for Diagnosis and Monitoring of Non-Small-Cell Lung Cancer. *Dis Markers.* 2017;2017:7439698.

7. Li N, Wang Y, Liu X, et al. Identification of Circulating Long Noncoding RNA HOTAIR as a Novel Biomarker for Diagnosis and Monitoring of Non-Small Cell Lung Cancer. *Technol Cancer Res Treat.* 2017;16(6):1060-1066.

8. Weber DG, Johnen G, Casjens S, et al. Evaluation of long noncoding RNA MALAT1 as a candidate blood-based biomarker for the diagnosis of non-small cell lung cancer. *BMC Res Notes.* 2013;6:518.

9. Zhu H, Zhang L, Yan S, et al. LncRNA16 is a potential biomarker for diagnosis of early-stage lung cancer that promotes cell proliferation by regulating the cell cycle. *Oncotarget.* 2017;8(5):7867-7877.

10. Liu H, Zhou G, Fu X, et al. Long noncoding RNA TUG1 is a diagnostic factor in lung adenocarcinoma and suppresses apoptosis via epigenetic silencing of BAX. *Oncotarget.* 2017;8(60):101899-101910.

11. Teng Y, Kang H, Chu Y. Identification of an Exosomal Long Noncoding RNA SOX2-OT in Plasma as a Promising Biomarker for Lung Squamous Cell Carcinoma. *Genet Test Mol Biomarkers.* 2019;23(4):235-240.

12. Tao Y, Tang Y, Yang Z, et al. Exploration of Serum Exosomal LncRNA TBILA and AGAP2-AS1 as Promising Biomarkers for Diagnosis of Non-Small Cell Lung Cancer. *Int J Biol Sci.* 2020;16(3):471-482.

13. Fan K, Liu Y, Yang B, Tian X, Li C, Wang B. Prognostic and diagnostic significance of long non-coding RNA AGAP2-AS1 levels in patients with non-small cell lung cancer. *Eur Rev Med Pharmacol Sci.* 2017;21(10):2392-2396.

14. Luo CL, Xu ZG, Chen H, et al. LncRNAs and EGFRvIII sequestered in TEPs enable blood-based NSCLC diagnosis. *Cancer Manag Res.* 2018;10:1449-1459.

15. Wan L, Zhang L, Fan K, Wang JJ. Diagnostic significance of circulating long noncoding RNA PCAT6 in patients with non-small cell lung cancer. *Onco Targets Ther.* 2017;10:5695-5702.

16. Wang H, Wang J, Liang CF, Zhou T. Expression of Long Non-Coding RNA PRAL as a Potential Biomarker for Diagnosis in Non-Small-Cell Lung Cancer Patients is Associated with the Inhibition of Cell Proliferation and Metastasis. *Clin Lab.* 2018;64(9):1341-1348.

17. Xie Y, Zhang Y, Du L, et al. Circulating long noncoding RNA act as potential novel biomarkers for diagnosis and prognosis of non-small cell lung cancer. *Mol Oncol.* 2018;12(5):648-658.

18. Cui D, Yu CH, Liu M, Xia QQ, Zhang YF, Jiang WL. Long non-coding RNA PVT1 as a novel biomarker for diagnosis and prognosis of non-small cell lung cancer. *Tumour Biol.* 2016;37(3):4127-4134.

19. Guo X, Xiang Y, Yuan S, et al. Expression and diagnostic significance of lncRNA NEAT1 in peripheral blood of patients with non-small cell lung cancer. *Journal of Third Military Medical University.* 2019;41(05):448-453.

20. Zhang X, Guo H, Bao Y, Yu H, Xie D, Wang X. Exosomal long non-coding RNA DLX6-AS1 as a potential diagnostic biomarker for non-small cell lung cancer. *Oncol Lett.* 2019;18(5):5197-5204.
