# Supplementary material for: The function of LncRNAs and their role in the prediction, diagnosis, and prognosis of lung cancer
Source: Clin Transl Med. 2021 Apr 5;11(4):e367. doi: 10.1002/ctm2.367 (PMC8021541; doi:10.1002/ctm2.367)
Supplement: Supplementary file 6 — Table S6 [file CTM2-11-e367-s004.docx]

Supplementary Table S6. LncRNAs as prognostic markers in lung cancer

| Official symbol | Ensemble/﻿GenBank accession no. | Genomic location | Description of the lncRNA | Types of lung cancer | Expression in lung cancer cells | Function in tumorigenesis | The HR of lncRNA expression | | Tendency of prognosis (if the lncRNA is upregulated) | Effect on lung cancer progression | Reference |
| --- | --- | --- | --- | --- | --- | --- | --- | --- | --- | --- | --- |
|  |  |  |  |  |  |  | Univariate analysis | Multivariate analysis |  |  |  |
| EGFR‑AS1 | ENSG00000224057 | [Chromosome 7: 55,179,750-55,188,934](https://uswest.ensembl.org/Homo_sapiens/Location/View?db=core;g=ENSG00000224057;r=7:55179750-55188934;t=ENST00000442411) reverse strand | EGFR Antisense RNA 1 | NSCLC | Upregulated | Oncogene | N/A | 1.542 | Poor | ﻿Promoting NSCLC cell proliferation and  ﻿chemoresistance *via* regulating ﻿miR-223/﻿IGF1R axis | ^1^ |
| MIAT | ENSG00000225783 | [Chromosome 22: 26,646,411-26,676,475](https://uswest.ensembl.org/Homo_sapiens/Location/View?db=core;g=ENSG00000225783;r=22:26646411-26676475) forward strand | Myocardial infarction associated transcript | LC | Upregulated | Oncogene | 2.74 | 3.53 | Poor | Upregulated lncRNA MIAT promotes resistance of lung cancer cells to gefitinib | ^2^ |
| AGAP2-AS1 | ENSG00000255737 | [Chromosome 12: 57,726,271-57,728,356](https://uswest.ensembl.org/Homo_sapiens/Location/View?db=core;g=ENSG00000255737;r=12:57726271-57728356;t=ENST00000542466) forward strand | AGAP2 antisense RNA 1 | NSCLC | Upregulated | Oncogene | 2.659 | 2.139 | Poor | Promoting the malignant progression of NSCLC | ^3^ |
| SLC16A1-AS1 | ENSG00000226419 | [Chromosome 1: 112,956,415-113,047,055](https://uswest.ensembl.org/Homo_sapiens/Location/View?db=core;g=ENSG00000226419;r=1:112956415-113047055) forward strand | SLC16A1 antisense RNA 1 | NSCLC | Downregulated | Tumor suppressor | 3.858 | 3.351 | Good | ﻿Regulating ﻿the viability and proliferation of NSCLC cells *via* modulating the phosphorylation level of Ras/Raf/MEK/ERK signaling pathway | ^4^ |
| PVT1 | ENSG00000249859 | [Chromosome 8: 127,794,526-128,187,101](https://uswest.ensembl.org/Homo_sapiens/Location/View?db=core;g=ENSG00000249859;r=8:127794526-128187101) forward strand | Pvt1 oncogene | NSCLC | Upregulated | Oncogene | 2.634 | 1.782 | Poor | Promoting lung cancer metastasis and invasion | ^5^ |
| MIR31HG | ENSG00000171889 | [Chromosome 9: 21,453,802-21,559,900](https://uswest.ensembl.org/Homo_sapiens/Location/View?db=core;g=ENSG00000171889;r=9:21453802-21559900) reverse strand | MIR31 host gene | LAD | Upregulated | Oncogene | 2.4080 | 1.734 | Poor | Regulating LAD cells proliferation and cell cycle | ^6^ |
| HOTAIR | ENSG00000228630 | [Chromosome 12: 53,962,308-53,974,956](https://uswest.ensembl.org/Homo_sapiens/Location/View?db=core;g=ENSG00000228630;r=12:53962308-53974956) reverse strand | HOX transcript antisense RNA | NSCLC | Upregulated | Oncogene | N/A | N/A | Poor | ﻿Regulating NSCLC cell invasion and metastasis, partially through the down-modulating HOXA5 expression | ^7^ |
| GACAT2 | ENSG00000265962 | [Chromosome 18: 8,695,856-8,707,621](https://uswest.ensembl.org/Homo_sapiens/Location/View?db=core;g=ENSG00000265962;r=18:8695856-8707621;t=ENST00000579368) reverse strand | Gastric cancer associated transcript 2;HMlincRNA717 | NSCLC | Downregulated | Tumor suppressor | 2.885 | 2.473 | Good | ﻿Regulating the malignant progression of NSCLC | ^8^ |
| ﻿MVIH | N/A | N/A | ﻿LncRNA associated with microvascular  invasion in HCC; located within the *RPS24* gene | NSCLC | Upregulated | Oncogene | N/A | N/A | Poor | ﻿Promoting NSCLC cell proliferation and invasion, partially through regulating MMP2 and MMP9 expression | ^9^ |
| CDKN2B-AS1 | ENSG00000240498 | [Chromosome 9: 21,994,139-22,128,103](https://uswest.ensembl.org/Homo_sapiens/Location/View?db=core;g=ENSG00000240498;r=9:21994139-22128103) forward strand | CDKN2B antisense RNA 1; ANRIL | NSCLC | Upregulated | Oncogene | 2.793 | 2.538 | Poor | Promoting the growth and metastasis of NSCLC cells | ^10^ |
| PANDAR | ENSG00000281450 | [Chromosome 6: 36,673,621-36,675,126](https://uswest.ensembl.org/Homo_sapiens/Location/View?db=core;g=ENSG00000281450;r=6:36673621-36675126;t=ENST00000629595) reverse strand | Promoter of CDKN1A antisense DNA damage activated RNA | NSCLC | Downregulated | Tumor suppressor | 0.557 | 0.652 | Good | Regulating the apoptosis of NSCLC cells through ﻿NF-YA/Bcl-2 axis | ^11^ |
| ﻿SBF2-AS1 | ENSG00000246273 | [Chromosome 11: 9,758,268-9,811,335](https://uswest.ensembl.org/Homo_sapiens/Location/View?db=core;g=ENSG00000246273;r=11:9758268-9811335) forward strand | SBF2 antisense RNA 1 | NSCLC | Upregulated | Oncogene | 2.464 | 2.341 | Poor | ﻿Promoting the malignant progression of NSCLC | ^12^ |
| CCAT1 | 100507056 | ﻿Chromosome 8: 127,207,382-  127,219,268 Minus strand;  Chromosome8:  128,219,629-128,231,333 Minus strand | Colon cancer associated transcript 1; CARLo-5 | NSCLC | Upregulated | Oncogene | N/A | N/A | Poor | Promoting ﻿proliferation, migration, and invasion in NSCLC | ^13^ |
| CCAT2 | ENSG00000280997 | [Chromosome 8: 127,400,399-127,402,150](https://uswest.ensembl.org/Homo_sapiens/Location/View?db=core;g=ENSG00000280997;r=8:127400399-127402150;t=ENST00000630920) forward strand | Colon cancer associated transcript 2 | SCLC | Upregulated | Oncogene | 2.676 | 2.034 | Poor | Promoting SCLC cells metastasis | ^14^ |
| TUBA4B | ENSG00000243910 | [Chromosome 2: 219,253,243-219,272,197](https://uswest.ensembl.org/Homo_sapiens/Location/View?db=core;g=ENSG00000243910;r=2:219253243-219272197) forward strand | Tubulin alpha 4b | NSCLC | Downregulated | Tumor suppressor | N/A | N/A | Good | Regulating NSCLC cells proliferation | ^15^ |
| GHET1 | ENSG00000281189 | [Chromosome 7: 148,987,527-148,989,432](https://uswest.ensembl.org/Homo_sapiens/Location/View?db=core;g=ENSG00000281189;r=7:148987527-148989432;t=ENST00000627071) forward strand | Gastric carcinoma proliferation enhancing transcript 1 | NSCLC | Upregulated | Oncogene | N/A | N/A | Poor | ﻿Promoting the malignant progression of NSCLC | ^16^ |
| NMRAL2P | ENSG00000171658 | [Chromosome 3: 185,959,943-185,980,872](https://uswest.ensembl.org/Homo_sapiens/Location/View?db=core;g=ENSG00000171658;r=3:185959943-185980872) forward strand | NmrA like redox sensor 2; LOC344887 | NSCLC | Upregulated | Oncogene | 4.317 | 3.783 | Poor | ﻿Promoting the malignant progression of NSCLC | ^17^ |
| BCAR4 | ENSG00000262117 | [Chromosome 16: 11,819,829-11,828,845](https://uswest.ensembl.org/Homo_sapiens/Location/View?db=core;g=ENSG00000262117;r=16:11819829-11828845) reverse strand | Breast cancer anti-estrogen resistance 4 | NSCLC | Upregulated | Oncogene | 2.853 | 2.643 | Poor | ﻿Promoting the malignant progression of NSCLC | ^18^ |
| LL22NC03-N64E9.1 | ENSG00000271127 | ﻿[Chromosome 22: 16,179,617-16,181,004](http://grch37.ensembl.org/Homo_sapiens/Location/View?db=core;g=ENSG00000271127;r=22:16179617-16181004;t=ENST00000603308) reverse strand | Novel ﻿transcript; Sense intronic | LC | Upregulated | Oncogene | N/A | N/A | Poor | ﻿Promoting proliferation of lung cancer cells | ^19^ |
| LINC-ROR | ENSG00000258609 | [Chromosome 18: 57,054,558-57,072,119](https://uswest.ensembl.org/Homo_sapiens/Location/View?db=core;g=ENSG00000258609;r=18:57054558-57072119) reverse strand | Long intergenic non-protein coding RNA, regulator of reprogramming | NSCLC | Upregulated | Oncogene | N/A | 2.983 | Poor | Promoting the malignant progression of NSCLC | ^20^ |
| PART1 | ENSG00000152931 | [Chromosome 5: 60,487,713-60,548,813](https://uswest.ensembl.org/Homo_sapiens/Location/View?db=core;g=ENSG00000152931;r=5:60487713-60548813) forward strand | Prostate androgen-regulated transcript 1 | NSCLC | Upregulated | Oncogene | 1.80 | 2.11 | Poor | Representing NSCLC recurrence in stage I–III | ^21^ |
| AFAP1-AS1 | ENSG00000272620 | [Chromosome 4: 7,754,077-7,778,928](https://uswest.ensembl.org/Homo_sapiens/Location/View?db=core;g=ENSG00000272620;r=4:7754077-7778928) forward strand | AFAP1 antisense RNA 1 | NSCLC | Upregulated | Oncogene | 1.057 | 1.046 | Poor | Regulating NSCLC cell proliferation by epigenetically inhibiting *p21* expression | ^22^ |
| CTB-193M12.5 | N/A | N/A | Novel ﻿transcript | LAD | Upregulated | Oncogene | N/A | N/A | Poor | N/A | ^23^ |
| BANCR | ENSG00000278910 | [Chromosome 9: 69,296,682-69,311,111](https://uswest.ensembl.org/Homo_sapiens/Location/View?db=core;g=ENSG00000278910;r=9:69296682-69311111) reverse strand | BRAF-activated non-protein coding RNA | NSCLC | Downregulated | Tumor suppressor | 0.367 | 0.496 | Good | Regulating lung cancer metastasis by affecting EMT | ^24^ |
| CASC2 | ENSG00000177640 | [Chromosome 10: 118,046,279-118,210,158](https://uswest.ensembl.org/Homo_sapiens/Location/View?db=core;g=ENSG00000177640;r=10:118046279-118210158) forward strand | Cancer susceptibility 2 | NSCLC | Downregulated | Tumor suppressor | 0.192 | 0.276 | Good | ﻿Regulating proliferation in NSCLC cells | ^25^ |
| H19 | ENSG00000130600 | [Chromosome 11: 1,995,176-2,001,470](https://uswest.ensembl.org/Homo_sapiens/Location/View?db=core;g=ENSG00000130600;r=11:1995176-2001470) reverse strand | H19 imprinted maternally expressed transcript | NSCLC | Upregulated | Oncogene | 1.076 | 1.087 | Poor | Regulating proliferation in NSCLC cells | ^26^ |
| BLACAT1 | ENSG00000281406 | [Chromosome 1: 205,434,885-205,457,091](https://uswest.ensembl.org/Homo_sapiens/Location/View?db=core;g=ENSG00000281406;r=1:205434885-205457091) reverse strand | Bladder cancer associated transcript 1 | SCLC | Upregulated | Oncogene | 2.508 | 1.786 | Poor | ﻿Promoting proliferation, migration, and invasion in SCLC cells | ^27^ |
| NPTN-IT1 | ENSG00000281183 | [Chromosome 15: 73,567,012-73,569,294](https://uswest.ensembl.org/Homo_sapiens/Location/View?db=core;g=ENSG00000281183;r=15:73567012-73569294;t=ENST00000628401) reverse strand | NPTN intronic transcript 1; LncRNA-LET | NSCLC | Downregulated | Tumor suppressor | N/A | 0.358 | Good | ﻿Regulating proliferation and tumor progression *via* ﻿lncRNA-LET/Notch axis | ^28^ |
| LUCAT1 | ENSG00000248323 | [Chromosome 5: 91,054,834-91,314,547](https://uswest.ensembl.org/Homo_sapiens/Location/View?db=core;g=ENSG00000248323;r=5:91054834-91314547) reverse strand | Lung cancer associated transcript 1 | NSCLC | Upregulated | Oncogene | 1.073 | 1.085 | Poor | ﻿Regulating cell cycle and proliferation by inhibiting *p21* and *p57* expression | ^29^ |
| LINC00261 | ENSG00000259974 | [Chromosome 20: 22,547,671-22,578,642](https://uswest.ensembl.org/Homo_sapiens/Location/View?db=core;g=ENSG00000259974;r=20:22547671-22578642) reverse strand | Long intergenic non-protein coding RNA 261 | NSCLC | Downregulated | Tumor suppressor | 2.731 | 2.231 | Good | Regulating the malignant progression of NSCLC | ^30^ |
| FAM83H-AS1 | ENSG00000282685 | [Chromosome CHR_HSCHR8_3_CTG7: 143,721,147-143,733,344](https://uswest.ensembl.org/Homo_sapiens/Location/View?db=core;g=ENSG00000282685;r=CHR_HSCHR8_3_CTG7:143721147-143733344) forward strand | FAM83H antisense RNA 1 | LAD | Upregulated | Oncogene | N/A | N/A | Poor | ﻿Regulating cell proliferation and invasion through MET/EGFR signaling pathway | ^31^ |
| LINC00857 | ENSG00000237523 | [Chromosome 10: 80,207,372-80,219,657](https://uswest.ensembl.org/Homo_sapiens/Location/View?db=core;g=ENSG00000237523;r=10:80207372-80219657) forward strand | Long intergenic non-protein coding RNA 857 | LAD | Upregulated | Oncogene | N/A | N/A | Poor | ﻿Promoting cancer cell proliferation and invasion by regulating cell cycle | ^32^ |
| LINC00342 | ENSG00000232931 | [Chromosome 2: 95,807,052-95,835,003](https://uswest.ensembl.org/Homo_sapiens/Location/View?db=core;g=ENSG00000232931;r=2:95807052-95835003) reverse strand | Long intergenic non-protein coding RNA 342 | NSCLC | Upregulated | Oncogene | N/A | N/A | Poor | ﻿Promoting cell proliferation by inhibiting the expression of *p53* and PTEN | ^33^ |
| VPS9D1-AS1 | ENSG00000261373 | [Chromosome 16: 89,711,856-89,718,165](https://uswest.ensembl.org/Homo_sapiens/Location/View?db=core;g=ENSG00000261373;r=16:89711856-89718165) forward strand | VPS9D1 antisense RNA 1 | NSCLC | Upregulated | Oncogene | 2.871 | 2.281 | Poor | Promoting the malignant progression of NSCLC | ^34^ |
| FENDRR | ENSG00000268388 | [Chromosome 16: 86,474,529-86,509,099](https://uswest.ensembl.org/Homo_sapiens/Location/View?db=core;g=ENSG00000268388;r=16:86474529-86509099) reverse strand | FOXF1 adjacent non-coding developmental regulatory RNA | LAD | Downregulated | Tumor suppressor | N/A | N/A | Good | Regulating the malignant progression of LAD | ^35^ |
| SOX2OT | ENSMUSG00000105265 | [Chromosome 3: 34,104,270-34,682,619](https://uswest.ensembl.org/Mus_musculus/Location/View?db=core;g=ENSMUSG00000105265;r=3:34104270-34682619) forward strand | SOX2 overlapping transcript | NSCLC | Upregulated | Oncogene | 2.532 | 2.808 | Poor | ﻿Promoting cell proliferation by regulating cell cycle | ^36^ |
| HULC | ENSG00000285219 | [Chromosome 6: 8,435,568-9,294,133](https://uswest.ensembl.org/Homo_sapiens/Location/View?db=core;g=ENSG00000285219;r=6:8435568-9294133) forward strand | Hepatocellular carcinoma up-regulated long non-coding RNA | NSCLC | Upregulated | Oncogene | N/A | N/A | Poor | ﻿Promoting cell proliferation, migration and invasion *via* ﻿PI3K/  Akt signaling pathway | ^37^ |
| KCNQ1OT1 | ENSG00000269821 | [Chromosome 11: 2,608,328-2,699,994](https://uswest.ensembl.org/Homo_sapiens/Location/View?db=core;g=ENSG00000269821;r=11:2608328-2699994;t=ENST00000597346) reverse strand | KCNQ1 opposite strand/antisense transcript 1 | NSCLC | Upregulated in ﻿early-stage NSCLC | Tumor suppressor | N/A | N/A | Good | ﻿Inhibiting NSCLC cell proliferation and tumor growth | ^38^ |
| LncRNA-ATB | 114004396 | Chromosome 14 | Long non-coding RNA activated by TGF-β | NSCLC | Upregulated | Oncogene | N/A | N/A | Poor | ﻿Promoting NSCLC cell proliferation and metastasis | ^39^ |
| GAS6-AS1 | ENSG00000233695 | [Chromosome 13: 113,815,630-113,845,744](https://uswest.ensembl.org/Homo_sapiens/Location/View?db=core;g=ENSG00000233695;r=13:113815630-113845744) forward strand | GAS6 antisense RNA 1 | NSCLC | Downregulated | Tumor suppressor | 0.043 | 0.15 | Good | Regulating the malignant progression of NSCLC | ^40^ |
| RGMB-AS1 | ENSG00000246763 | [Chromosome 5: 98,769,618-98,773,469](https://uswest.ensembl.org/Homo_sapiens/Location/View?db=core;g=ENSG00000246763;r=5:98769618-98773469) reverse strand | RGMB antisense RNA 1 | LAD | Upregulated | Oncogene | N/A | N/A | Poor | ﻿Promoting LAD cell proliferation, migration and invasion | ^41^ |
| ZEB1-AS1 | ENSG00000237036 | [Chromosome 10: 31,206,278-31,320,447](https://uswest.ensembl.org/Homo_sapiens/Location/View?db=core;g=ENSG00000237036;r=10:31206278-31320447) reverse strand | ZEB1 antisense RNA 1 | NSCLC | Upregulated | Oncogene | 1.704 | 1.577 | Poor | Promoting the malignant progression of NSCLC | ^42^ |
| LINC00504 | ENSG00000248360 | [Chromosome 4: 14,470,465-14,888,169](https://uswest.ensembl.org/Homo_sapiens/Location/View?db=core;g=ENSG00000248360;r=4:14470465-14888169) reverse strand | Long intergenic non-protein coding RNA 504 | NSCLC | Upregulated | Oncogene | 3.261 | 2.895 | Poor | Promoting the malignant progression of NSCLC | ^43^ |
| ﻿NEAT1 | ENSG00000245532 | [Chromosome 11: 65,422,774-65,445,540](https://uswest.ensembl.org/Homo_sapiens/Location/View?db=core;g=ENSG00000245532;r=11:65422774-65445540) forward strand | Nuclear paraspeckle assembly transcript 1 | NSCLC | Upregulated | Oncogene | N/A | N/A | Poor | Promoting the malignant progression of NSCLC | ^44^ |
| GAS5 | ENSG00000234741 | [Chromosome 1: 173,858,559-173,868,882](https://uswest.ensembl.org/Homo_sapiens/Location/View?db=core;g=ENSG00000234741;r=1:173858559-173868882) reverse strand | Growth arrest specific 5 | NSCLC | Downregulated | Tumor suppressor | N/A | N/A | Good | Regulating NSCLC ﻿cell growth and apoptosis | ^45^ |
| HNF1A-AS1 | ENSG00000241388 | [Chromosome 12: 120,941,728-120,980,965](https://uswest.ensembl.org/Homo_sapiens/Location/View?db=core;g=ENSG00000241388;r=12:120941728-120980965) reverse strand | HNF1A antisense RNA 1 | NSCLC | Upregulated | Oncogene | 3.166 | 3.014 | Poor | Promoting the malignant progression of NSCLC | ^46^ |
| CYTOR | ENSG00000222041 | [Chromosome 2: 87,454,781-87,636,740](https://uswest.ensembl.org/Homo_sapiens/Location/View?db=core;g=ENSG00000222041;r=2:87454781-87636740) forward strand | ﻿Cytoskeleton regulator RNA; LINC00152 | LAD | Upregulated | Oncogene | N/A | N/A | Poor | Promoting LAD cell proliferation *via* regulating histone acetylation level | ^47^ |
| MALAT1 | ENSG00000251562 | [Chromosome 11: 65,497,688-65,506,516](https://uswest.ensembl.org/Homo_sapiens/Location/View?db=core;g=ENSG00000251562;r=11:65497688-65506516) forward strand | Metastasis associated lung adenocarcinoma transcript 1 | NSCLC | Upregulated | Oncogene | N/A | N/A | Poor | Inducing NSCLC cells migration and tumor growth | ^48^ |
| MEG3 | ENSG00000214548 | [Chromosome 14: 100,779,410-100,861,031](https://uswest.ensembl.org/Homo_sapiens/Location/View?db=core;g=ENSG00000214548;r=14:100779410-100861031) forward strand | Maternally expressed 3 | NSCLC | Downregulated | Tumor suppressor | 0.666 | 0.745 | Good | Regulating NSCLC ﻿cell growth | ^49^ |
| RFPL3S | ENSG00000205853 | [Chromosome 22: 32,359,886-32,382,106](https://uswest.ensembl.org/Homo_sapiens/Location/View?db=core;g=ENSG00000205853;r=22:32359886-32382106) reverse strand | RFPL3 antisense | LC | Upregulated | Oncogene | N/A | N/A | Poor | Promoting the malignant progression of LC | ^50^ |
| CERNA2 | ENSG00000285972 | [Chromosome 10: 84,167,228-84,172,093](https://uswest.ensembl.org/Homo_sapiens/Location/View?db=core;g=ENSG00000285972;r=10:84167228-84172093;t=ENST00000647830) reverse strand | Competing endogenous lncRNA 2 for microRNA let-7b; HOST2 | NSCLC | Upregulated | Oncogene | N/A | N/A | Poor | ﻿Promoting NSCLC cell ﻿viability, migration and invasion | ^51^ |

Abbreviations

AFAP1: Actin Filament Associated Protein 1

AGAP2: ArfGAP With GTPase Domain, Ankyrin Repeat And PH Domain 2

BRAF: B-Raf Proto-Oncogene, Serine/Threonine Kinase

CDKN1A: Cyclin Dependent Kinase Inhibitor 1A

CDKN2B: Cyclin Dependent Kinase Inhibitor 2B

EGFR: Epidermal Growth Factor Receptor

EMT: ﻿Epithelial-mesenchymal transition

FAM83H: Family With Sequence Similarity 83 Member H

FOXF1: Forkhead Box F1

GAS6: Growth Arrest Specific 6

HCC: Hepatocellular carcinoma

HNF1A: HNF1 Homeobox A

HOXA5: Homeobox A5

HR:﻿ Hazard ratio

IGF1R: Insulin Like Growth Factor 1 Receptor

KCNQ1: Potassium Voltage-Gated Channel Subfamily Q Member 1

LAD: Lung adenocarcinoma

LC: Lung cancer

LncRNA-LET: LncRNA-Low Expression in Tumor

MET: Mesenchymal-Epithelial Transition

MIR31: MicroRNA 31

MMP2: Matrix Metallopeptidase 2

MMP9: Matrix Metallopeptidase 9

NF-YA: Nuclear Transcription Factor Y Subunit Alpha

NPTN: Neuroplastin

NSCLC: ﻿Non-small cell lung cancer

N/A: Not available

RFPL3: Ret Finger Protein Like 3

PTEN: Phosphatase And Tensin Homolog

PVT1: Plasmacytoma variant translocation 1

RGMB: Repulsive Guidance Molecule BMP Co-Receptor B

RPS24: Ribosomal Protein S24

SBF2: SET Binding Factor 2

SCLC: ﻿Small cell lung cancer

SLC16A1: Solute Carrier Family 16 Member 1

SOX2: SRY-Box Transcription Factor 2

VPS9D1: VPS9 Domain Containing 1

ZEB1: Zinc Finger E-Box Binding Homeobox 1

Supplementary References

1. Xu YH, Tu JR, Zhao TT, Xie SG, Tang SB. Overexpression of lncRNA EGFRAS1 is associated with a poor prognosis and promotes chemotherapy resistance in nonsmall cell lung cancer. *Int J Oncol.* 2019;54(1):295-305.

2. Fu Y, Li C, Luo Y, Li L, Liu J, Gui R. Silencing of Long Non-coding RNA MIAT Sensitizes Lung Cancer Cells to Gefitinib by Epigenetically Regulating miR-34a. *Front Pharmacol.* 2018;9:82.

3. Luo CL, Xu ZG, Chen H, et al. LncRNAs and EGFRvIII sequestered in TEPs enable blood-based NSCLC diagnosis. *Cancer Manag Res.* 2018;10:1449-1459.

4. Liu HY, Lu SR, Guo ZH, et al. lncRNA SLC16A1-AS1 as a novel prognostic biomarker in non-small cell lung cancer. *J Investig Med.* 2020;68(1):52-59.

5. Huang C, Liu S, Wang H, Zhang Z, Yang Q, Gao F. LncRNA PVT1 overexpression is a poor prognostic biomarker and regulates migration and invasion in small cell lung cancer. *American journal of translational research.* 2016;8(11):5025.

6. Qin J, Ning H, Zhou Y, Hu Y, Yang L, Huang R. LncRNA MIR31HG overexpression serves as poor prognostic biomarker and promotes cells proliferation in lung adenocarcinoma. *Biomed Pharmacother.* 2018;99:363-368.

7. Liu X-h, Liu Z-l, Sun M, Liu J, Wang Z-x, De W. The long non-coding RNA HOTAIR indicates a poor prognosis and promotes metastasis in non-small cell lung cancer. *BMC Cancer.* 2013;13(1).

8. Xie X, Liu H-T, Mei J, et al. LncRNA HMlincRNA717 is down-regulated in non-small cell lung cancer and associated with poor prognosis. *International journal of clinical and experimental pathology.* 2014;7(12):8881.

9. Nie FQ, Zhu Q, Xu TP, et al. Long non-coding RNA MVIH indicates a poor prognosis for non-small cell lung cancer and promotes cell proliferation and invasion. *Tumour Biol.* 2014;35(8):7587-7594.

10. Lin L, Gu ZT, Chen WH, Cao KJ. Increased expression of the long non-coding RNA ANRIL promotes lung cancer cell metastasis and correlates with poor prognosis. *Diagn Pathol.* 2015;10:14.

11. Han L, Zhang EB, Yin DD, et al. Low expression of long noncoding RNA PANDAR predicts a poor prognosis of non-small cell lung cancer and affects cell apoptosis by regulating Bcl-2. *Cell Death Dis.* 2015;6:e1665.

12. Zhao Q, Li L, Zhang L, et al. Over-expression of lncRNA SBF2-AS1 is associated with advanced tumor progression and poor prognosis in patients with non-small cell lung cancer. *Eur Rev Med Pharmacol Sci.* 2016;20(14):3031-3034.

13. Luo J, Tang L, Zhang J, et al. Long non-coding RNA CARLo-5 is a negative prognostic factor and exhibits tumor pro-oncogenic activity in non-small cell lung cancer. *Tumour Biol.* 2014;35(11):11541-11549.

14. Chen S, Wu H, Lv N, et al. LncRNA CCAT2 predicts poor prognosis and regulates growth and metastasis in small cell lung cancer. *Biomed Pharmacother.* 2016;82:583-588.

15. Chen J, Hu L, Wang J, et al. Low Expression LncRNA TUBA4B is a Poor Predictor of Prognosis and Regulates Cell Proliferation in Non-Small Cell Lung Cancer. *Pathol Oncol Res.* 2017;23(2):265-270.

16. Shen Q, Wang H, Xu S. LncRNA GHET1 predicts a poor prognosis of the patients with non-small cell lung cancer. *Eur Rev Med Pharmacol Sci.* 2018;22(8):2328-2333.

17. Wu B, Zhang X, Li X, Jiang L, He F. Long non-coding RNA Loc344887 is a potential prognostic biomarker in non-small cell lung cancer. *Eur Rev Med Pharmacol Sci.* 2017;21(17):3808-3812.

18. Gong J, Zhang H, He L, Wang L, Wang J. Increased Expression of Long Non-Coding RNA BCAR4 Is Predictive of Poor Prognosis in Patients with Non-Small Cell Lung Cancer. *Tohoku J Exp Med.* 2017;241(1):29-34.

19. Jing H, Qu X, Liu L, Xia H. A Novel Long Noncoding RNA (lncRNA), LL22NC03-N64E9.1, Promotes the Proliferation of Lung Cancer Cells and is a Potential Prognostic Molecular Biomarker for Lung Cancer. *Med Sci Monit.* 2018;24:4317-4323.

20. Qu C, Sun Q, Zhang F, Jia Y. Long non-coding RNA ROR is a novel prognosis factor associated with non-small-cell lung cancer progression. *Eur Rev Med Pharmacol Sci.* 2017;21(18):4087-4091.

21. Li M, Zhang W, Zhang S, Wang C, Lin Y. PART1 expression is associated with poor prognosis and tumor recurrence in stage I-III non-small cell lung cancer. *J Cancer.* 2017;8(10):1795-1800.

22. Yin D, Lu X, Su J, et al. Long noncoding RNA AFAP1-AS1 predicts a poor prognosis and regulates non-small cell lung cancer cell proliferation by epigenetically repressing p21 expression. *Mol Cancer.* 2018;17(1):92.

23. Wang X, Li G, Luo Q, Xie J, Gan C. Integrated TCGA analysis implicates lncRNA CTB-193M12.5 as a prognostic factor in lung adenocarcinoma. *Cancer Cell Int.* 2018;18:27.

24. Sun M, Liu X-H, Wang K-M, et al. Downregulation of BRAF activated non-coding RNA is associated with poor prognosis for non-small cell lung cancer and promotes metastasis by affecting epithelial-mesenchymal transition. *Molecular Cancer.* 2014;13(1).

25. He X, Liu Z, Su J, et al. Low expression of long noncoding RNA CASC2 indicates a poor prognosis and regulates cell proliferation in non-small cell lung cancer. *Tumour Biol.* 2016;37(7):9503-9510.

26. Zhang E, Li W, Yin D, et al. c-Myc-regulated long non-coding RNA H19 indicates a poor prognosis and affects cell proliferation in non-small-cell lung cancer. *Tumour Biol.* 2016;37(3):4007-4015.

27. Chen W, Hang Y, Xu W, et al. BLACAT1 predicts poor prognosis and serves as oncogenic lncRNA in small-cell lung cancer. *J Cell Biochem.* 2018.

28. Li S, Zhao H, Li J, Zhang A, Wang H. Downregulation of long non-coding RNA LET predicts poor prognosis and increases Notch signaling in non-small cell lung cancer. *Oncotarget.* 2018;9(1):1156-1168.

29. Sun Y, Jin S-D, Zhu Q, et al. Long non-coding RNA LUCAT1 is associated with poor prognosis in human non-small cell lung cancer and regulates cell proliferation via epigenetically repressing p21 and p57 expression. *Oncotarget.* 2017;8(17):28297-28311.

30. Liu Y, Xiao N, Xu S. Decreased expression of long non-coding RNA LINC00261 is a prognostic marker for patients with non-small cell lung cancer: a preliminary study. *Eur Rev Med Pharmacol Sci.* 2017;21(24):5691-5695.

31. Zhang J, Feng S, Su W, et al. Overexpression of FAM83H-AS1 indicates poor patient survival and knockdown impairs cell proliferation and invasion via MET/EGFR signaling in lung cancer. *Sci Rep.* 2017;7:42819.

32. Wang L, He Y, Liu W, et al. Non-coding RNA LINC00857 is predictive of poor patient survival and promotes tumor progression via cell cycle regulation in lung cancer. *Oncotarget.* 2016;7(10):11487-11499.

33. Tang H, Zhao L, Li M, Li T, Hao Y. Investigation of LINC00342 as a poor prognostic biomarker for human patients with non-small cell lung cancer. *J Cell Biochem.* 2019;120(4):5055-5061.

34. Tan J, Yang L. Long noncoding RNA VPS9D1-AS1 overexpression predicts a poor prognosis in non-small cell lung cancer. *Biomed Pharmacother.* 2018;106:1600-1606.

35. Herrera-Merchan A, Cuadros M, Rodriguez MI, et al. The value of lncRNA FENDRR and FOXF1 as a prognostic factor for survival of lung adenocarcinoma. *Oncotarget.* 2020;11(13):1172.

36. Hou Z, Zhao W, Zhou J, et al. A long noncoding RNA Sox2ot regulates lung cancer cell proliferation and is a prognostic indicator of poor survival. *Int J Biochem Cell Biol.* 2014;53:380-388.

37. Zhang J, Lu S, Zhu J-F, Yang K-P. Up-regulation of lncRNA HULC predicts a poor prognosis and promotes growth and metastasis in non-small cell lung cancer. *Int J Clin Exp Pathol.* 2016;9(12):12415-12422.

38. Sun X, Xin Y, Wang M, et al. Overexpression of long non-coding RNA KCNQ1OT1 is related to good prognosis via inhibiting cell proliferation in non-small cell lung cancer. *Thorac Cancer.* 2018;9(5):523-531.

39. Ke L, Xu SB, Wang J, Jiang XL, Xu MQ. High expression of long non-coding RNA ATB indicates a poor prognosis and regulates cell proliferation and metastasis in non-small cell lung cancer. *Clin Transl Oncol.* 2017;19(5):599-605.

40. Han L, Kong R, Yin DD, et al. Low expression of long noncoding RNA GAS6-AS1 predicts a poor prognosis in patients with NSCLC. *Med Oncol.* 2013;30(4):694.

41. Li P, Zhang G, Li J, et al. Long Noncoding RNA RGMB-AS1 Indicates a Poor Prognosis and Modulates Cell Proliferation, Migration and Invasion in Lung Adenocarcinoma. *PLoS One.* 2016;11(3):e0150790.

42. Xie J, Wu Y, Bian X, Chen D, Gui Q, Huang J. Increased expression of lncRNA ZEB1-AS1 in non-small cell lung cancer is associated with poor prognosis. *International Journal of Clinical and Experimental Pathology.* 2018;11(7):3703.

43. Ma H, Wang L, Li W, Guo H, Wu Y, Li X. Upregulation of LINC00504 is associated with aggressive progression and poor prognosis in non-small cell lung cancer. *Eur Rev Med Pharmacol Sci.* 2020;24(2):699-703.

44. Yang C, Li Z, Li Y, et al. Long non-coding RNA NEAT1 overexpression is associated with poor prognosis in cancer patients: a systematic review and meta-analysis. *Oncotarget.* 2016;8(2):2672-2680.

45. Kamel LM, Atef DM, Mackawy AMH, Shalaby SM, Abdelraheim N. Circulating long non-coding RNA GAS5 and SOX2OT as potential biomarkers for diagnosis and prognosis of non-small cell lung cancer. *Biotechnol Appl Biochem.* 2019;66(4):634-642.

46. Ma Y, Liang T, Li C, Li Y, Jin S, Liu Y. Long non-coding RNA HNF1A-AS1 up-regulation in non-small cell lung cancer correlates to poor survival. *Eur Rev Med Pharmacol Sci.* 2016;20(23):4858-4863.

47. Feng S, Zhang J, Su W, et al. Overexpression of LINC00152 correlates with poor patient survival and knockdown impairs cell proliferation in lung cancer. *Sci Rep.* 2017;7(1):2982.

48. Schmidt LH, Spieker T, Koschmieder S, et al. The long noncoding MALAT-1 RNA indicates a poor prognosis in non-small cell lung cancer and induces migration and tumor growth. *J Thorac Oncol.* 2011;6(12):1984-1992.

49. Zhang Z, Liu T, Wang K, et al. Down-regulation of long non-coding RNA MEG3 indicates an unfavorable prognosis in non-small cell lung cancer: Evidence from the GEO database. *Gene.* 2017;630:49-58.

50. Liu Z, Ning Z, Lu H, et al. Long non-coding RNA RFPL3S is a novel prognostic biomarker in lung cancer. *Oncol Lett.* 2020;20(2):1270-1280.

51. Wang D, Zhao Y, Zhao Z, Li Y, Liu L, Chen G. Long non-coding RNA HOST2 predicts poor prognosis and promotes cell proliferation and invasion in non-small cell lung cancer. *INTERNATIONAL JOURNAL OF CLINICAL AND EXPERIMENTAL MEDICINE.* 2018;11(1):157-165.
